# The Impact of APP on Alzheimer-like Pathogenesis and Gene Expression in Down Syndrome iPSC-Derived Neurons

**DOI:** 10.1016/j.stemcr.2018.05.004

**Published:** 2018-05-31

**Authors:** Dmitry A. Ovchinnikov, Othmar Korn, Isaac Virshup, Christine A. Wells, Ernst J. Wolvetang

**Affiliations:** 1 Australian Institute for Bioengineering and Nanotechnology, University of Queensland, Brisbane, QLD 4072, Australia; 2 Centre for Stem Cell Systems, MDHS, University of Melbourne, Melbourne, VIC 3010, Australia

**Keywords:** beta-amyloid, iPSC, Down syndrome, Hsa21 trisomy, CRISPR/Cas9, cortical neurogenesis, gene expression profiling, tau phosphorylation

## Abstract

Early-onset Alzheimer disease (AD)-like pathology in Down syndrome is commonly attributed to an increased dosage of the amyloid precursor protein (APP) gene. To test this in an isogenic human model, we deleted the supernumerary copy of the *APP* gene in trisomic Down syndrome induced pluripotent stem cells or upregulated APP expression in euploid human pluripotent stem cells using CRISPRa. Cortical neuronal differentiation shows that an increased APP gene dosage is responsible for increased β-amyloid production, altered Aβ42/40 ratio, and deposition of the pyroglutamate (E3)-containing amyloid aggregates, but not for several tau-related AD phenotypes or increased apoptosis. Transcriptome comparisons demonstrate that *APP* has a widespread and temporally modulated impact on neuronal gene expression. Collectively, these data reveal an important role for APP in the amyloidogenic aspects of AD but challenge the idea that increased APP levels are solely responsible for increasing specific phosphorylated forms of tau or enhanced neuronal cell death in Down syndrome-associated AD pathogenesis.

## Highlights

• Normalization of APP copy number in Down syndrome (DS) iPSCs
• APP controls neuronal amyloid levels and pyroglutamate (pE3) accumulation
• APP in DS neurons does not affect levels of neurotoxic tau species or apoptosis
• APP gene dosage has stage-specific and genome-wide effects on gene expression

## Graphical Abstract

**Figure:**
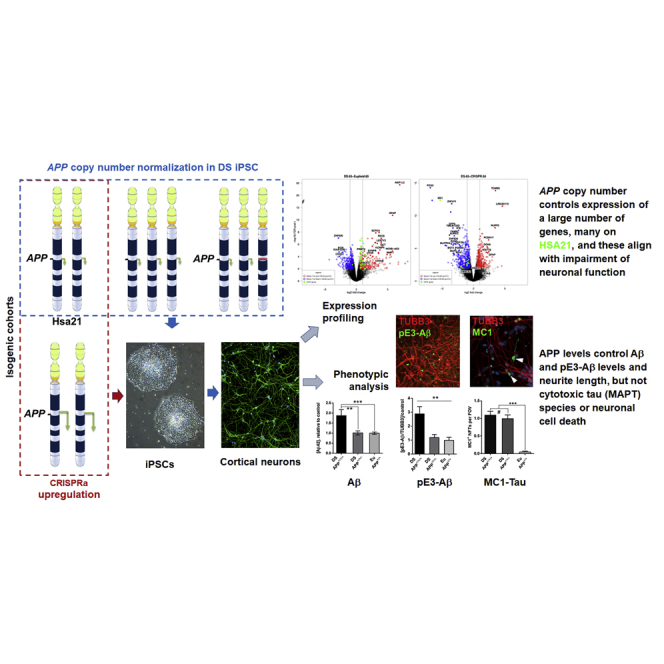

## Teaser

Wolvetang and colleagues used CRISPR/Cas9 technologies to manipulate the copy number and expression of the amyloid precursor protein (APP) gene in Down syndrome and corresponding euploid pluripotent stem cells. They demonstrate that APP modulates the expression of a surprisingly large cohort of genes and dictates Aβ42/40 ratio and pyroglutamate-E3 foci but does not affect hyperphosphorylated forms of tau associated with Alzheimer disease or neuronal cell death of *in vitro* generated cortical neurons.

## Introduction

The *APP* gene encodes the amyloid precursor protein (APP) and is located on chromosome 21. Increased dosage of this gene results in an elevated expression of APP in Down syndrome (DS; trisomy 21) tissues (Oyama et al., 1994). This is thought to increase the levels of β-amyloid (Aβ), a cleavage product of APP that aggregates upon misfolding, accumulates in plaques in the brains of people with Alzheimer disease (AD) and DS (Braak and Braak, 1994), and in turn is assumed to underlie the development of early-onset, highly penetrant, AD-like pathology in individuals with DS (Decourt et al., 2013). Aβ aggregation was previously linked to tau hyperphosphorylation, defective synapse function, oxidative stress, and increased neuronal cell death (Spires-Jones and Hyman, 2014). Consistent with these observations, three cases of partial trisomy of chromosome 21 that exclude the *APP* locus showed no evidence of early-onset AD (EOAD) (Korbel et al., 2009) or neurodegeneration at an advanced age (Doran et al., 2017). Similarly, individuals with a rare familial duplication of the *APP* locus develop EOAD, although this is more akin to vascular dementia than classical AD (Rovelet-Lecrux et al., 2007, Sleegers et al., 2006). Individuals with DS can, nevertheless, carry large plaque loads without overt AD signs (Vemuri et al., 2010), challenging a direct causal relationship between *APP* triplication and EOAD in DS. Indeed, the penetrance and expressivity of disease phenotypes, including AD-like pathology, vary between DS individuals, and this has been attributed to the presence of modifier alleles on Hsa21 (e.g., *DYRK1A*, *BACE2*, *miR-155*) or other chromosomes, such as *APOE* (Sherman et al., 2007). Several groups have generated induced pluripotent stem cells (iPSCs) from individuals with DS (e.g., Shi et al., 2012). We (Briggs et al., 2013) and others (Murray et al., 2015) have previously found that nuclear reprogramming permits the isolation of isogenic euploid (Hsa21-disomic) iPSCs from otherwise fully Hsa21-trisomic DS subjects. DS iPSC-derived cortical neurons were previously shown to exhibit increased production of Aβ42, and hyperphosphorylation and redistribution of tau (Chang et al., 2015), suggesting that DS iPSC-derived cortical neuronal cultures can recapitulate aspects of AD neuropathology *in vitro* (Shi et al., 2012).

To elucidate the role of APP in EOAD in DS without potential confounding effects of modifier alleles, we manipulated APP dosage and expression in isogenic DS or euploid iPSC backgrounds; subjected these cell lines to prolonged cortical differentiation; and analyzed gene expression-, amyloid-, and tau-associated changes. Our data reveal APP gene dosage in DS has neurodevelopmental stage-specific, genome-wide gene regulatory effects and affects the Aβ42/Aβ40 ratio and pyroglutamate aggregates but does not alter a range of tau-phosphorylation events, abundance of neurofibrillary tangle (NFT)-like tau aggregates, or neuronal cell death.

## Results

### Generation of APP Copy-Number-Normalized DS iPSCs and Doxycycline-Inducible *APP*-Overexpressing Human Embryonic Stem Cells

**Figure 1:**
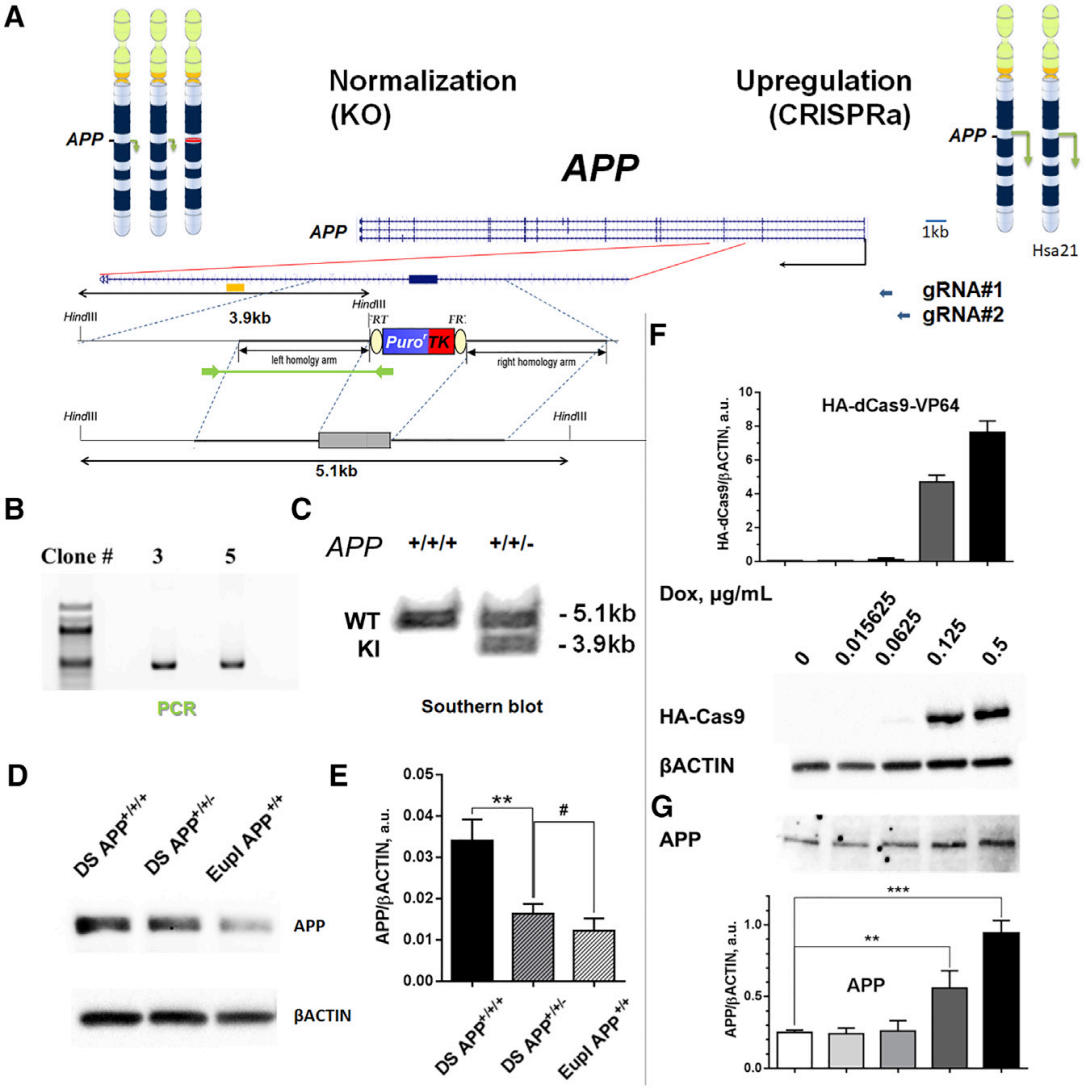
Manipulating *APP* Levels in Human Pluripotent Stem Cells Using CRISPR/Cas9-Aided Approaches (A) Vector design for targeting exon 3 of the *APP* gene. Green is PCR product, yellow rectangle is location of the Southern probe. gRNA, guide RNA; KO, knockout. (B) Targeted allele-specific PCR. (C) Southern blot showing *APP* targeted allele in DS18 iPSC. KI, knockin; WT, wild-type. (D and E) (D) Inactivationof one of the three *APP* alleles in day 45 DS iPSC-derived neurons reduces APP protein expression to isogenic euploid control levels (quantified in E, N = 3). (F) Doxycycline induced upregulation of HA-tagged dCas9-VP64 in Gen22::TRE-dCas9-VP64 with HA antibody. (G) Doxycycline induced APP protein expression in Gen22::TRE-dCas9-VP64 hESC (APP gRNA#1 shown). ^∗∗^p < 0.01, ^∗∗∗^p < 0.001, #non-significant; n = 3, n = 3 for qPCR and western blots. Means ± SEM values shown.

To address the role of the supernumerary copy of the APP gene in AD-like neuropathology in DS, we deleted exon 3 of one of the APP alleles (Figure 1A) in a previously characterized footprint-free DS iPSC line, clone C11DS (Briggs et al., 2013) and this was confirmed by genomic PCR (Figure 1B) and Southern blotting (Figure 1C). As expected, the ∼1.5–2-fold increase in APP protein expression observed in neuronally differentiated DS APP^+/+/+^ iPSC was “normalized” to euploid levels in the isogenic CRISPR-targeted APP^+/+/−^ DS iPSC-derived neuronal cultures (Figures 1D and 1E), and this was maintained during prolonged neuronal differentiation (up to 90 days tested; Figure S3A).

APP overexpression in a euploid (Hsa21-disomic) background was achieved through lentiviral delivery of a doxycycline-inducible CRISPRa-driven system. We isolated a clonal line that displays tightly controlled dox-inducible HA-dCAS9-VP64 (Figure 1F) and APP (Figure 1G) expression, following lentiviral delivery of guide RNAs (gRNAs) that target the APP promoter (Figure S1J and Supplemental Experimental Procedures). The APP^+/+/−^ iPSC and the Genea22::HA-dCAS9-VP64 line were devoid of chromosomal abnormalities (SNP arrays) and showed the hallmarks of pluripotent stem cells (Figure S1).

Neurogenic cultures derived from all six isogenic iPSC clones (2 DS APP^+/+/+^, 1 DS APP^+/+/−^, and three euploid APP^+/+^ lines) displayed similar cortical neuronal trajectories with little contamination of non-neuronal cell types, and were transcriptionally most similar to the frontal cortex of a 16–18-week fetal brain (Figure S2A). Temporal changes in *TUBB3* and *TBR2* mRNA expression (Figure S2B), and immune-fluorescent detection of pan-neuronal markers TUBB3 and NeuN and astrocyte marker GFAP (Figure S2C), indicates comparable neuronal differentiation trajectories of all six isogenic lines. This is supported by the transcriptome-based temporal and regional staging of the neuronal cultures using CoNTExT (Figure S2), although a small decrease in cortical layer markers SATB2 and TBR1 (Figure S2C’) was detected in DS samples.

### *APP* Copy Number Has a Significant Impact on the Transcriptome of DS Cortical Neuronal Cultures

**Figure 2:**
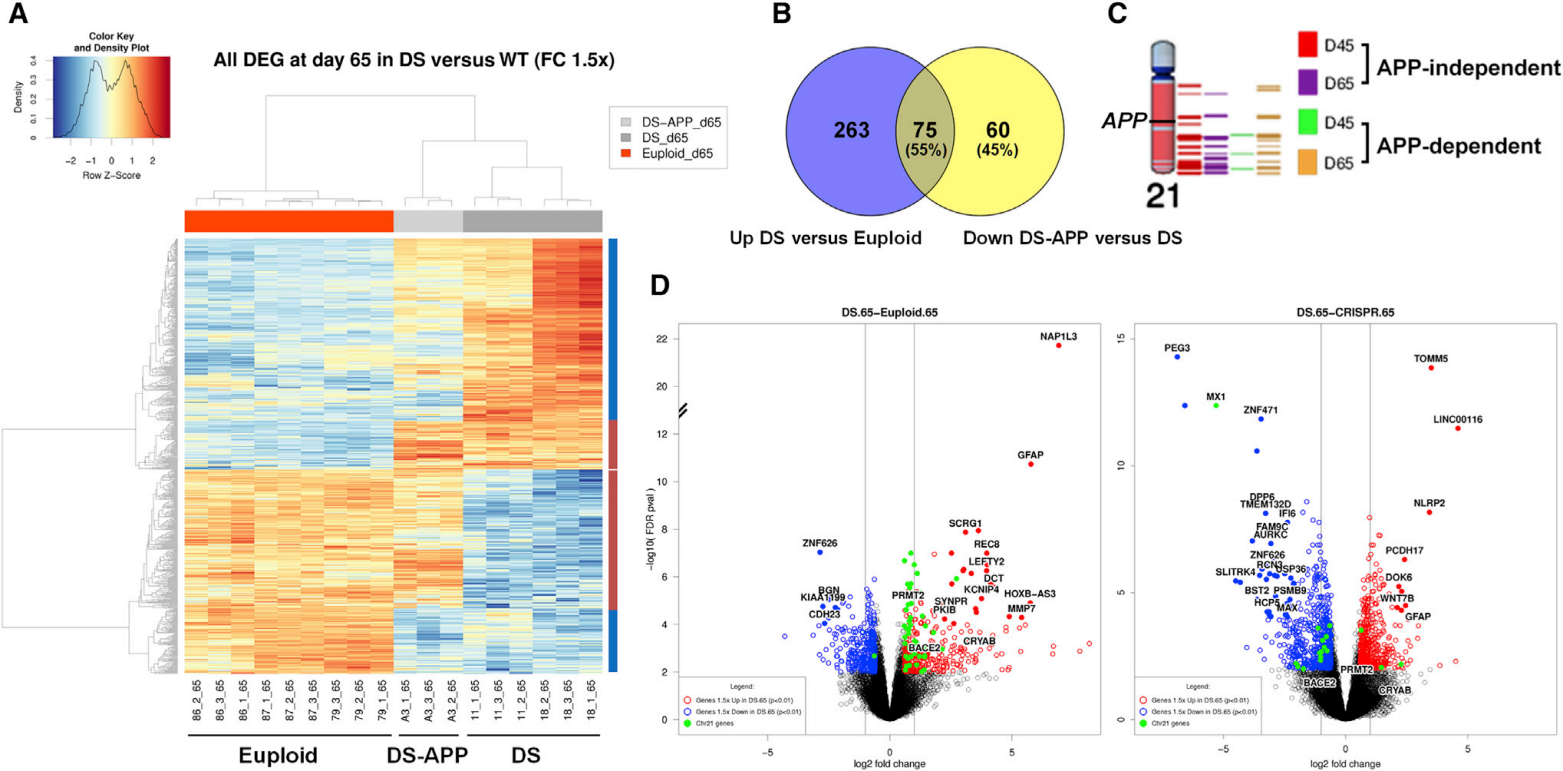
Transcriptome Profiling of Isogenic iPSC-Derived Neuronal Cultures at Day 65 of Differentiation (A) Hierarchical probe clustering-based heatmaps (with limits set at 1.5-fold changes and p < 0.01) in day 65 neuronal cultures from three euploid, two DS, and one APP-normalized DS iPSC lines. (B) Venn diagram illustrating overlap between DEG lists from DS-euploid and DS APP copy-adjusted comparisons. (C) Graphic representation of Hsa21 genes significantly upregulated in APP-independent and APP-dependent manners at indicated days of differentiation. (D) Volcano plots exemplifying asymmetrical changes in gene expression levels at day 65, with a larger number of genes upregulated in DS versus euploid, including numerous Hsa21 genes (shown in green). Similarly, a large number of genes are downregulated upon inactivation of the third *APP* copy in day 65 DS iPSC neurons (right panel).

Hierarchical clustering of microarray transcriptome data from DS APP^+/+/+^ and isogenic euploid day 65 samples shows clustering is dictated by the presence of Hsa21 (Figure 2A). As expected, Hsa21 genes are highly overrepresented among significantly overexpressed transcripts (green dots in volcano plot in Figure 2D), and day 45 gene expression data show similar trends (Figures S2D and S2E). At both time points, chromosome 21 genes are significantly overrepresented among upregulated differentially expressed genes (DEGs) in Hsa21-trisomic cells (p < 0.0002), particularly those in the distal part of the long arm (Figure 2C). The relative contribution of Hsa21 genes diminishes from 22% to 15% from day 45 to 65 (see GEO dataset for chromosomal assignment details). DS and AD were identified as the top disease signatures (Table S1), and Ingenuity Pathway Analysis identified neurological signs, cognitive impairment, and abnormality of the cerebral cortex (Table S2). We next examined the transcriptome differences driven by the presence of the supernumerary APP gene copy in an isogenic Hsa21-trisomic context. Hierarchical clustering analyses revealed that the impact of the supernumerary *APP* copy number on the trisomy 21 transcriptome increases significantly from day 45 (Figure S2D) to day 65 (Figure 2A). By day 65 about half of all genes downregulated upon inactivation of the supernumerary *APP* gene in DS neurons were also abnormally upregulated in DS neurons (Figure 2B). This substantial impact of the APP gene on DS (Hsa21-trisomy)-associated gene expression changes in neurons particularly affects the expression of other Hsa21 genes (Figures 2C and 2D).

### APP Copy Number Dictates Aβ Levels, Presence of Pyroglutamate Aggregates, and Neurite Length of DS iPSC-Derived Cortical Neurons

**Figure 3:**
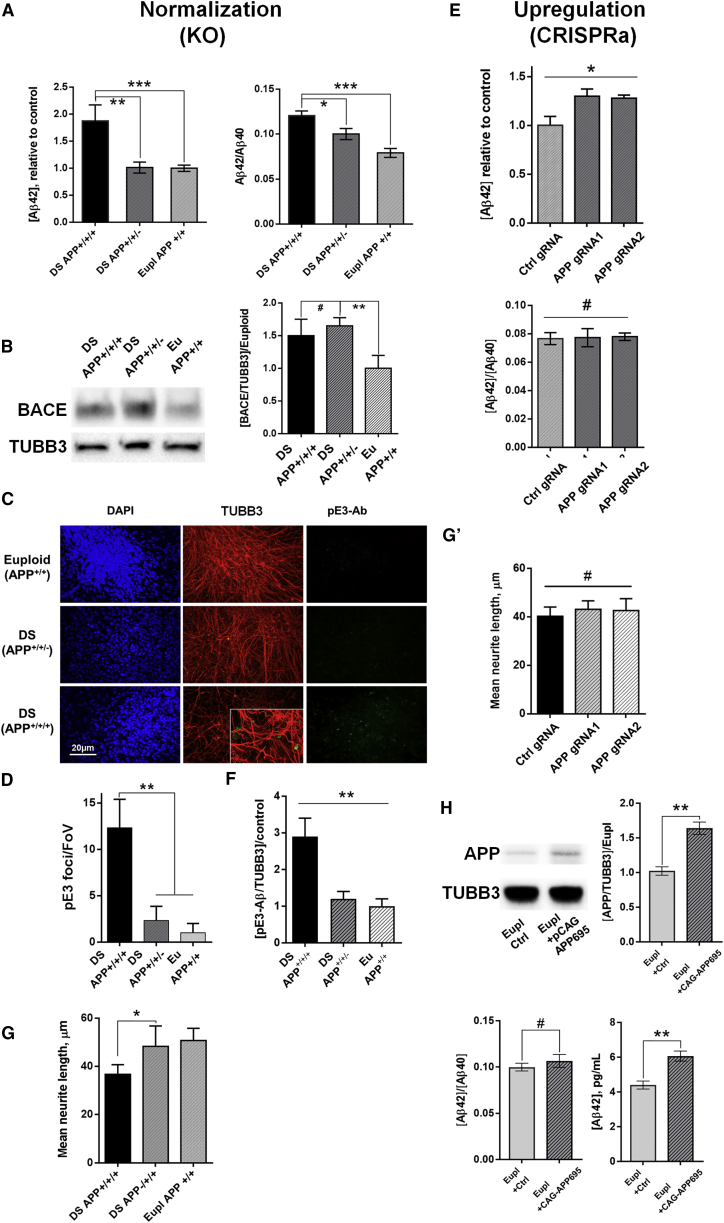
APP Levels Influence β-Amyloidogenic AD-like Phenotypes and Neurite Outgrowth in Neuronal Cultures (A) Levels of secreted Aβ42 and Aβ42/Aβ40 peptide ratios measured in medium conditioned by 90-day-old neuronal cultures of respective genotypes (N = 3). (B) Western blot detection of BACE protein in APP^+++^, APP^++–^ DS and isogenic euploid neurons. (C) Day 120+ DS neuronal cultures display more pE3-pyroglutamate-Aβ immunoreactive foci than euploid or APP copy-number-corrected DS cultures. (D) Quantification of the numbers of pE3-pyroglutamate foci (per field of view). (E) CRISPRa-driven overexpression of APP leads to an increase in Aβ42 levels but does not alter the Aβ42/Aβ40 ratio (day 90). (F) Quantification of pyroglutamate expression relative to TUBB3, as measured by western blotting. (G) The effect of APP copy number on neurite length in day 120 DS neurons and (G′) in euploid neurons with CRISPRa-upregulated APP. (H) Overexpression of APP in day 90 euploid neurons using APP695 ORF results in elevated Aβ42 levels but does not alter the Aβ42/Aβ40 ratio. Means ± SEM (N = 3). ^∗^p < 0.05, ^∗∗^p < 0.01, ^∗∗∗^p < 0.001, #non-significant. Scale bar in (C) represents 20 μm.

APP, when processed by the γ- and β-secretases, can generate the amyloidogenic Aβ42 peptide or the more abundant and non-cytotoxic Aβ40 peptide. Because these proteases are expressed (as confirmed by our microarray and qPCR data; Figure S3D), increased APP levels are expected to result in elevated neuronal Aβ amyloid production (Maulik et al., 2015) and an increased ratio of Aβ42/Aβ40 peptides. In day 90 DS iPSC-derived cortical neurons, we indeed detected an increased secretion of both Aβ42 (Figure 3A) and Aβ40 (Figure S3C) in the culture medium compared with APP^+/+^ (euploid) cells, and these levels were normalized to euploid levels in APP^+/+/−^ neuronal cultures. Upregulation of Aβ42 was also observed in the medium of Genea21 DS human embryonic stem cell (hESC)-derived neurons compared with the euploid Genea22 sibling hESC-derived neurons, but this was accompanied by little (day 45) to no (day 90) skewing of the Aβ42/Aβ40 ratio (Figure S3B). The correction of APP gene dosage (APP^+/+/−^ DS iPSC) reversed the elevated Aβ42/Aβ40 ratio observed in DS iPSC-derived neuronal cultures (Figure 3A), albeit only partially, suggesting APP expression may indirectly affect the activity of the processing machinery. Indeed, mRNA and protein levels of β-secretase BACE2, an Hsa21 gene already elevated in day 65 DS neurons (and DS brains; e.g., Cheon et al., 2008), becomes even further elevated in DS APP^+/+/−^ neurons (Figure 3B). Since BACE2 activity potentially reduces the substrate pool for BACE1 (Sun et al., 2006), this may account for the reduction in Aβ40 and thus the only partially normalized Aβ42/Aβ40 ratio observed in APP copy-number-normalized DS neurons (Figure S3E). Inhibition of BACE activity with 1 or 10 nM verubecestat reduced Aβ42 production (Figure S3F) and Aβ42/Aβ40 ratios in neuronal cultures from APP^+/+/+^ DS, APP^+/+/−^ DS, and isogenic euploid iPSCs (Figure S3E), confirming that BACE activity is responsible.

We next show that increasing the expression of APP in Genea022::TRE-dCas9-VP64 hESC-derived day 90 neurons was sufficient to increase both secreted Aβ42 and Aβ40 peptide levels but did not alter the Aβ42/Aβ40 ratio (Figure 3E). In euploid iPSC (Eu79)-derived neurons, 1.5-fold overexpression of APP using an APP695-expressing plasmid similarly upregulated Aβ42 peptide levels but also did not change the Aβ42/Aβ40 ratio (Figure 3H).

Collectively these data show that APP copy number is necessary for increased Aβ42 levels and skewing of the Aβ42/Aβ40 ratio in DS neurons, and that increasing APP expression in euploid neurons is sufficient to increase Aβ42 peptide levels but does not alter the Aβ42/Aβ40 ratio in this model.

Oligomeric Aβ42 is considered to be a key cytotoxic agent in AD, particularly after it heterodimerizes with N-terminally truncated pyroglutamylated Aβ (Gunn et al., 2010). This further processed species is more aggregation prone and can make up half of the amyloid load in AD brains (Gunn et al., 2016). We detected increased numbers of pyroglutamate (pE3)-positive foci in 120 day DS iPSC-derived neuronal cultures (Figure 3C) and increased protein expression by western blot (Figures 3D and S3G), which were both reduced to near-euploid levels upon removal of the supernumerary copy of the APP gene. CRISPRa-driven overexpression of *APP* in euploid Gen22 hESC was not sufficient to cause an appreciable increase in the pyroglutamate (pE3) levels (Figure S3H).

APP is also known to play a role in regulation of the outgrowth of neuronal processes. N-terminally processed extracellular fragments (e.g., sAPPα) can enhance neurite outgrowth, whereas C-terminally derived forms can exert opposite effects (Trazzi et al., 2013, Young-Pearse et al., 2008). Normalization of *APP* copy number in a DS chromosomal context increased mean neurite length by ∼18% (day 120 neurons), to a size similar to that of isogenic euploid controls (Figure 3G), suggesting that in DS the extra copy of APP limits neurite outgrowth. CRISPRa-directed upregulation of *APP* in euploid neurons did not increase neurite length (Figure 3G’).

### *APP* and Aβ42 Levels Do Not Directly Control Neuronal Cell Death or Tau-Hyperphosphorylation Sites Linked to Neurodegeneration

**Figure 4:**
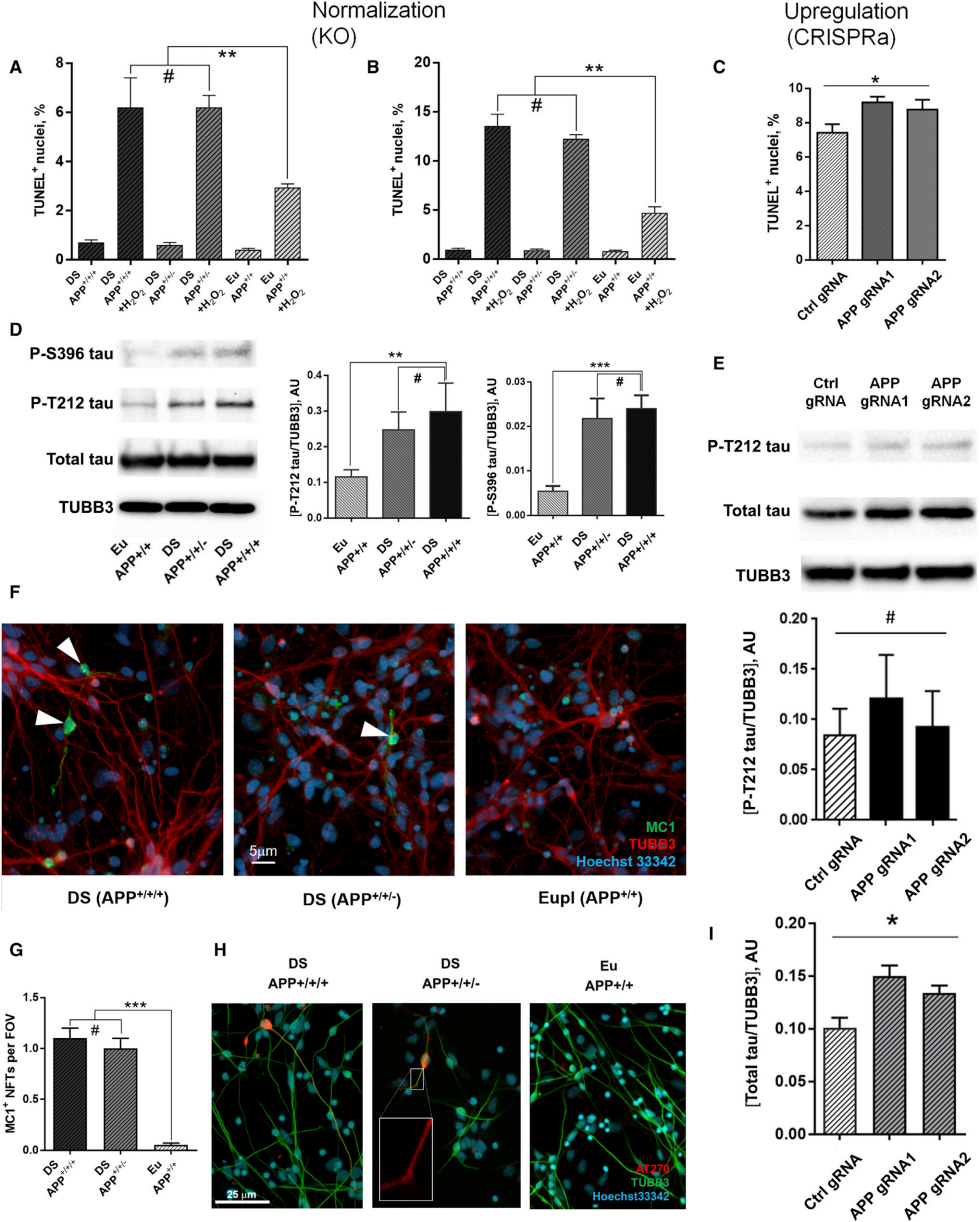
Hyperphosphorylation of Tau and Apoptosis Are Not Controlled by the Levels of *APP* Gene Expression (A) Hydrogen peroxide-induced cell death measured by fluorescence-activated cell sorting of TUNEL-labeled cells in DS neuronal cultures at days 45. (B and C) (B) Day 90 DS cultures and (C) day 90 Gen22::TRE-dCas9-VP64 hESC-derived neurons (gRNAs as labeled). (D) Western blots of P-T212, P-S396, and total Tau in day 90 DS, APP copy-number-corrected, and isogenic euploid neurons and their quantification relative to TUBB3. (E) Western blotting of day 90 Gen22::TRE-dCas9-VP64 hESC-derived neurons with T212 Tau antibody and quantification relative to total Tau. (F) Overlaid images of MC1^+^ (paired helical filament-containing) NFT-like aggregates (highlighted by arrowheads) in Hsa21-trisomic, APP copy-number-normalized, and euploid day 120 cortical neurons. (G) Quantification of the frequency of MC1^+^ NFT-containing neurons per field of view in D120 cortical neuronal cultures. (H) Detection of hyperphosphorylated tau accumulation using AT270 antibody in day 120 DS, APP copy-number-corrected, and isogenic euploid neurons. (I) Levels of total Tau protein in day 90 Genea22::TRE-dCas9-VP64 hESC-derived neurons overexpressing APP, relative to TUBB3. ^∗^p < 0.05, ^∗∗^p < 0.01, ^∗∗∗^p < 0.001, #non-significant. Values shown are means ± SEM of three experiments.

Aβ42 is thought to be responsible for increasing neuronal cell death by increasing oxidative stress (Manterola et al., 2013). Hydrogen peroxide-induced apoptosis, a measure of cellular resilience to oxidative stress, is indeed increased in primary DS fetus-derived neurons (Busciglio and Yankner, 1995). In agreement with these observations we find that day 90 DS neurons also exhibit increased hydrogen peroxide-induced apoptosis (Figures 4A and 4B). However, hydrogen peroxide sensitivity was not corrected by normalization of *APP* gene copy number in either day 45 (Figure 4A) or day 90 cultures (Figure 4B). Increasing the expression of APP in hESCs, which increases Aβ42 levels, only very modestly increased apoptosis in day 90 neuronal cultures (Figure 4C). We conclude that increased *APP* gene load, and increased Aβ42 and pE3 pyroglutamate levels, are not directly responsible for the increased oxidative stress-induced cell death of DS neurons under these conditions.

We next explored whether APP gene load and Aβ42 levels affect tau phosphorylation in day 90 neurons from isogenic DS, euploid, and APP-normalized DS iPSCs. To account for potential differences in tau protein levels themselves or altered neuronal content of the samples, the fractions of P-S396 and P-T212 tau (canonical sites implicated in AD) were normalized to either total tau or the neuronal marker βIII-tubulin (TUBB3). Irrespectively, P-S396 and P-T212 tau levels were significantly higher in DS neurons, but these were not reduced by normalization of APP gene dosage (Figure 4D). These data therefore strongly suggest that the increased levels of Aβ42 and skewed Aβ42/Aβ40 ratio in DS neuronal cultures are not directly responsible for increasing these tau-phosphorylation events, and this is consistent with our observation that normalization of APP also did not affect apoptosis, a process previously linked to tau phosphorylation (Wu et al., 2016). Immunofluorescent detection of hyperphosphorylated tau with the AT270 antibody revealed an increase in immunoreactivity in both cell bodies and axons in a subset of day 120 DS APP^+/+/+^ neurons compared with isogenic *APP*^*+/+*^ euploid neurons, but this was not reduced in DS *APP*^*+/+/−*^ cultures (Figure 4H). Similar data were obtained with the AT8 phospho-tau antibody (Figure S4A). Detection of pathological tau with the conformation-specific antibody MC1 revealed potential early NFT-like structures in day 120 DS neurons that were absent in isogenic euploid neurons but persisted in DS *APP*^*+/+/−*^ neuronal cultures (Figures 4F and 4G). CRISPRa upregulation of *APP* to DS levels in euploid hESCs over 90 days of neuronal differentiation did not lead to increased tau T212-phosphorylation (Figure 4E). This does lead to a modest upregulation of total tau in these neurons (Figure 4E), in agreement with previously reported effects of APP on tau proteostasis (Moore et al., 2015). Collectively our data suggest that the increased APP and Aβ42 levels do not affect the increased abundance of neuro-pathology-associated species of phosphorylated tau or apoptotic sensitivity of DS neurons.

## Discussion

In iPSC-derived DS neurons, APP mRNA and protein levels are ∼2-fold increased over the entire neuronal differentiation time course, in agreement with elevated gene dosage and previous reports (Buxbaum and Greengard, 1996). We show that inactivating one copy of the *APP* gene in a trisomy 21 background normalized APP, secreted levels of Aβ42, Aβ42/Aβ40 ratio (at least in part), and pyroglutamate (pE3)-containing Aβ levels and foci numbers to euploid levels. This indicates that increased expression of APP is directly responsible for these hallmark features of AD pathogenesis in this *in vitro* DS model. A considerable body of literature suggests that Aβ42 (either aggregated or in its monomeric form) directly or indirectly induces tau hyperphosphorylation and that this in turn leads to synaptic defects, neuronal dysfunction, and cell death (e.g., Gotz et al., 2001). Our data show that normalization of APP copy number and Aβ42 levels in DS cortical neurons does not reduce tau phosphorylation, nor does it affect the sensitivity of neurons to oxidative stress-induced apoptosis. Furthermore, overexpression of APP to physiologically relevant levels (1.5–2-fold) in euploid cortical neurons increased Aβ42 but was not sufficient to increase amyloid pyroglutamate E3 levels, or tau (T212) hyperphosphorylation, and only very moderately increased oxidative stress-induced apoptosis. APP overexpression by itself did not significantly skew the Aβ42/40 ratio, suggesting that other Hsa21 genes might attenuate APP processing.

These data challenge the notion of a direct linear relationship between APP/Aβ42 levels and tau pathology-induced neuronal cell death in this DS model. In agreement with this notion, mouse models that display typical Aβ pathology with amyloid plaque deposition, such as mice with a humanized *App* gene (Saito et al., 2014) or overexpression of wild-type APP protein (Cataldo et al., 2003), also did not exhibit tau pathology. In agreement with previous observations (Moore et al., 2015), APP gene copy number affects tau proteostasis, albeit modestly. Interestingly, we do not observe this in DS iPSC-derived neurons, suggesting compensatory effects of the dosage of other Hsa21 genes or other compensatory processes (Simon et al., 2012).

Collectively these data underline the need for a better understanding of the role(s) of other Hsa21 genes and elucidation of the intra-chromosomal and genome-wide gene regulatory networks they affect. Our gene expression data comparing isogenic euploid, DS, and APP copy-number-normalized DS neurons at days 45 and 65 of cortical differentiation highlight a number of important concepts in this regard. The initial idea that DS is exclusively driven by the overexpression of all Hsa21 genes or by genes that reside in a DS critical region (Korbel et al., 2009) has been superseded by more sophisticated models that take into account intra- and inter-chromosomal gene regulatory networks as well as epigenetic effects (Antonarakis, 2017). We now show that the number, identity, and expression levels of overexpressed Hsa21 genes in DS neurons vary during different temporal windows of neuronal differentiation, a concept well accepted in developmental biology. The corollary of this observation is that each cell type at each stage of development may be differentially affected by overexpression of specific combinations of Hsa21 genes (either directly, or indirectly by altering the expression of genes on other chromosomes, or by affecting the global epigenetic landscape), adding further complexity to understanding the etiology of DS-associated pathologies.

At later time points (days 90–120) we find that presence of a supernumerary APP copy decreases neurite length in a trisomic genetic background, in agreement with previous data in a DS mouse model (Trazzi et al., 2013). Our gene expression data reveal that a surprisingly large proportion of genes differentially expressed between euploid and DS neurons are controlled by APP copy number and that this proportion dramatically increases from day 45 to day 65. Remarkably, a disproportionally large fraction (∼10%) of these APP-controlled genes reside on chromosome 21 (see GEO dataset for details).

The molecular mechanism that mediates this unexpected gene regulatory role of APP remains unclear at this stage. It has been suggested that APP can function as a ligand, act as a (co)-receptor (Deyts et al., 2016), and can release a Notch-like intra-cellular domain of APP (AICD) that can alter transcription following dimerization with intra-cellular partners such as Fe65 and Tip60 (Cao and Sudhof, 2001, Chang and Suh, 2010). However, when we examined the cohort of genes identified using ChIP (chromatin immunoprecipitation)-on-chip in neuroblastoma cells overexpressing AICD and Fe65 (Muller et al., 2007), we found very limited overlap with our sets of APP copy-number-dependent up- or downregulated genes in DS neurons.

Our functional data indicating that tau phosphorylation and neuronal cell death were not affected by APP copy number or Aβ42 normalization are supported by our bioinformatics analyses that show that genes that change their behavior in an APP-dependent fashion in a Hsa21-trisomic background overlap with pathways related to abnormal nervous system development (Enrichr portal, MP0003632, p < 10^−16^) and abnormal brain morphology (MP0002152, p < 10^−11^) but not with AD (Table S1). Conversely, genes that are differentially expressed between DS and euploid neurons and overexpressed independently of the *APP* gene copy number show high congruence with gene sets upregulated in DS at both days 45 and 65 (false discovery rate [FDR]-corrected p values <6 × 10^−15^ and <3 × 10^−8^, respectively, see Table S1), indicating that, in this *in vitro* model, Hsa21 genes other than APP drive many of the DS and AD pathogenic processes. Gene expression comparisons of DS and euploid neurons identified AD as the top disease, whereas comparison of DS with DS APP aligns with the “abnormal brain development and morphology”-associated gene list (input datasets shown in Tables S1A and S4A). Collectively, our data reveal that the *APP* gene plays an important role in moderating the expression of genes in *trans*, many of which reside on chromosome 21 itself, and that this changes during the course of cortical neuronal differentiation *in vitro*. We further demonstrate that the supernumerary copy of *APP* is indeed responsible for increased Aβ42 and pyroglutamate-containing amyloid levels in DS but is not directly involved in stimulation of tau hyperphosphorylation or increased neuronal cell death in DS neuronal cultures.

Systematic CRISPR-assisted genome manipulation in DS iPSC should permit the further elucidation of the transcriptional and putative epigenetic modulatory effects of APP and the roles and interactions of other Hsa21 genes involved in AD pathogenesis in DS, and provide further insight into the complex molecular mechanisms and gene regulatory networks underlying AD pathogenesis in DS and the general population.

## Experimental Procedures

### Human Pluripotent Stem Cell Culture

Experiments were performed with approval by the University of Queensland Human Research Ethics Committee (HREC/09/QRCH/103, approval number 2015000667) and University of Queensland Animal Ethics Committee (AIBN/178/15/SCA/LEJEUNE/KACST).

Derivation of isogenic DS and euploid human iPSCs from skin fibroblasts was described (Briggs et al., 2013) and the near-isogenic hESC cell lines Genea021 and Genea022 (Dumevska et al., 2016a, Dumevska et al., 2016b) were obtained from Genea Biocells (San Diego, United States). Pluripotent cell cultures were maintained on Matrigel substrate (BD Biosciences, United States) in mouse embryonic fibroblast-conditioned knockout serum replacement (KOSR) medium supplemented with 100 μM β-mercaptoethanol and 100 ng/mL basic fibroblast growth factor (bFGF) (for iPSCs) or 50 ng/mL bFGF (for hESCs), and passaged by manual cutting or enzymatic dissociation using dispase as described (Ovchinnikov et al., 2015b).

### Generation of the Cortical Neurogenic Cultures

Simultaneous inhibition of transforming growth factor β/activin and bone morphogenetic protein/growth differentiation factor signaling pathways was used to generate neurogenic cultures (Briggs et al., 2013, Chambers et al., 2009). Production of the neuroprogenitor and neuronal populations was performed using conventional approaches (see Supplemental Experimental Procedures), and maturation for 90- and 120-day-old cultures was achieved by supplementing the differentiation medium from day 65 with 20 ng/mL brain-derived neurotrophic factor, 10 ng/mL glial cell-derived neurotrophic factor, 500 nM ascorbic acid, and 1 mM dibutyl-cyclic AMP.

### Transcriptome Analyses

Description of sample preparation and Illumina-HT12 microarray analysis is outlined in Supplemental Experimental Procedures. A log_2_ detection threshold of 3.2 was applied to the quantile normalized log_2_ data, retaining probes expressing above threshold in at least 50% of samples. After filtering, 17,259 probes were considered for a two-factor differential expression analysis (by phenotype and day) using Limma (Ritchie et al., 2015). Due to earlier removal of two outlying samples, a mixed linear model was applied, treating variance within cell line clones as random effects. Multiple pairwise contrasts between phenotypes and days of interest were fitted to the linear model and top tables of significant differential expression probes by empirical Bayes moderated t test were produced. Results were FDR p < 0.01 adjusted (Benjamini-Hochberg). A fold change threshold of 1.5 was used in heatmaps and volcano plots. Probes were annotated to genes using the hg19 Ensembl human genome assembly.

### Immunofluorescence and Flow Cytometry

Staining with antigen-specific and fluorescent antibodies was performed essentially as described (Ovchinnikov et al., 2015a) with minor modifications (see Supplemental Experimental Procedures). Antibodies used in this study are listed in Supplemental Experimental Procedures.

### Western Blotting

Western blotting of the Laemmli/RIPA-based protein lysates supplemented with protease and phosphatase inhibitors (Roche/Life Sciences, USA) was performed using the iBlot transfer system (Life Technologies, USA) and detected using Clarity ECL (Bio-Rad) according to the manufacturer's recommendations (see details in Supplemental Experimental Procedures).

### Measuring Soluble Products of APP Proteolysis

To measure production of different forms of Aβ in the medium of neurogenic cultures of various genotypes, we utilized ELISA kits specifically aimed at detection of the two most relevant and dominant Aβ species: Aβ40 (KHB3482) and Aβ42 (KHB3441) (Thermo Fisher Scientific/Life Technologies). Medium was used in 1:20 dilution in ELISA buffer for direct measurement of absolute peptide concentration using the provided standard curves. Peptide concentrations were normalized to βIII-tubulin as measured by western blotting from cells that produced secreted APP products.

## Author Contributions

D.A.O. and E.J.W. conceived the project and designed experiments; D.A.O. performed all wet-lab experiments and data analyses; C.A.W. contributed to experimental design; C.A.W., I.V., and O.K. contributed to bioinformatics analyses; D.A.O. and E.J.W. wrote the manuscript; all authors edited the manuscript.

## Accession Numbers

The accession number for the dataset reported in this paper is GEO: GSE100680.

## Supplemental Information

Document S1. Supplemental Experimental Procedures, Figures S1–S4, and Tables S1–S4

Document S2. Article plus Supplemental Information
