# Supplementary material for: The Impact of APP on Alzheimer-like Pathogenesis and Gene Expression in Down Syndrome iPSC-Derived Neurons
Source: Stem Cell Reports. 2018 May 31;11(1):32–42. doi: 10.1016/j.stemcr.2018.05.004 (PMC6066957; doi:10.1016/j.stemcr.2018.05.004)
Supplement: Document S1. Supplemental Experimental Procedures, Figures S1–S4, and Tables S1–S4 [file mmc1.pdf]

Stem Cell Reports, Volume *11*

Supplemental Information

The Impact of APP on Alzheimer-like Pathogenesis and Gene Expression in Down Syndrome iPSC-Derived Neurons

Dmitry A. Ovchinnikov, Othmar Korn, Isaac Virshup, Christine A. Wells, and Ernst J. Wolvetang

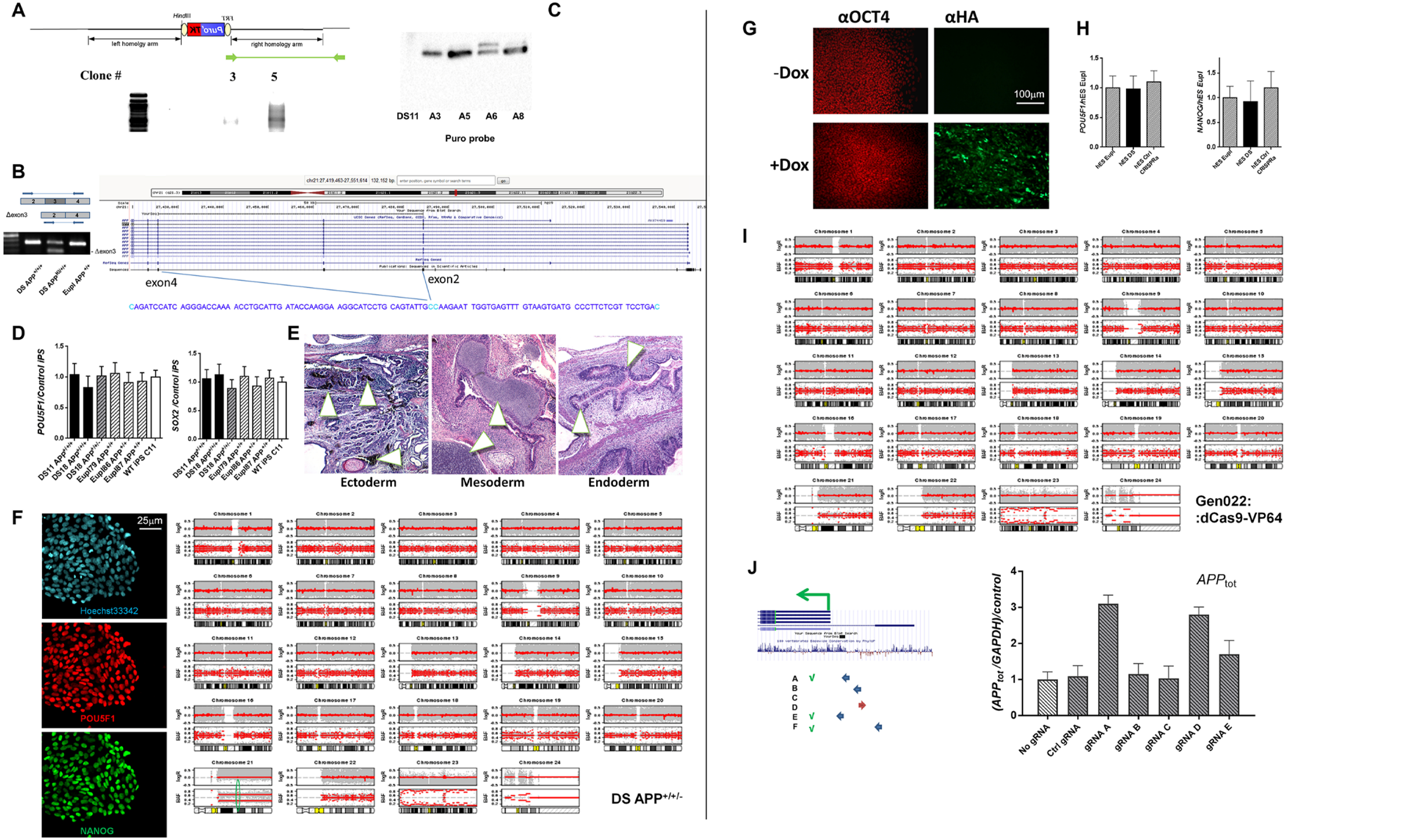

Supplemental Figure S1. (related to Figure 1) Validation of the clonal CRISPR-edited DS iPSC and CRISPRa hESC lines. A. Targeted allele-specific right homology arm-spanning PCR, with primers residing outside of right homology arm and within the selection cassette. **B.** Sequence and alignment of the amplicon of mRNA spliced in APP-targeted clone across the exon 3. **C.** Southern blot of the parental DS iPSC line and Puromycin-resistant clones using a Puromycin cassette-specific probe. **D.** qPCR validation of pluripotency marker expression in the isogenic iPSC cohort. **E.** H&E staining of the teratoma sections illustrating differentiation into the 3 germ layers (ectoderm, mesoderm, endoderm). **F.** Immunofluorescent detection of pluripotency markers POU5F1/OCT4 and NANOG in the DS APP$^{+/+/-}$ iPSC clone. Right panel; SNP analysis gDNA DS APP$^{+/+/-}$. **G.** Immunofluorescence staining of the Gen22::TRE-dCas9-VP64 line with anti-OCT3/4 and anti-HA (to detect HA-dCas9-VP64), in the absence or presence of doxycycline. **H.** qPCR analysis of pluripotency-associated gene expression in Gen21, Gen22, and Gen22::TRE-dCas9-VP64 lines **I.** SNP analysis of Gen22::TRE-dCas9-VP64 CRISPRa line. **J.** Validation of the 5 gRNAs targeting proximal to the main promoter region of the *APP* gene for their ability to mediate activation using a CRISPRa approach in 293FT cells. Values are shown as mean + SEM. N=3, n=2 for **D, H.**

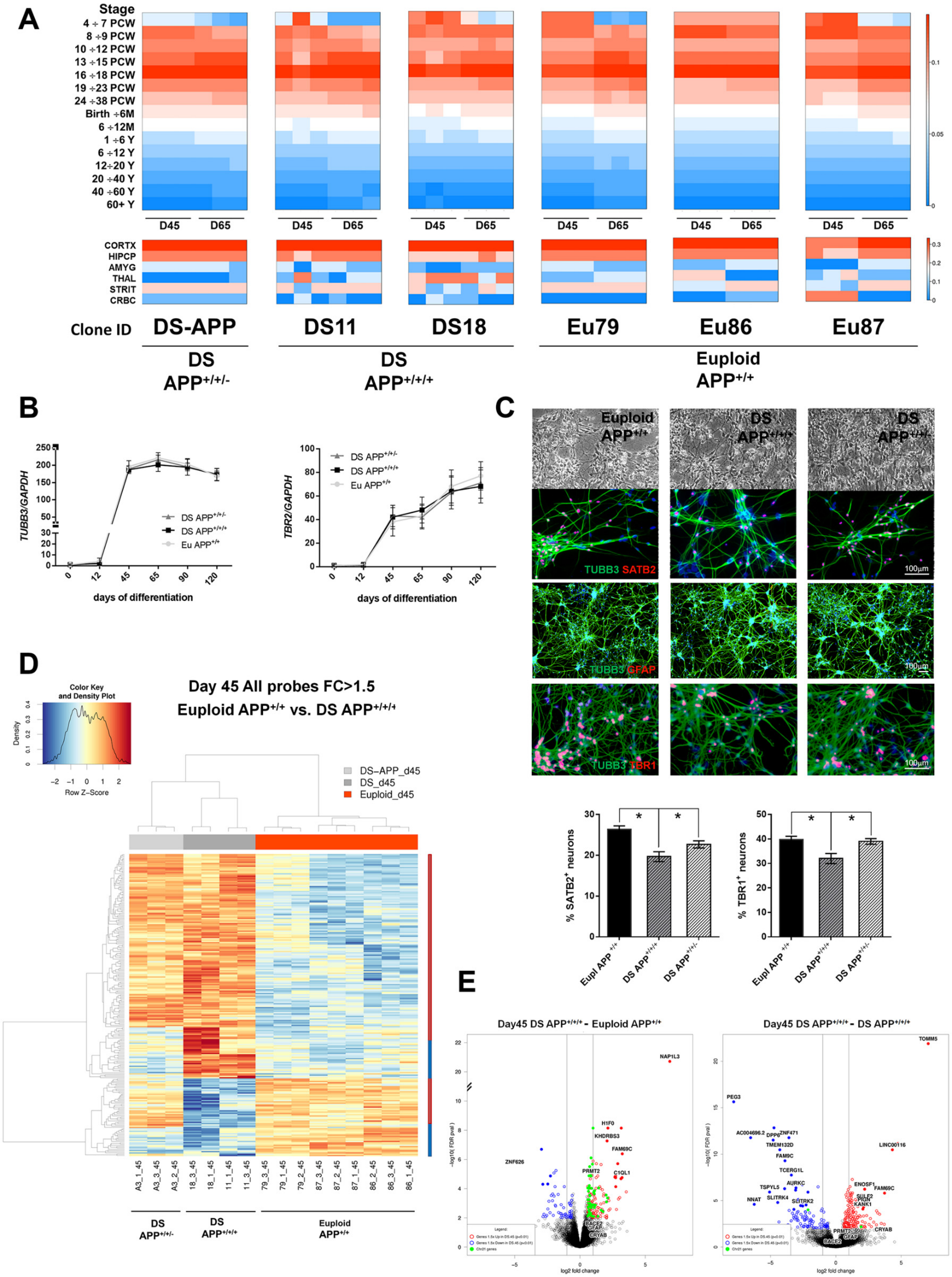
A
Stage
4 ÷ 7 PCW
8 ÷9 PCW
10 ÷12 PCW
13 ÷15 PCW
16 ÷18 PCW
19 ÷23 PCW
24 ÷38 PCW
Birth ÷6M
6 ÷12M
1 ÷6 Y
6 ÷12 Y
12÷20 Y
20 ÷40 Y
40 ÷60 Y
60+ Y
D45
D65
0.1
0.05
0
CORTX
HIPCP
AMYG
THAL
STRIT
CRBC
0.3
0.2
0.1
Clone ID
DS-APP
DS11
DS18
Eu79
Eu86
Eu87
DS APP+/+/-
DS APP+/+/+
Euploid APP+/+
B
TUBB3/GAPDH
DS APP+/+/-
DS APP+/+/+
Eu APP+/+
days of differentiation
TBR2/GAPDH
C
Euploid APP+/+
DS APP+/+/+
DS APP+/+/-
TUBB3 SATB2
TUBB3 GFAP
TUBB3 TBR1
100µm
% SATB2+ neurons
% TBR1+ neurons
Eupl APP +/+
DS APP+/+/+
DS APP+/+/-
D
Color Key and Density Plot
Density
Row Z-Score
Day 45 All probes FC>1.5
Euploid APP+/+ vs. DS APP+/+/+
DS–APP_d45
DS_d45
Euploid_d45
A3_1_45
A3_3_45
A3_2_45
18_3_45
18_1_45
11_1_45
11_3_45
79_3_45
79_1_45
79_2_45
87_3_45
87_2_45
87_1_45
86_2_45
86_3_45
86_1_45
DS APP+/+/-
DS APP+/+/+
Euploid APP+/+
E
Day45 DS APP+/+/+ - Euploid APP+/+
Day45 DS APP+/+/+ - DS APP+/+/+
NAP1L3
H1F0
KHDRBS3
FAM69C
ZNF626
PRMT2
C1QL1
BACE2
GFAP
CRYAB
TOMM5
PEG3
AC004696.2
DPP6
ZNF471
TMEM132D
FAM9C
LINC00116
TCERG1L
AURKC
TSPYL5
SLITRK4
SLITRK2
NNAT
ENOSF1
FAM69C
SULF2
PRDM
KANK1
PRMT2
GFAP
CRYAB
BACE2
-log10(FDR pval)
log2 fold change
Legend:
Genes 1.5x Up in DS.45 (p<0.01)
Genes 1.5x Down in DS.45 (p<0.01)
Chr21 genes

Supplemental Figure S2 (related to Figure 2). Transcriptomic profiling and characterization of the cortical neurogenic cultures A. Temporal and spatial identity assignment of the day 65 neuronal differentiation samples (5 clones: APP copy number-normalized DS11, 2 Hsa21-trisomic DS and 3 euploid clones) using RNA expression data from Illumina HT12 microarray and an online resource CoNTExT (https://context.semel.ucla.edu/). Scale bars reflecting predicted probabilities of temporal (top) or spatial (bottom) class memberships are shown on the right. **B.** Time course qPCR analysis of pan-neuronal marker *TUBB3* and intermediate neuronal progenitor marker *TBR2* during the cortical neuronal differentiation of the isogenic iPSC cohort **C.** Representative phase-contrast (top row) and epifluorescence (rows 2-4) images of neuronal cultures of all 3 genotypes at day 90 (GFAP staining in 3rd row at day 55) indicates comparable abundance of cortical layer markers SATB2 and TBR1 (quantification relative to TUBB3^{+} cells shown in **C'**). **D.** Heatmap based on hierarchical clustering of all significantly differentially-expressed probes (|FC|>1.5, P_{adj} <0.01) at day 45. **E.** Volcano plots for probes differentially expressed in DS vs euploid (left) and DS vs. APP copy number-normalized DS neuronal cultures (right) at day 45. Hsa21 genes shown in bright-green, genes down- or up-regulated >1.5 fold shown in blue and red, respectively. Negative decimal logarithm of the false discovery rate probability plotted on Y axis, log_2 of the fold change plotted on X with thin line at 0.5/2 FC values. Values are shown as mean ± SD (**B, C'**).

`

Supplemental Figure S3 (related to Figures 3, 4). A. Western blot showing upregulation of APP levels is retained in day 90 DS neurones, and fully offset by normalisation of the APP copy number. Quantification relative to beta-Actin. **B.** Quantification of Aβ42 and Aβ42/40 ratios in medium of day 90 DS and sibling euploid hESC-derived neurons (as measured by peptide-specific specific ELISA). **C.** Quantification of Aβ40 petide levels in day 90 culture medium from DS, DS copynumber normalised and euploid neurons. **D.** Effects of APP level modulation on *BACE2* (3 left panels) and *BACE1* transcript levels (qPCR) at indicated timepoints. **E.** Modulation of the Aβ42/40 ratio by the BACE inhibitor Verubicestat (Verub., used at 1 nM in DMSO) based on measurements from the medium of 90 day-old neurons of all 3 DS patient-derived genotypes described in this study. **F.** Effect of Verubicestat and the γ-secretase inhibitor DAPT on absolute levels of Aβ42 peptide secreted by day 90 DS neurons. **G**. Example of a western blot for pyroglutamate(pE3)-Ab in isogenic DS neurons quantified in Figure 3F. **H**. Western blot and its quantification showing pE3-pyroglutamate levels relative to TUBB3 in APP-overexpressing day 90 Gen22::TRE-dCas9-VP64 derived neurons bearing the indicated gRNAs. **I.** An example of TUBB3/Hoechst33342 - stained neuronal cultures at day 90 (top) used for automated neurite length calculation (see Supplemental information for details). **J.** An isogenic cohort of 2 DS trisomic clones, APP copy number-normalized clone and 3 euploid clones (at day 65 of neuronal differentiation); **K.** Gen22::TRE-dCas9-VP64 derived neurons (day 90) bearing the indicated APP promoter targeting gRNAs. Values shown are means ±SEM, N=3, n=2. * p<0.05, ** p<0.01, *** p<0.001, # ns. Mean values and SEM shown on graphs.

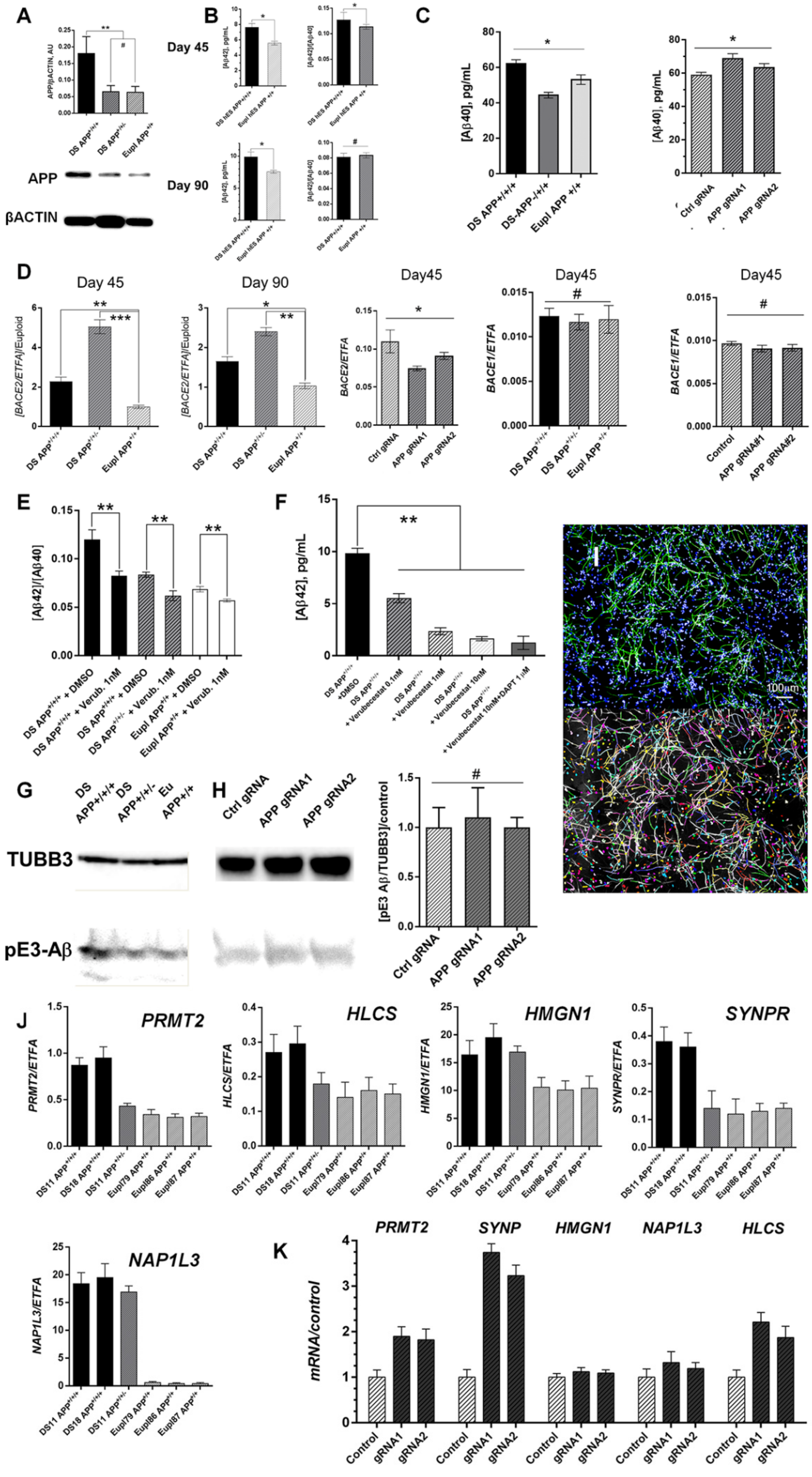

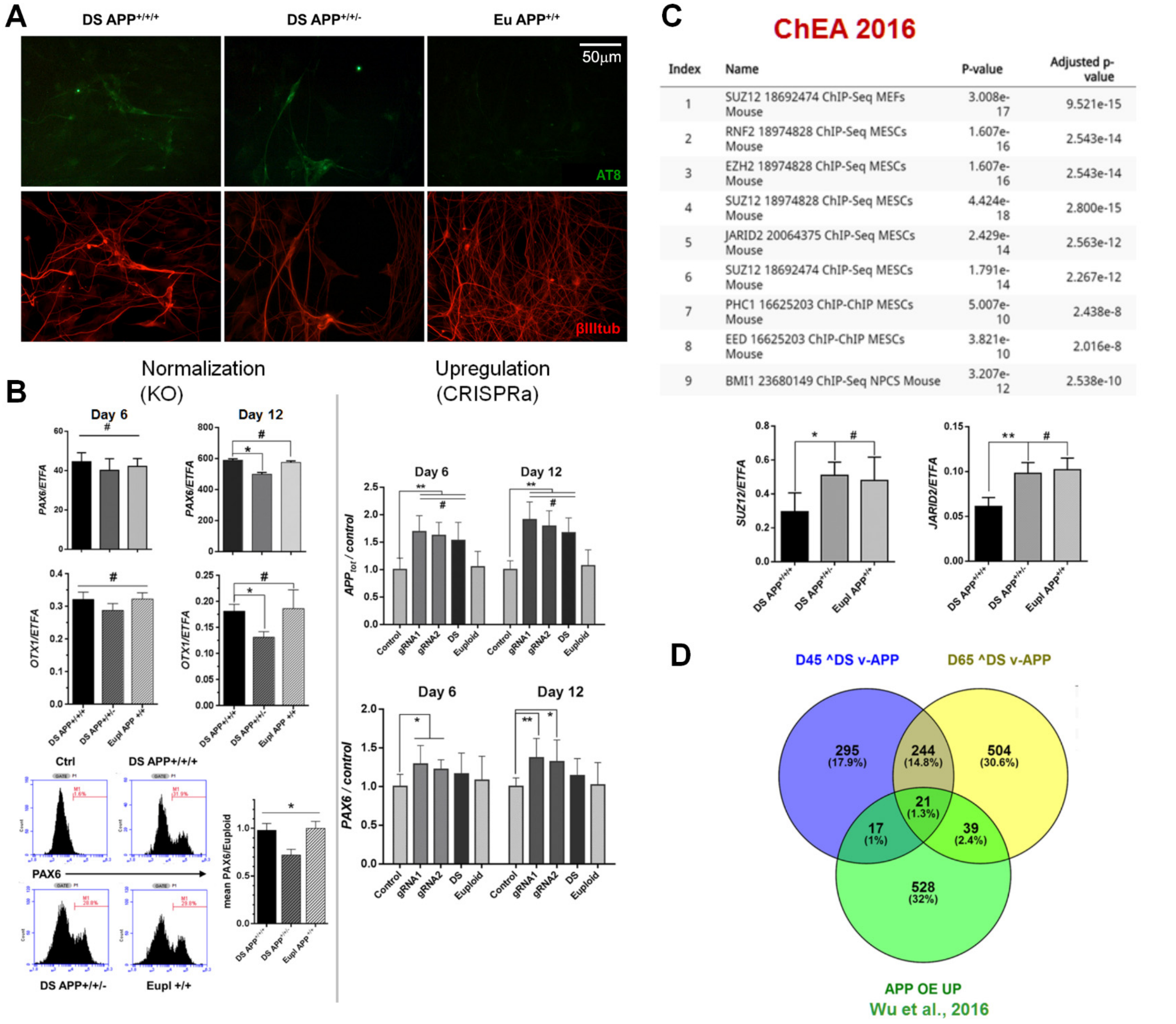

Index	Name	P-value	Adjusted p-value
1	SUZ12 18692474 ChIP-Seq MEFs Mouse	3.008e-17	9.521e-15
2	RNF2 18974828 ChIP-Seq MESCs Mouse	1.607e-16	2.543e-14
3	EZH2 18974828 ChIP-Seq MESCs Mouse	1.607e-16	2.543e-14
4	SUZ12 18974828 ChIP-Seq MESCs Mouse	4.424e-18	2.800e-15
5	JARID2 20064375 ChIP-Seq MESCs Mouse	2.429e-14	2.563e-12
6	SUZ12 18692474 ChIP-Seq MESCs Mouse	1.791e-14	2.267e-12
7	PHC1 16625203 ChIP-ChIP MESCs Mouse	5.007e-10	2.438e-8
8	EED 16625203 ChIP-ChIP MESCs Mouse	3.821e-10	2.016e-8
9	BMI1 23680149 ChIP-Seq NPCS Mouse	3.207e-12	2.538e-10

Supplemental Figure S4 (Related to Figures 4, 2). Interpretation and comparison of mRNA expression profiling results from the isogenic cohort of DS (Hsa21-trisomic), euploid and APP copy number-normalized DS neurons. A. Detection of hyperphosphorylated tau with AT8 anti-phospho-Tau antibody (Day 120 neuronal culture). **B.** Venn diagram showing overlap of DEG lists of transcripts upregulated in DS vs euploid and downregulated in DS-APP vs DS at days 45 (blue circle) and 65 (yellow circle) with APP overexpression-driven genes (green) from Wu et al. **C. S**ignificance of an overlap between 265 (244+21) APP copy number dependent differentially expressed genes with ChIP Enrichment Analysis (ChEA 2016), qPCR validation of APP copy number-dependent expression of the PRC2 components, *SUZ12* and *JARID2,* in day 65 neuronal cultures **D.** Venn diagrams built using a free online resource http://bioinfogp.cnb.csic.es/tools/venny/. * p<0.05, ** p<0.01, # ns. N=3, n=2 for qPCR Values shown are means±SEM.

Table S1 (Related to Figure 2): Analysis of the pathway alignment of APP-dependent or dynamically-regulated gene sets (using ToppGene online tool); validation of the APP synexpression groups using the Enrichr integrated gene list analysis online tool.

APP-regulated genes at day 65 (shown in Venn diagram in Figure 2B)

DAPL1, LOC404266, CRYAB, ALDH1A3, SCRG1, GFAP, RBP1, CFI, C18ORF51, MMP7, DACT2, HSD17B2, C1QL1, SYNPR, RDH10, DCT, HOPX, HOXB6, SIX6, NRIP3, A2M, SPP1, LEFTY2, METRN, PMP2, SCUBE2, HS.204481, CA14, IGFBP5, KAL1, CHSY3, C21ORF62, MARCH3, LMO2, RAB31, SMOC1, TAGLN2, PRSS23, LOC100133609, HS.569104, NFIX, SYT4, TRAF3IP2, F3, CPNE4, SLC44A3, TNC, DKK3, PPP1R1B, RBM20, RELN, BHLHE22, BAALC, NECAB2, CPS1, RHPN2, EBF3, SERPINB6, CRYM, RASD1, ATP1B2, MGP, SULF2, LPAR1, PCDH17, GLIS3, HTRA1, KANK1, NKAIN4, GPC3, HS.531457, NHLH2, RGS20, PODXL, LYN

**Gene expression signature/ID**	Gene overlap, UP @D45	Gene overlap, UP @D65
**Down syndrome (umls:C0013080)**	IL10RB, C21orf33, PIGP, DONSON, PDE9A, GART, WRB, DSCR3, PFKL, APP, TTC3, USP16, PDXK, IFNAR1, IFNGR2, SYNJ1, PSMG1, N6AMT1, CYYR1, PKNOX1, CSTB, PRMT2, CRYZL1, TMEM50B, SON	MRPL39, IL10RB, U2AF1, C21orf33, ATP5O, WRB, CSTB, HLCS, PRMT2, CRYZL1, BACE2, TMEM50B, GABPA, MX1, IFNAR2
**Alzheimer's Disease (umls:C0002395)**	PAG1, RELN, MAOA, SERPINF1, GART, CPLX1, GSTO2, EOMES, C1QL1, CDK5R1, SYNJ1, KCND2, NFIX, SYN1, SYN2, BACE2, HS3ST1, HOMER2, CA2, CAMK2A, NTS, KHDRBS2, GRIN1, APP, SLC1A2, IGFBP5, SCG3, TSPAN7, ELAVL4, SNAP25, CTNNA2, SNCA, MYO16	

Dynamically-regulated pathways D65-D45	Euploid (3 clones) D65-D45	
trans-synaptic signalling (GO:0099537)	DLGAP2, PDYN, TMOD2, MGLL, MEF2C, NRXN1, SYT13, PMCH, HTR2C, PPFIA4, PPFIA2, ICA1, BCAN, APOE, GABBR2, SLC1A1, SLC1A2, PRKCE, SNCB, ATP2B2, GABRA2, GABRG2, GLRB, TSPO, SLC17A7, DLG2, GRIN3B, CA2, HPCA, CAMK2A, CAMK2B, NTSR1, PCDHB4, GRIA2, GRIA3, GRIN1, SYT7, CCKBR, ZNF225, SEZ6, EXOC4	
, Neuronal system (#1268763)		DLGAP2, GNG8, MAOA, NRXN1, PPFIA4, PPFIA2, GABBR2, SLC1A1, SLC1A2, PRKACB, PRKCB, GABRA2, GABRG2, KCNA5, KCNF1, KCNJ4, KCNK1, SLC17A7, DLG2, CAMK2A, CAMK2B, GRIA2, GRIA3, GRIN1, SYT7, KCNH3

Table S2 (Related to Figure 2): Ingenuity (Qiagen) prediction of the pathways most-affected by the Hsa21 trisomy from DS vs Euploid comparison (D45)

Categories	Diseases or Functions Annotation	p-Value	Predicted Activation State	Activation z-score	№ of Molecules	Molecules
Neurological Disease	**neurological signs**	8.7E-08	⌂	2.184	43	ADCY1,APP,BHLHE22,CA2,CAMK2A,CAMK2B,CDK5R1,CHRM3,CNR1,CRYAB,CTGF,CX3CL1,DAAM2,DIO2,DIRAS2,DKK3,DMRT3,DONSON,ELAVL2,GPRC5C,GRIA2,GRIN1,GYPC,HSPA1A/HSPA1B,IER3,LMCD1,MAL2,NDRG1,NTF3,PCDH7,PLCB1,PPP1R1B,SALL4,SCN3B,SLC1A2,SMAD6,SNAP25,SNCA,SRPX,SYNJ1,SYNPR,TTR,VAMP2,ADCY1,APP,ATP1A2,BCHE,CA14,CA2,CA4,CCL2,CDK5R1,CHRM3,CNR1,CRH,GABBR2,
Neurological Disease	**cognitive impairment**	3.25E-06	⌂	2.173	29	GRIN1,MAF,MAOA,NTF3,PLAT,PMM2,PTGER4,SCN3B,SLC1A2,SNAP25,SNCA,SYNJ1,TSPAN7,TTR,TUBB2A,VAMP2
Neurological Disease, Organismal Injury and Abnormalities	**abnormality of cerebral cortex**	2.05E-09	⌂	2.05	25	ADCY1,APP,ATF3,BRINP1,CDK5R1,CNR1,CTNNA2,CTSV,CX3CL1,GAP43,GPR37,GRIA2,GRIN1,HSD17B2,NFIX,OTX1,OTX2,PLAT,POU3F2,SLC1A2,SNAP25,SNCA,SPP1,SYN1,VAMP2

Table S3 (Related to Figure 2): Analysis of the pathways influenced by APP copy number in DS neurons using the Reactome and KEGG DEG list pathway alignment-analysing open access bioinformatic tools

APP-dependent upregulated in DS in day 45 neurons

ID	Description	GeneRatio	BgRatio	pvalue	p.adjust	qvalue	GeneID
72163	mRNA Splicing - Major Pathway	25/603	115/6749	2.05E-05	0.008390092	0.007225009	HNRNPA1/HNRNPK/SF3A3/HNRNPA3/U2AF1/MAGOH/SF3B6/SNRPB/HSPA8/CPSF1/CSTF3/DHX9/SRSF2/SNU13/PHF5A/ALYREF/SNRPA1/SNRPA/HNRNPUL1/HNRNPM/HNRNPR/HNRNPD/POLR2C/PRPF19/PABPN1
72172	mRNA Splicing	25/603	115/6749	2.05E-05	0.008390092	0.007225009	HNRNPA1/HNRNPK/SF3A3/HNRNPA3/U2AF1/MAGOH/SF3B6/SNRPB/HSPA8/CPSF1/CSTF3/DHX9/SRSF2/SNU13/PHF5A/ALYREF/SNRPA1/SNRPA/HNRNPUL1/HNRNPM/HNRNPR/HNRNPD/POLR2C/PRPF19/PABPN1
2262752	Cellular responses to stress	43/603	256/6749	3.10E-05	0.008390092	0.007225009	CAT/HDAC6/NUP85/H3F3B/MAPKAPK3/RING1/UBE2D3/FKBP4/YWHAE/TXNRD2/VCP/PRDX5/HSPA8/RPA1/TNRC6B/IGFBP7/CBX4/HSPA1B/MAPK3/NUP107/TERF2/UBA52/MAPK8/UBE2D2/CAMK2A/UBB/CAMK2B/SOD1/PRDX3/GPX8/SEH1L/ID1
72203	Processing of Capped Intron-Containing Pre-mRNA	25/603	119/6749	3.79E-05	0.008390092	0.007225009	HNRNPA1/HNRNPK/SF3A3/HNRNPA3/U2AF1/MAGOH/SF3B6/SNRPB/HSPA8/CPSF1/CSTF3/DHX9/SRSF2/SNU13/PHF5A/ALYREF/SNRPA1/SNRPA/HNRNPUL1/HNRNPM/HNRNPR/HNRNPD/POLR2C/PRPF19/PABPN1
3560783	Defective B4GALT7 causes EDS, progeroid type	8/603	19/6749	0.000120295	0.017743474	0.015279543	CSPG5/BCAN/GPC6/SDC2/BGN/NCAN/GPC4/SDC1
3560801	Defective B3GAT3 causes JDSSDHD	8/603	19/6749	0.000120295	0.017743474	0.015279543	CSPG5/BCAN/GPC6/SDC2/BGN/NCAN/GPC4/SDC1
1971475	A tetrasaccharide linker sequence is required for GAG synthesis	9/603	25/6749	0.000188604	0.023844918	0.020533715	CSPG5/BCAN/GPC6/SDC2/BGN/NCAN/GPC4/SDC1/GXYLT1

APP-dependent upregulated in DS day 65 neurons

ID	Description	GeneRatio	BgRatio	pvalue	p.adjust	qvalue	GeneID
422475	Axon guidance	58/753	292/6749	6.15E-06	0.005696398	0.005484652	EPHB4/CRMP1/ARHGEF7/ST8SIA2/LAMB1/MYH10/CDC42/LAMA1/VASP/DNM1/AP2B1/YES1/DOCK1/KRAS/ABLIM1/SDC2/CDK5R1/TIAM1/GRIN1/DPYSL2/NCAM1/ERBB2/LAMC1/
112315	Transmission across Chemical Synapses	42/753	194/6749	1.43E-05	0.006623393	0.00637719	GNG5/PPFIA2/STXBP1/SLC6A1/CAMK2B/GABRB3/GABRA2/GABRA5/GABRG2/GNG2/PRKCB/SLC1A1/GABRA3/AP2B1/GNGT1/LIN7B/CPLX1/GNB1/CALM3/GRIN1/SNAP25/GAD1/VA
1566948	Elastic fibre formation	14/753	39/6749	4.27E-05	0.013187281	0.012697086	ITGB5/MFAP4/BMP7/FBN2/LTBP3/BMP2/FBLN5/FBLN1/MFAP2/LOXL1/ITGB1/ELN/ITGA5/FBLN2
888590	GABA synthesis, release, reuptake and degradation	9/753	19/6749	8.37E-05	0.019381838	0.018661381	STXBP1/SLC6A1/CPLX1/SNAP25/GAD1/VAMP2/STX1A/GAD2/SLC32A1
977441	GABA A receptor activation	7/753	13/6749	0.00019606	0.025658115	0.024704357	GABRB3/GABRA2/GABRA5/GABRG2/GABRA3/GABRB1/GABRA1
1266738	Developmental Biology	73/753	438/6749	0.000213329	0.025658115	0.024704357	SMAD4/MED30/EPHB4/CRMP1/ARHGEF7/MED25/ST8SIA2/LAMB1/MYH10/CDC42/LAMA1/MED16/VASP/DNM1/AP2B1/YES1/DOCK1/KRAS/ABLIM1/SDC2/CDK5R1/TIAM1/KLF4/I
977443	GABA receptor activation	16/753	55/6749	0.000220293	0.025658115	0.024704357	GNG5/GABRB3/GABRA2/GABRA5/GABRG2/GNG2/GABRA3/GNGT1/GNB1/GABBR2/ADCY1/GNG12/KCNJ2/GABRB1/GABRA1/GABBR1
2129379	Molecules associated with elastic fibres	12/753	35/6749	0.000249015	0.025658115	0.024704357	ITGB5/MFAP4/BMP7/FBN2/LTBP3/BMP2/FBLN5/FBLN1/MFAP2/ITGB1/ELN/FBLN2
112310	Neurotransmitter Release Cycle	14/753	45/6749	0.000249377	0.025658115	0.024704357	PPFIA2/STXBP1/SLC6A1/SLC1A1/LIN7B/CPLX1/SNAP25/GAD1/VAMP2/SLC1A3/STX1A/MAOA/GAD2/SLC32A1

Table S4 (related to Figure S4): Gene memberships of the overlaps in APP-regulated gene sets identified using Venn diagrams depicted in Figure S4D

**244 genes Upregulated in DS vs Euploid and DOWNregulated in DS-APP vs DS**<br>**at both D45 and D65**	TOMM5, HOXB8, CRYAB, HOXB5, NLRP2, LOC205251, SULF2, LMO2, DAPL1, NFIX, ALDH1A3, CFI, PODXL, SYNPR, A2M, HOXA5, LOC404266, HS.19193, C1QL1, RAB31, RAX, ATOH8, BHLHE22, HOXC4, PRSS23, POU4F2, ITGB5, CYP1B1, HOXC6, PRPH, DACT2, HOPX, CHX10, ALPK2, HS.71947, ENOSF1, TNC, CLDN1, SIX6, COLEC12, EBF3, SERPINF1, C18ORF51, CPS1, WNT7B, GJA1, TXNL1, CXXC5, TYMS, CDKN1A, HOXB7, MALT1, TGIF1, ZNF680, TPBG, GLIS3, FABP7, ZNF521, KAL1, DUSP6, SMOC1, PARD6G, CYB5A, PIGN, CPNE4, KANK1, C1QTNF5, PPP4R1, ME2, MDK, RSPO3, SPARCL1, HOXB6, ADAMTS1, TUBB6, RDH10, NDUFV2, DKK3, ATP1B2, HS3ST3A1, ABLIM1, C3ORF58, PLS3, BCL2, RBMS1, NME5, CA14, ATOH7, RBM20, LOC643911, CHSY3, HOXC8, SERPINB6, PRODH, FVT1, NDC80, RASD1, DTNA, RELN, HS.531457, ZIC3, PLCE1, PPP1R1B, HS.552087, SPP1, AFG3L2, C12ORF31, PSMG2, IMPA2, MMP7, LOC100130502, LPAR1, PLTP, CXCR4, MEX3C, HOMER2, LOC285943, LOXL1, GDF10, C4ORF31, KLHDC8B, DCT, ZNF503, MRLC2, NHLH2, MYL12A, MARCH3, CDH6, EFCBP1, HOXB3, HSD17B2, DCN, KCNJ13, TPPP3, OTX2, FLNC, MAPK4, CCND1, CHMP1B, C18ORF1, C21ORF34, RHPN2, TRPM3, FAR2, SULF1, TRAF3IP2, CASP6, FHL2, FAM167A, FBN2, HS.553310, SERPING1, SYT4, LPIN2, ITGA2, TNFRSF11B, HS.574278, SEC11C, VWC2, PQLC1, ACOX2, HS.582985, ZNF248, LOC100133609, F3, GRM8, C1ORF24, RARB, HOXA2, C6ORF165, PDLIM1, LOC730254, PON2, C8ORF47, COL18A1, ZNF266, SLC39A4, HS.278285, CLSTN2, HS.569104, SCUBE2, TAGLN2, NRIP3, KIAA1468, C8ORF13, HS.552008, PLEKHA4, SEH1L, C21ORF62, SOCS3, NECAB2, SLC44A3, CHST3, COL4A1, HS.204481, ARL4C, FNDC5, ZNF532, CD44, TMEM132B, ID1, STX8, OSBPL1A, BAALC, SH3D19, RHOU, LAPTM4B, LOC154860, IL13RA1, SH3BP4, HS.143937, FEZ2, C18ORF22, EMP1, NPPB, C18ORF10, SCRG1, TGFBI, HEPH, ADAMTS3, IFITM2, GREM1, RNF182, CD9, KITLG, EPB41L3, ADD3, HOXB2, ABCA1, OTP, FBLN2, HS.61208, LOC401052, DYNLRB2, ANKRD12, GLIPR2, FOXJ1, FOLR1, SESN1, SDSL, AGTRAP, BMP7, LOC100134265, TSHZ,

**21 genes upregulated by APP in Wu et al. dataset and regulated by APP copy number at D45&D65**	RBP1, HTRA1, APCDD1, IGFBP5, ID3, BMP2, LAMA1, ARL4A, SILV, TWSG1, PMAIP1, SEMA6A, PTPRM, PCDH17, PMP22, VIM, GPC3, ACADVL, GPR98, LOC400657, MSRA

Alignment of the transcriptomic changes with gene sets regulated by components of the Polycomb Repressor Complex 2

Gene lists for ChEA 2016 analysis (Enrichr)	Test DEG list	P_{adj} value	Pathway overlap membership
SUZ12_ 18692474	See above	0.000000 0000000 1	**55/1135:** CLSTN2;FHL2;RAX;PTPRM;OTP;CDH6;SIX6;ADAMTS1;GRM8;CYP1B1;HOXA2;HOXA5;CHX10;SEMA6A;IGFBP5;LMO2;EBF3;C1QL1;GREM1;ALDH1A3;COL4A1;HOXB3;HOXB8;HOXB7;HOXB6;HOXB5;FBN2;HS3ST3A1;NFIX;HTRA1;CXCR4;SCUBE2;NHLH2;RELN;RASD1;PODXL;ATOH8;HOXC4;OTX2;FLNC;HOXC8;HOXC6;ABCA1;GDF10;DTNA;HOMER2;WNT7B;BMP7;POU4F2;BMP2;PPP1R1B;ID1;BCL2;ID3;TGFBI

EZH2 18974828	See above	0.000000000000025	58/1302: CLSTN2;RAX;PTPRM;OTP;PRSS23;SIX6;ADAMTS3;ADAMTS1;GRM8;HOXA2;CYP1B1;HOXA5;CHX10;SEMA6A;IGFBP5;LMO2;EBF3;C1QL1;GREM1;AFG3L2;ALDH1A3;COL4A1;HOXB3;HOXB2;HOXB8;HOXB7;HOXB6;HOXB5;FBN2;COLEC12;HS3ST3A1;NFIX;TSHZ1;GLIS3;HTRA1;CXCR4;PCDH17;SCUBE2;NHLH2;RELN;PODXL;ATOH8;GPC3;HOXC4;OTX2;FLNC;HOXC8;HOXC6;GDF10;HOMER2;WNT7B;BMP7;POU4F2;BMP2;PPP1R1B;BCL2;VWC2;ID3
SUZ12 18974828	As above	0.0000000000000028	75/1934: CLSTN2;FHL2;RAX;PTPRM;OTP;CLDN1;PRSS23;LOXL1;CDH6;SIX6;ADAMTS3;CCND1;ADAMTS1;SESN1;GRM8;HOXA2;CYP1B1;RSPO3;HOXA5;CHX10;TGIF1;MMP7;SEMA6A;IGFBP5;LMO2;EBF3;C1QL1;GREM1;ALDH1A3;RAB31;COL4A1;HOXB3;HOXB2;HOXB8;HOXB7;CD44;HOXB6;CHST3;HOXB5;FBN2;COLEC12;NFIX;HS3ST3A1;PON2;TSHZ1;HTRA1;GLIS3;CXCR4;PCDH17;SCUBE2;ATOH7;NHLH2;RELN;PODXL;ATOH8;GPC3;HOXC4;NRIP3;OTX2;FLNC;HOXC8;HOXC6;GDF10;HOMER2;WNT7B;TPBG;BMP7;POU4F2;BMP2;PPP1R1B;BCL2;VWC2;PMP22;ID3;VIM
JARID2_ 20064375	As above	0. 0000000000025	50/1117 CLSTN2;RAX;PTPRM;OTP;CDH6;SIX6;ADAMTS1;GRM8;CYP1B1;HOXA2;RSPO3;HOXA5;SEMA6A;IGFBP5;LMO2;EBF3;C1QL1;FNDC5;GREM1;COL4A1;HOXB3;HOXB2;HOXB8;HOXB7;HOXB6;HOXB5;FBN2;NECAB2;HS3ST3A1;NFIX;TSHZ1;GLIS3;HTRA1;CXCR4;PCDH17;SCUBE2;NHLH2;RELN;ATOH8;CHSY3;HOXC8;HOXC6;HOMER2;WNT7B;BMP7;POU4F2;BMP2;PPP1R1B;BCL2;VWC2
EED_ 16625203	As above	0.00000002	36/830: FBN2;COLEC12;NFIX;FHL2;RAX;CXCR4;OTP;LOXL1;NHLH2;SIX6;RELN;RASD1;ADAMTS1;PODXL;ATOH8;HOXA2;CYP1B1;HOXC4;PRODH;HOXA5;HOXC6;CHX10;GDF10;SEMA6A;IGFBP5;EBF3;BMP7;POU4F2;COL4A1;PMP22;ID3;HOXB3;HOXB2;HOXB8;HOXB7;HOXB6

Supplemental experimental procedures

Targeting the APP locus using CRISPR/Cas9-aided homologous recombination in hiPSCs

For gene targeting, $3x10^6$ pluripotent DS iPS cells were transfected with 2.5µg of targeting vector (Figure 1) together with 1 µg a pre-validated gRNA and Cas9 expressing plasmid (pX459, a gift from Feng Zhang, MIT) using CA-137 protocol in 4D Nucleofector (Lonza, Switzerland) according to manufacturer's instruction and primary cell transfection buffer kit PC3 (Lonza, Switzerland). Induction of *APP* overexpression was performed using aqueous doxycycline hyclate (Sigma-Aldrich, USA) at 1 µg/mL in culture medium unless indicated otherwise. Overexpression of APP by transfection was achieved using either 2 µg pCAX-APP695 plasmid (Addgene plasmid 30137) or co-transfection of 1 µg of SP-dCas9-VPR (Addgene 63798) and 0.5 µg each of validated (data not shown) gRNAs (in constitutive lentiviral vector Addgene 52628) targeting regions upstream of *APP* promoter into matured hiPSC-derived euploid neurons (from the isogenic 6-clone cohort) in a 24-well plate using Lonza neuronal transfection kit (Lonza, Switzerland).

To address the role of the supernumerary copy of the APP gene on development of AD neuropathology in DS we edited one APP allele in a previously characterised footprint-free DS-iPSC line with full trisomy of HSA21 - clone C11DS (Briggs et al., 2012). Exon 3 of the APP gene was replaced with a Puro-TK selection cassette using CRISPR/SpCas9-assisted homology-directed repair (DS $APP^{+/+/-}$ line, Figure 1A). Correct targeting was confirmed using PCRs specific to the correctly targeted allele (Figure 1B, Figure S1A) and Southern blotting (Figure 1C). While deletion of exon 3 still permits low-level splicing between exons 2 and 4 (Figure S1B, mRNA from neuronal cultures on day 65), splicing of the resulting transcript introduces a frameshift and should not lead to the synthesis of a functional APP protein. Indeed, the ~1.5-2-fold increase in APP protein expression observed in neuronally differentiated DS $APP^{+/+/+}$ iPSC was "normalised" to euploid levels in isogenic CRISPR-targeted $APP^{+/+/-}$ DS iPSC-derived neuronal cultures (Figures 1D and 1E), and this was maintained during prolonged neuronal differentiation (up to 90 days tested, Figure S3A). The clonal APP allele-targeted $APP^{+/+/-}$ DS iPSC line exhibits the expected expression of pluripotency markers (Figure S1D), the ability to form representatives of all three germ layers in teratoma assays (Figure S1E), and maintains a stable HSA21-trisomic karyotype without obvious deletions, duplications or insertions as assessed by SNP array analysis (Figure S1F).

Generation of stable hPSCs allowing for CRISPRa-driven upregulation of the APP gene.

To assess the effect of APP overexpression in a euploid (HSA21-disomic) background we modified a euploid human ES cell line Genea022 (Dumevska et al., 2016b) with lentiviral transgenes carrying a doxycycline-inducible artificial transactivator dSpCas9-VP64, permitting comparison with its sibling HSA21-trisomic DS hESC counterpart, Genea021 (Dumevska et al., 2016a). Transduction of the Genea022::TRE-dCas9-VP64 line with a puromycin-selectable lentivirus constitutively expressing one of the pre-validated gRNAs that direct the transcriptional activator upstream of the main *APP* promoter, allows for doxycycline-tunable overexpression of APP protein to levels comparable to DS (Figures 1F and 1G). Induction of the HA-tagged dCas9-VP64, and APP overexpression was confirmed by western blotting using antibodies listed below.

Generation of the cortical neurogenic cultures

Simultaneous inhibition of TGFbeta/Activin and BMP/GDF signalling pathways was used to generate neurogenic cultures (Briggs et al., 2013; Chambers et al., 2009). Briefly, SB431542 at 10µM and dorsomorphin at 5µM were used for inhibition of the abovementioned pathways, respectively, for the first 6 and 12 days of differentiation medium (KOSR gradually exchanged for N2B27 (neurobasal medium supplemented with 2% B27, 1% N2 and 1% GlutaMax , all from LifeTechnologies) in 25% incremental steps on days 4, 6, 8, 10). The neuroepithelial cell layer formed by day 6 was mechanically separated into evenly-sized pieces of ~500 cells/each and transferred in differentiation medium in ultra-low attachment plate for neurosphere formation. After day 12, neurospheres were plated on Matrigel-coated (1/50 dilution of original supplied stock in DMEM/F12 base medium) TC-treated plastic. Growing neurogenic cultures were passaged every ~10 days, and were plated on poly-L-ornithine/human laminin-coated TC plastic, glass coverslips or chamber slides (plastic or glass) 2-6 weeks before assays (unless specified otherwise). Further maturation was achieved by supplementing the differentiation medium with 20ng/mL BDNF, 10ng/mL GDNF, 500nM ascorbic acid and 1mM dibutyl-cyclic AMP.

Microarray analysis: sample and data preparation

Total RNA from triplicates of day 45 and 65 neuronal differentiation cultures[17,71] was prepared using direct lysis on the plate with TriZOL reagent (LifeTechnologies), followed by total RNA isolation using PureLink RNA kit

(Ambion/LifeTech) with on-column DNase treatment (as per manufacturer's instructions). After RNA integrity verification using BioAnalyzer (Agilent Technologies, USA), RIN=10 RNA samples were used for Illumina HT-12 microarray hybridization and an iSCAN analysis (performed by AGRF-Melbourne). The summarised probe profile and control probe files were read into the statistical programming language R (https://www.R-project.org/), and analysed using packages from the Bioconductor project(Huber et al., 2015). Quality was assessed using array QualityMetrics (Kauffmann et al., 2009), and two samples (DS11 and DS18 at day 45) were determined to be outliers and were removed from any subsequent analysis. The samples underwent *normexp* background correction using the negative control probes and quantile normalization using the negative and positive control probes, using the *normexp* function from the Limma package (Ritchie et al., 2015). Any probes not considered to be detected (using a detection p-value of less than 0.01), were removed, resulting in 25,378 probes and 34 samples. A linear model was fitted taking into account the group and the time point (results of this analysis were used for evaluation of spatiotemporal identity of neurogenic samples using CoNTExT and Enrichr analysis of consistently APP-dependent genes). For other analyses, the otherwise unaltered sample probe profile with remaining 34 samples was independently background-corrected and quantile normalized using R/Bioconductor package Lumi (Du et al., 2008) and further log2 transformed. This data was used in all subsequent analyses.

Western blotting (extended)

Cells were washed with PBS$^{(-/-)}$ twice and subsequently lysed with 2x Laemmli loading buffer (Sigma), containing 1 tablet of each complete protease inhibitor and PhosSTOP phosphatase inhibitor (Roche). DNA in samples were sheared with at least 27G syringe needles, heated at 65°C for 10 mins and resolved using sodium dodecyl sulphate polyacrylamide gel electrophoresis (SDS-PAGE). The iBlot® Dry Blotting System (Invitrogen) is used to transfer proteins onto a PVDF membrane using program P3 iBlot transfer device. Blots were rinsed in TBS and blocked in TBST (TBS + 0.05% Tween-20) containing either 3-5% bovine serum albumin (BSA, for western blotting, Cell Signalling Technologies) for phospho-protein detection or 5% skim dried milk (Diploma brand) at room temperature for ≥1 hour, probed with desired primary antibodies in blocking buffer at 4°C over-night. Membranes were then rinsed and washed thrice for 15 minutes each in TBST, probed with appropriate HRP-conjugated secondary antibodies in blocking buffer (typically 5% skim milk, normally at 1:1000 or higher dilutions) for at least one hour at room temperature, washed as before, rinsed in TBS and detected using Clarity ECL (BioRad) according to manufacturer's recommendations The images were analyzed using Image Lab (Bio-Rad) software.

Immunofluorescence and flow-cytometric (FACS) analyses

Staining with antigen-specific and fluorescent antibodies was performed essentially as described (Ovchinnikov et al., 2015; Ovchinnikov et al., 2014) with minor modifications. For staining with anti-phosphorylated proteins forms, PBS was substituted with TBS as a base buffer, and Triton-X100 was used at 0.25% for permeabilization in case of detection of a nuclear protein. All antibodies used in this study are listed below. For apoptosis measurement in neuronal cultures, 12 hours after hydrogen peroxide challenge we performed a TUNEL staining, a flow cytometry-optimized APO-BrdU™ TUNEL Assay Kit, with Alexa Fluor™ 488 Anti-BrdU (Invitrogen/ThermoFisherScientific) was used according to manufacturer's instructions (fractions of TUNEL^{+} nuclei used for calculations shown in Figure 4A-C). Observations were validated using an externalised phosphoserine-visualizing surface Annexin V staining (using Dead Cell Apoptosis Kit with Annexin V Alexa Fluor™ 488 & Propidium Iodide, Invitrogen/ThermoFisherScientific) 4 hours after the hydrogen peroxide challenge according to manufacturer's instructions.

Statistical analyses

N refers to a number of independently-repeated experiments, n- technical replicates (e.g. wells in q qPCR reaction or a staining/differentiation). Unless stated otherwise, the results represent an analysis of means of at least three independent experiments. Mean-of-the-ratios (MoR) comparison approach was always taken, i.e. values from repeated experiments were compared after internal normalization(s) (e.g. for each individual western blot protein form of interest was normalized against a housekeeping gene and/or unphosphorylated tau levels, *etc.*). Two-tailed t-test for used for pairwise comparison of the means for different genotypes, and one-way ANOVA for identification of the presence of an outlier sample in a set of three or more cohorts. Standard symbols were used to reflect p values obtained through the respective statistical analyses (using GraphPad Prizm software 6 or 7): #, n.s. - not statistically-significant (p>0.05), * - p<0.05, ** - p<0.01, *** - p<0.001, **** - p<0.0001.

Morphometric measurements of cells in neurogenic cultures

Neuronal neurite length parameters, were measured using Harmony 12.1 software. As a first step, individual cell assignment was carried out using nuclear detection ("Method M", typically diameter 8-10 µm, splitting coefficient and common threshold ~25%) on nuclear stain channel. TUBB3-positive cells/processes (Figure S3I) were used for calculation of the dendritic parameters using built-in "neurite length analysis" module (CSIRO) using parameter settings determined via training on representative samples for each multi-sample experiment. Example of numeric settings for main parameters: Smoothing width 3, Linear window 6, Contrast 3, Diameter 6 Gap closure distance 5, Gap closure quality 25, Debarb length 20, Body thickening 4, Tree length 15.

Analysis of the effects of APP copy number on expression of HSA21 genes

Interestingly, a significant fraction (~30%) of genes affected by normalization of the *APP* copy number in DS (HSA21-trisomic) map to chromosome 21 (volcano plot in Figure 2D, see GEO data set for chromosomal assignment details). Particularly at day 65, the supernumerary *APP* copy has a profound effect on mis-expression of genes from HSA21 with 25 of 329 HSA21 genes (HugoGeneNomenclatureCommittee site-HSA21 gene annotation) overexpressed in an APP-dependent manner, while at day 45 only 4 HSA21 genes were overexpressed in an APP-dependent manner (Figure 2C). ENRICHR analysis reveals a significant overlap with gene sets involved in abnormal nervous system and abnormal brain morphology but not with Alzheimer's disease, as would perhaps be anticipated given that APP is thought to be an important determinant of AD-like neuropathology in DS. Collectively, our data suggest that even a modest increase in APP levels in a trisomy 21 context has profound effects on the transcriptional makeup of DS neurons, and that the impact of APP on gene expression increases with neuronal maturation, and has a particularly pronounced effect on over-expression of other HSA21 genes through an as yet unidentified mechanism (See GEO dataset for more details).

Identification and evaluation of putative epigenetic mechanisms mediating genome-wide supernumerary *APP* copy impact in Hsa21-trisomic DS iPS-derived neurons

To identify other potential mechanisms responsible for the observed APP-dependent genome-wide transcriptome changes we compared the 265 genes that are overexpressed in DS neurons in an APP-dependent fashion at both days with existing ChIP datasets (Lachmann et al., 2010), available through Enrichr online analysis tool suite (Chen et al., 2013; Kuleshov et al., 2016). This identified, at the high confidence levels ($p<2E-8$, Figure S4C), enrichment for targets of components of the polycomb repressive complex 2 (PRC2), such as SUZ12, EZH2, JARID2 and EED. Significantly, *SUZ12* and *JARID2,* both obligatory PRC2 components, are repressed in DS neurons in an APP copy number-dependent manner (see Figure S4C and Supplemental Table S4 for overlap gene memberships). How APP is mechanistically involved in this remains unclear and likely complex since a number of other epigenetic modifier genes were found to be upregulated in an APP-dependent and independent fashion (Figure S3J) in DS neurons. These include *HLCS,* a HSA21 gene implicated in modulation of repression-associated chromatin complexes (Zempleni et al., 2011; Zempleni et al., 2014) and *HMGN1,* a protein that associates with chromatin-accessible domains and enhancers and displaces heterochromatic histone H1 (Martinez de Paz and Ausio, 2016) (Figure S3J). Overexpression of APP in CRISPRa day 90 euploid neurons is sufficient to upregulate *HLCS* but not *HMGN1* (Figure S3K).

Comparison of neuronal gene expression datasets with previously reported data

Transcriptome comparison of our footprint free isogenic cohort of DS IPSC derived neurons with previously reported (Weick et al., 2016) 30 day-old retrovirally generated DS iPSC-derived neurons reveals substantial overlap in overexpressed HSA21 genes at day 45 (65%), and at day 65 (59%) (data not shown). However, when all up-regulated genes are considered, the overlap drops to 25% on day 45, and 12% on day 65 (data not shown), suggesting that while the core HSA21-driven overexpression cohort is largely preserved, neuronal cultures of different ages display highly-dynamic misexpression pattern of genes residing on other chromosomes. The limited overlap amongst the identified gene sets may also be due to platform differences, our more stringent cut-off used, the maturity of the cultures, or method of iPSC generation (footprint-free vs retroviral iPS cells. Comparison of our APP-dependent gene sets with a data set kindly provided by Wu et al. (Wu et al., 2016), who overexpressed APP in HEK293 cells, also did not show significant overlap, suggesting these gene regulatory effects of APP are likely to be cell context-dependent (Figure S4B). DEG overlap memberships are listed in Supplemental Table S4 and could be retrieved from GEO and Wu et al. resources online).

Oligonucleotides used in this study

qPCR primer sets

ETFA	TGTTGATGCTGGCTTTGTTC	TGGATGGCTCCAGATATTCC
APP	GATGCGGAGGAGGATGAC	TCTGTGGCTTCTTCGTAGG
NANOG	GATAGATTTCAGAGACAGAAATACCTCAGC	AGGCCTTCTGCGTCACACC
APP_{tot}	CATGGTCCAGAGTGGAAGCCATGGTC	GAGAGACTGATTCATGCGCTC
APP_{695}	AGGAGGAAGAAGTGGCTGAGGTGGA	TCTCTCGGTGCTTGGCCTCAA
APP_{751}	GCCGAGCAATGATCTCCCGCTGGTA	TCTCTCGGTGCTTGGCCTCAA
APP_{770}	GTGTGCTCTGAACAAGCCGAGAC	GGATCTCGGGCAAGAGGTTC
ASCL1	TCTCATCCTACTCGTCGGACGA	CTGCTTCCAAAGTCCATTCGCAC
DCX	TCCAGCAGCCAGCTCTCTACC	TACAGGTCCTTGTGCTTCCG
BACE1	ACCAACCTTCGTTTGCCCAA	TCTCCTAGCCAGAAACCATCAG
BACE2	GGAGATGCTGATCGGGACC	AGTACGTGTCTATGTAGGAGTGC
PAX6	CAGCACCAGTGTCTACCAACCA	CAGATGTGAAGGAGGAAACCG
GAPDH	GAGTCAACGGATTTGGTCGT	TTGATTTTGGAGGGATCTCG
GLAST	TACCCTTCGCTCAAAGTCACC	CCGATCTGGGCTGAGTCTTTT
GLI1	CCCAGTACATGCTGGTGGTT	GCTTTACTGCAGCCCTCGT
HB9	CGCTCTCCTACTCGTACCCG	GGAGTTGAAGTCGGGCATCTTA
HES1	GTGCGAGGGCGTTAATACCG	TGATCTGGGTCATGCAGTTGG
HOXB4	GCAAAGAGCCCGTCGTCTACCG	TGTCAGGTAGCGGTTGTA
NCAM	ACCTGGAGGACTTCTACCCG	ACCATGTGCCCATCCAGAGTC
NKX2.2	AACGATGAGGAGGGCTCTGTG	TGTCGCTGCTGTCGTAGAAGG
OLIG2	CACGGCCTACTCAAGTCTCCG	ACTTGGCGTCGGAGGTGAGG
OTX1	CTACCCTGACATCTTCATGCGGG	GAGAGGACTTCTTCTTGGCTG
OTX2	AAAGAAGACATCTCCAGCTCG	GTCGGGACTGAGGTGCTAGAGG
SOX1	TCCTGGAGTATGGACTGTCCG	GAATGCAGGCTGAATTCGG
SOX2	CCACCTACAGCATGTCCTACTCG	GGGAGGAAGAGGTAACCACAGG
SOD1	CTCACTCTCAGGAGACCATTGC	CCACAAGCCAAACGACTTCCAG
DSCR1	AAACTTCAGCAACCCCTTCTC	CCACTTGTTTCCATCCCACT
DYRK1A DO1	TTCAGTGGTGCCAATGAGGT	TTCTTGCTTTTGGTGCTTGGT
DYRK1A DO2	GCACACTGGAGAACCTCTGTT	AGCAGGTGGAATACCCAGAACT
S100B	TCCCGGGATGCGCCTGATCA	CCCCCAGAGCTGGCTCGGAT
SYNP	GCATCGACATAGCGTTTGCC	GACGAGGAGTCACCAATCAATG
MMP7	GAGTGAGCTACAGTGGGAACA	CTATGACGCGGGAGTTTAACAT
JUN	AACAGGTGGCACAGCTTAAAC	CAAACTGCTGCGTTAGCATGAG
HMGN1	GCGAAGCCGAAAAAGGCAG	TCCGCAGGTAAGTCTTCTTTAG

NAP1L3	GCAGTTAGAAATCGTGTGCAAG	TGTAGGCTCGTATTCTGCATTGA
H1FO	ACCCTCCTCGACTTCCACAG	TGTAGAGCTTGATAGCTGCCA
HLCS	CCCGTTGAGCATTATCACCTC	GGCAGACGCAAACTTGACTG
PRMT2	ACATTCCGGCAAACCATGTG	GGATGACTTTATCCGTCAGGG

CRISPR/SpCas9 manipulation of the *APP* locus used in this study

gRNA APP3.2	CACCGACCTGCATTGATACCAAGGA	AAACTCCTTGGTATCATGCAGGTC
SNA PCR APP3 435bp	CTGGGTGACAGAGTGAGGAAA	ACCCACTGATGGTAATGCTGG
Ca APPgRNA1	CACCGCGCGAGCGGGCGCAGTTCCC	AAACGGGAACTGCGCCCGCTCGCGC
Ca APPgRNA2	CACCGCCCACAGGTGCACGCGCCCT	AAACAGGGCGCGTGCACCTGTGGGC

Primers used for *APP* gene level manipulation using CRISPR/Cas9 technology

		Forward primer	Reverse primer
**Homology arm amplification**	**Left: 702 bp**	aaaaaggtaccGAACTTGACTTCTGCAATGACTGGT	Aaaaa ggcgcgccGCCTGGTCTTGAGGGAAGAAAAAG
	**Right: 793 bp**	aaaaaagcggccgcAAGCATCTAACAAGCCTCCACTG	aaaaaaccatggGGGGCTGTAGTTAGTTATAGGAGC
**Screening PCR**	**Left: 729 bp**	GTCCACAATCTCCACAGAC	AGGCCTACCCGCTTCCATTGCTC
**Puromycin-targeting probe PCR**	**483 bp**	ACCGAGTACAAGCCCACGGTGC	TCGTAGAAGGGGAGGTTGCGGG

Antibodies used in this study

Antibody	Recommended dilutiuon	Catalog#	Source
**APP (C-term)**	WB 1/1000, 5% CST-BSA	CST.2452S	CST
**GAPDH (D16H11) XP®**	WB 1/2000 NFDM block (be default)	CST.5174S	CST
**DYRK1A (D30C10) Rabbit mAb**	WB	sc-3010	SCBT
**Alzheimer's Disease Antibody Sampler Kit**	WB 1/1000 for pirmaries, 1/2500 for anti-Ms and Rb se	CST.9784S	CST
**Phospho-Tau (Ser396) (PHF13) Mouse mAb**	WB 1/1000, 5% CST-BSA	CST.9632S	CST
**β-Amyloid (pE3 Peptide) (D5N5H) Rabbit mAb**	WB:1/1000, IF: 1/200	CST.14975P	CST
**Phospho-Tau (Thr212) (PHF13) Mouse mAb**	WB:1/1000, 5% CST-BSA	MBS128512	CST
**BACE**	WB 1/1000 for pirmaries, 1/2500 for anti-Ms and Rb se	D10E5	CST
**AT270 (P-Tau Thr181)**	IF/WB	MN-1080	Thermo
**TUBB3 (Rabbit)**	IF/IB	SAB4300623	Sigma
**TUBB3 (Guinea pig)**	IF	302304	Synaptic Systems
**GFAP**	IF/WB	Z0334	Dako
**MC1**	1/250 (IF)	N/A	P. Davies
**AT8**	1/400 (IF)	MN1020	Thermo

Supplemental references

Briggs, J.A., Sun, J., Shepherd, J., Ovchinnikov, D.A., Chung, T.L., Nayler, S.P., Kao, L.P., Morrow, C.A., Thakar, N.Y., Soo, S.Y., *et al.* (2012). Integration-free induced pluripotent stem cells model genetic and neural developmental features of down syndrome etiology. Stem Cells *31*, 467-478.

Briggs, J.A., Sun, J., Shepherd, J., Ovchinnikov, D.A., Chung, T.L., Nayler, S.P., Kao, L.P., Morrow, C.A., Thakar, N.Y., Soo, S.Y., *et al.* (2013). Integration-free induced pluripotent stem cells model genetic and neural developmental features of down syndrome etiology. Stem Cells *31*, 467-478.

Chambers, S.M., Fasano, C.A., Papapetrou, E.P., Tomishima, M., Sadelain, M., and Studer, L. (2009). Highly efficient neural conversion of human ES and iPS cells by dual inhibition of SMAD signaling. Nature biotechnology *27*, 275-280.

Chen, E.Y., Tan, C.M., Kou, Y., Duan, Q., Wang, Z., Meirelles, G.V., Clark, N.R., and Ma'ayan, A. (2013). Enrichr: interactive and collaborative HTML5 gene list enrichment analysis tool. BMC Bioinformatics *14*, 128.

Du, P., Kibbe, W.A., and Lin, S.M. (2008). lumi: a pipeline for processing Illumina microarray. Bioinformatics *24*, 1547-1548.

Dumevska, B., Bosman, A., McKernan, R., Main, H., Schmidt, U., and Peura, T. (2016a). Derivation of Trisomy 21 affected human embryonic stem cell line Genea021. Stem cell research *16*, 401-404.

Dumevska, B., Bosman, A., McKernan, R., Schmidt, U., and Peura, T. (2016b). Derivation of human embryonic stem cell line Genea022. Stem cell research *16*, 472-475.

Huber, W., Carey, V.J., Gentleman, R., Anders, S., Carlson, M., Carvalho, B.S., Bravo, H.C., Davis, S., Gatto, L., Girke, T., *et al.* (2015). Orchestrating high-throughput genomic analysis with Bioconductor. Nature methods *12*, 115-121.

Kauffmann, A., Gentleman, R., and Huber, W. (2009). arrayQualityMetrics--a bioconductor package for quality assessment of microarray data. Bioinformatics *25*, 415-416.

Kuleshov, M.V., Jones, M.R., Rouillard, A.D., Fernandez, N.F., Duan, Q., Wang, Z., Koplev, S., Jenkins, S.L., Jagodnik, K.M., Lachmann, A., *et al.* (2016). Enrichr: a comprehensive gene set enrichment analysis web server 2016 update. Nucleic Acids Res *44*, W90-97.

Lachmann, A., Xu, H., Krishnan, J., Berger, S.I., Mazloom, A.R., and Ma'ayan, A. (2010). ChEA: transcription factor regulation inferred from integrating genome-wide ChIP-X experiments. Bioinformatics *26*, 2438-2444.

Letourneau, A., Santoni, F.A., Bonilla, X., Sailani, M.R., Gonzalez, D., Kind, J., Chevalier, C., Thurman, R., Sandstrom, R.S., Hibaoui, Y., *et al.* (2014). Domains of genome-wide gene expression dysregulation in Down's syndrome. Nature *508*, 345-350.

Martinez de Paz, A., and Ausio, J. (2016). HMGNs: The enhancer charmers. BioEssays : news and reviews in molecular, cellular and developmental biology *38*, 226-231.

Ovchinnikov, D.A., Hidalgo, A., Yang, S.K., Zhang, X., Hudson, J., Mazzone, S.B., Chen, C., Cooper-White, J.J., and Wolvetang, E.J. (2015). Isolation of contractile cardiomyocytes from human pluripotent stem-cell-derived cardiomyogenic cultures using a human NCX1-EGFP reporter. Stem cells and development *24*, 11-20.

Ovchinnikov, D.A., Titmarsh, D.M., Fortuna, P.R., Hidalgo, A., Alharbi, S., Whitworth, D.J., Cooper-White, J.J., and Wolvetang, E.J. (2014). Transgenic human ES and iPS reporter cell lines for identification and selection of pluripotent stem cells in vitro. Stem cell research *13*, 251-261.

Ritchie, M.E., Phipson, B., Wu, D., Hu, Y., Law, C.W., Shi, W., and Smyth, G.K. (2015). limma powers differential expression analyses for RNA-sequencing and microarray studies. Nucleic acids research *43*, e47.

Weick, J.P., Kang, H., Bonadurer, G.F., 3rd, and Bhattacharyya, A. (2016). Gene Expression Studies on Human Trisomy 21 iPSCs and Neurons: Towards Mechanisms Underlying Down's Syndrome and Early Alzheimer's Disease-Like Pathologies. Methods in molecular biology *1303*, 247-265.

Wu, Y., Zhang, S., Xu, Q., Zou, H., Zhou, W., Cai, F., Li, T., and Song, W. (2016). Regulation of global gene expression and cell proliferation by APP. Sci Rep *6*, 22460.

Zempleni, J., Li, Y., Xue, J., and Cordonier, E.L. (2011). The role of holocarboxylase synthetase in genome stability is mediated partly by epigenomic synergies between methylation and biotinylation events. Epigenetics *6*, 892-894.
Zempleni, J., Liu, D., Camara, D.T., and Cordonier, E.L. (2014). Novel roles of holocarboxylase synthetase in gene regulation and intermediary metabolism. Nutrition reviews *72*, 369-376.
