# Supplementary material for: The Impact of APP on Alzheimer-like Pathogenesis and Gene Expression in Down Syndrome iPSC-Derived Neurons
Source: Stem Cell Reports. 2018 May 31;11(1):32–42. doi: 10.1016/j.stemcr.2018.05.004 (PMC6066957; doi:10.1016/j.stemcr.2018.05.004)
Supplement: Document S2. Article plus Supplemental Information [file mmc2.pdf]

Stem Cell Reports
Report

OPEN ACCESS

The Impact of APP on Alzheimer-like Pathogenesis and Gene Expression in Down Syndrome iPSC-Derived Neurons

Dmitry A. Ovchinnikov,[1] Othmar Korn,[1] Isaac Virshup,[2] Christine A. Wells,[1,2] and Ernst J. Wolvetang[1,*]
[1]Australian Institute for Bioengineering and Nanotechnology, University of Queensland, Brisbane, QLD 4072, Australia
[2]Centre for Stem Cell Systems, MDHS, University of Melbourne, Melbourne, VIC 3010, Australia
*Correspondence: e.wolvetang@uq.edu.au
https://doi.org/10.1016/j.stemcr.2018.05.004

SUMMARY

Early-onset Alzheimer disease (AD)-like pathology in Down syndrome is commonly attributed to an increased dosage of the amyloid precursor protein (APP) gene. To test this in an isogenic human model, we deleted the supernumerary copy of the *APP* gene in trisomic Down syndrome induced pluripotent stem cells or upregulated APP expression in euploid human pluripotent stem cells using CRISPRa. Cortical neuronal differentiation shows that an increased APP gene dosage is responsible for increased β-amyloid production, altered Aβ42/40 ratio, and deposition of the pyroglutamate (E3)-containing amyloid aggregates, but not for several tau-related AD phenotypes or increased apoptosis. Transcriptome comparisons demonstrate that *APP* has a widespread and temporally modulated impact on neuronal gene expression. Collectively, these data reveal an important role for APP in the amyloidogenic aspects of AD but challenge the idea that increased APP levels are solely responsible for increasing specific phosphorylated forms of tau or enhanced neuronal cell death in Down syndrome-associated AD pathogenesis.

INTRODUCTION

The *APP* gene encodes the amyloid precursor protein (APP) and is located on chromosome 21. Increased dosage of this gene results in an elevated expression of APP in Down syndrome (DS; trisomy 21) tissues (Oyama et al., 1994). This is thought to increase the levels of β-amyloid (Aβ), a cleavage product of APP that aggregates upon misfolding, accumulates in plaques in the brains of people with Alzheimer disease (AD) and DS (Braak and Braak, 1994), and in turn is assumed to underlie the development of early-onset, highly penetrant, AD-like pathology in individuals with DS (Decourt et al., 2013). Aβ aggregation was previously linked to tau hyperphosphorylation, defective synapse function, oxidative stress, and increased neuronal cell death (Spires-Jones and Hyman, 2014). Consistent with these observations, three cases of partial trisomy of chromosome 21 that exclude the *APP* locus showed no evidence of early-onset AD (EOAD) (Korbel et al., 2009) or neurodegeneration at an advanced age (Doran et al., 2017). Similarly, individuals with a rare familial duplication of the *APP* locus develop EOAD, although this is more akin to vascular dementia than classical AD (Rovelet-Lecrux et al., 2007; Sleegers et al., 2006). Individuals with DS can, nevertheless, carry large plaque loads without overt AD signs (Vemuri et al., 2010), challenging a direct causal relationship between *APP* triplication and EOAD in DS. Indeed, the penetrance and expressivity of disease phenotypes, including AD-like pathology, vary between DS individuals, and this has been attributed to the presence of modifier alleles on Hsa21 (e.g., *DYRK1A*, *BACE2*, *miR-155*) or other chromosomes, such as *APOE* (Sherman et al., 2007). Several groups have generated induced pluripotent stem cells (iPSCs) from individuals with DS (e.g., Shi et al., 2012). We (Briggs et al., 2013) and others (Murray et al., 2015) have previously found that nuclear reprogramming permits the isolation of isogenic euploid (Hsa21-disomic) iPSCs from otherwise fully Hsa21-trisomic DS subjects. DS iPSC-derived cortical neurons were previously shown to exhibit increased production of Aβ42, and hyperphosphorylation and redistribution of tau (Chang et al., 2015), suggesting that DS iPSC-derived cortical neuronal cultures can recapitulate aspects of AD neuropathology *in vitro* (Shi et al., 2012).

To elucidate the role of APP in EOAD in DS without potential confounding effects of modifier alleles, we manipulated APP dosage and expression in isogenic DS or euploid iPSC backgrounds; subjected these cell lines to prolonged cortical differentiation; and analyzed gene expression-, amyloid-, and tau-associated changes. Our data reveal APP gene dosage in DS has neurodevelopmental stage-specific, genome-wide gene regulatory effects and affects the Aβ42/Aβ40 ratio and pyroglutamate aggregates but does not alter a range of tau-phosphorylation events, abundance of neurofibrillary tangle (NFT)-like tau aggregates, or neuronal cell death.

RESULTS

Generation of APP Copy-Number-Normalized DS iPSCs and Doxycycline-Inducible *APP*-Overexpressing Human Embryonic Stem Cells

To address the role of the supernumerary copy of the APP gene in AD-like neuropathology in DS, we deleted exon 3

 © 2018 The Author(s).
This is an open access article under the CC BY license (http://creativecommons.org/licenses/by/4.0/).

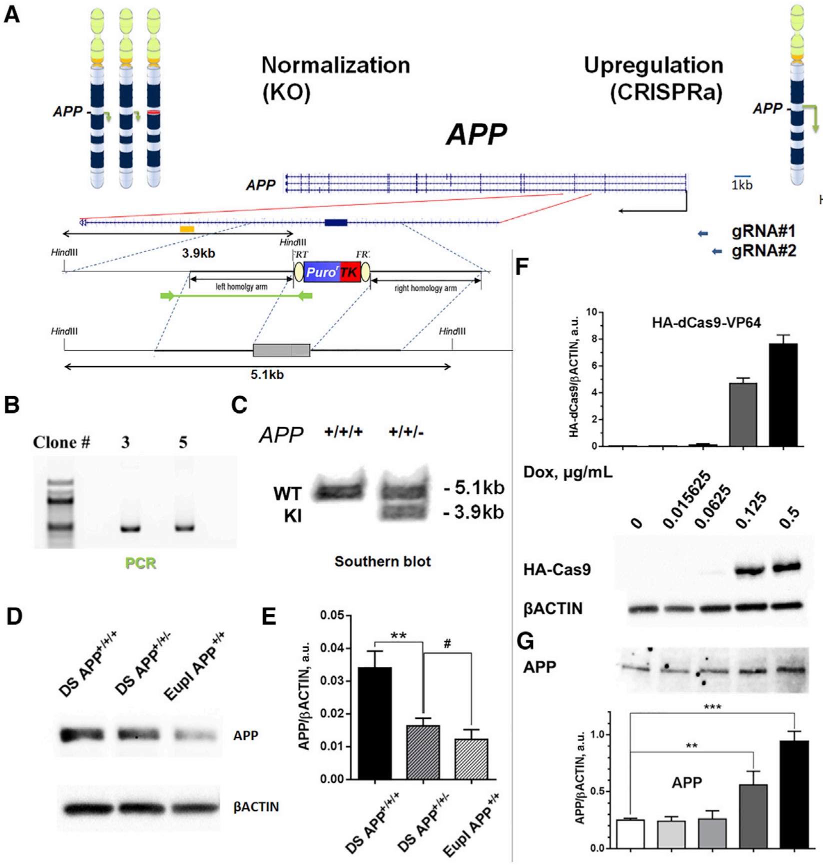

Figure 1. Manipulating *APP* Levels in Human Pluripotent Stem Cells Using CRISPR/Cas9-Aided Approaches

(A) Vector design for targeting exon 3 of the *APP* gene. Green is PCR product, yellow rectangle is location of the Southern probe. gRNA, guide RNA; KO, knockout.

(B) Targeted allele-specific PCR.

(C) Southern blot showing *APP* targeted allele in DS18 iPSC. KI, knockin; WT, wild-type.

(D and E) (D) Inactivationof one of the three *APP* alleles in day 45 DS iPSC-derived neurons reduces APP protein expression to isogenic euploid control levels (quantified in E, N = 3).

(F) Doxycycline induced upregulation of HA-tagged dCas9-VP64 in Gen22::TRE-dCas9-VP64 with HA antibody.

(G) Doxycycline induced APP protein expression in Gen22::TRE-dCas9-VP64 hESC (APP gRNA#1 shown).

**$p < 0.01$, ***$p < 0.001$, #non-significant; n = 3, n = 3 for qPCR and western blots. Means ± SEM values shown.

of one of the APP alleles (Figure 1A) in a previously characterized footprint-free DS iPSC line, clone C11DS (Briggs et al., 2013) and this was confirmed by genomic PCR (Figure 1B) and Southern blotting (Figure 1C). As expected, the ~1.5–2-fold increase in APP protein expression observed in neuronally differentiated DS APP$^{+/+/+}$ iPSC was "normalized" to euploid levels in the isogenic CRISPR-targeted APP$^{+/+/-}$ DS iPSC-derived neuronal cultures (Figures 1D and 1E), and this was maintained during prolonged neuronal differentiation (up to 90 days tested; Figure S3A).

APP overexpression in a euploid (Hsa21-disomic) background was achieved through lentiviral delivery of a doxycycline-inducible CRISPRa-driven system. We isolated a clonal line that displays tightly controlled dox-inducible HA-dCAS9-VP64 (Figure 1F) and APP (Figure 1G) expression, following lentiviral delivery of guide RNAs (gRNAs) that target the APP promoter (Figure S1J and Supplemental Experimental Procedures). The APP$^{+/+/-}$ iPSC and the Genea22::HA-dCAS9-VP64 line were devoid of chromosomal abnormalities (SNP arrays) and showed the hallmarks of pluripotent stem cells (Figure S1).

Neurogenic cultures derived from all six isogenic iPSC clones (2 DS APP$^{+/+/+}$, 1 DS APP$^{+/+/-}$, and three euploid APP$^{+/+}$ lines) displayed similar cortical neuronal trajectories with little contamination of non-neuronal cell types, and were transcriptionally most similar to the frontal cortex of a 16–18-week fetal brain (Figure S2A). Temporal changes in *TUBB3* and *TBR2* mRNA expression (Figure S2B), and immune-fluorescent detection of pan-neuronal markers TUBB3 and NeuN and astrocyte marker GFAP (Figure S2C), indicates comparable neuronal differentiation trajectories of all six isogenic lines. This is supported by the transcriptome-based temporal and regional staging of the neuronal cultures using CoNTExT (Figure S2), although a small decrease in cortical layer markers SATB2 and TBR1 (Figure S2C′) was detected in DS samples.

APP Copy Number Has a Significant Impact on the Transcriptome of DS Cortical Neuronal Cultures

Hierarchical clustering of microarray transcriptome data from DS APP$^{+/+/+}$ and isogenic euploid day 65 samples shows clustering is dictated by the presence of Hsa21 (Figure 2A). As expected, Hsa21 genes are highly overrepresented among significantly overexpressed transcripts

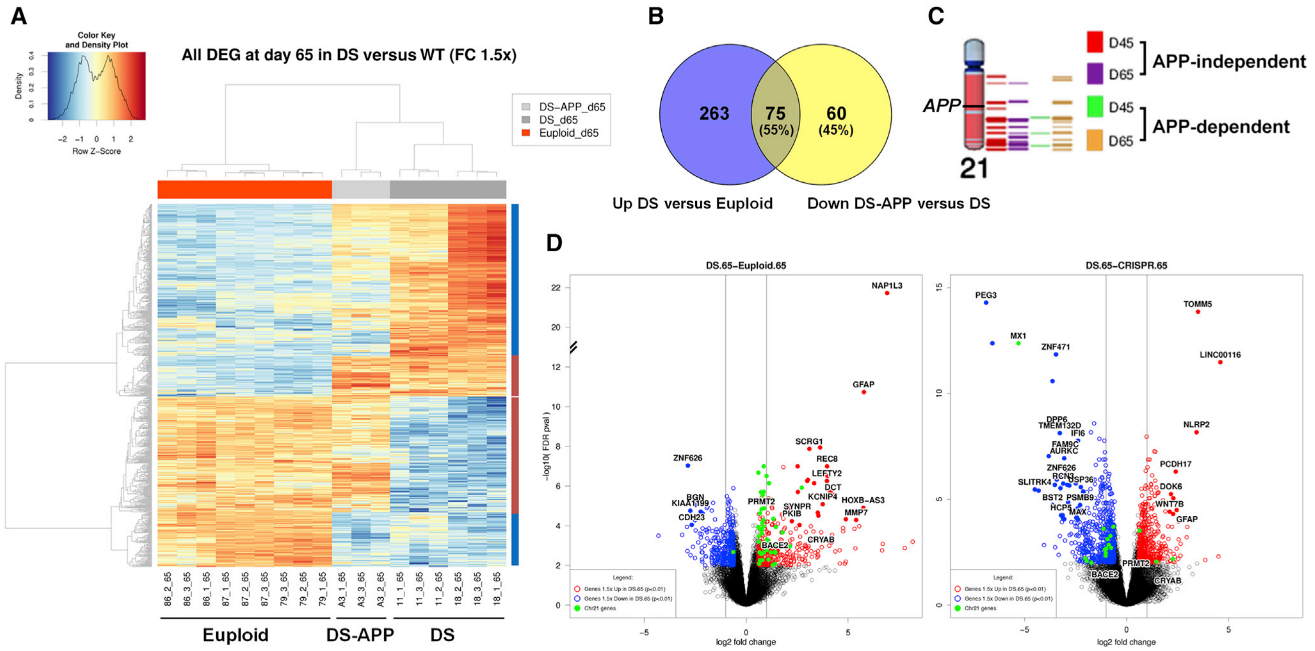

Figure 2. Transcriptome Profiling of Isogenic iPSC-Derived Neuronal Cultures at Day 65 of Differentiation

(A) Hierarchical probe clustering-based heatmaps (with limits set at 1.5-fold changes and $p < 0.01$) in day 65 neuronal cultures from three euploid, two DS, and one APP-normalized DS iPSC lines.

(B) Venn diagram illustrating overlap between DEG lists from DS-euploid and DS APP copy-adjusted comparisons.

(C) Graphic representation of Hsa21 genes significantly upregulated in APP-independent and APP-dependent manners at indicated days of differentiation.

(D) Volcano plots exemplifying asymmetrical changes in gene expression levels at day 65, with a larger number of genes upregulated in DS versus euploid, including numerous Hsa21 genes (shown in green). Similarly, a large number of genes are downregulated upon inactivation of the third *APP* copy in day 65 DS iPSC neurons (right panel).

(green dots in volcano plot in Figure 2D), and day 45 gene expression data show similar trends (Figures S2D and S2E). At both time points, chromosome 21 genes are significantly overrepresented among upregulated differentially expressed genes (DEGs) in Hsa21-trisomic cells ($p < 0.0002$), particularly those in the distal part of the long arm (Figure 2C). The relative contribution of Hsa21 genes diminishes from 22% to 15% from day 45 to 65 (see GEO dataset for chromosomal assignment details). DS and AD were identified as the top disease signatures (Table S1), and Ingenuity Pathway Analysis identified neurological signs, cognitive impairment, and abnormality of the cerebral cortex (Table S2). We next examined the transcriptome differences driven by the presence of the supernumerary APP gene copy in an isogenic Hsa21-trisomic context. Hierarchical clustering analyses revealed that the impact of the supernumerary *APP* copy number on the trisomy 21 transcriptome increases significantly from day 45 (Figure S2D) to day 65 (Figure 2A). By day 65 about half of all genes downregulated upon inactivation of the supernumerary *APP* gene in DS neurons were also abnormally upregulated in DS neurons (Figure 2B). This substantial impact of the APP gene on DS (Hsa21-trisomy)-associated gene expression changes in neurons particularly affects the expression of other Hsa21 genes (Figures 2C and 2D).

APP Copy Number Dictates Aβ Levels, Presence of Pyroglutamate Aggregates, and Neurite Length of DS iPSC-Derived Cortical Neurons

APP, when processed by the γ- and β-secretases, can generate the amyloidogenic Aβ42 peptide or the more abundant and non-cytotoxic Aβ40 peptide. Because these proteases are expressed (as confirmed by our microarray and qPCR data; Figure S3D), increased APP levels are expected to result in elevated neuronal Aβ amyloid production (Maulik et al., 2015) and an increased ratio of Aβ42/Aβ40 peptides. In day 90 DS iPSC-derived cortical neurons, we indeed detected an increased secretion of both Aβ42 (Figure 3A) and Aβ40 (Figure S3C) in the culture medium compared with $APP^{+/+}$ (euploid) cells, and these levels were normalized to euploid levels in $APP^{+/+/-}$ neuronal cultures. Upregulation of Aβ42 was also observed in the medium of Genea21 DS human embryonic stem cell (hESC)-derived neurons compared with the euploid Genea22

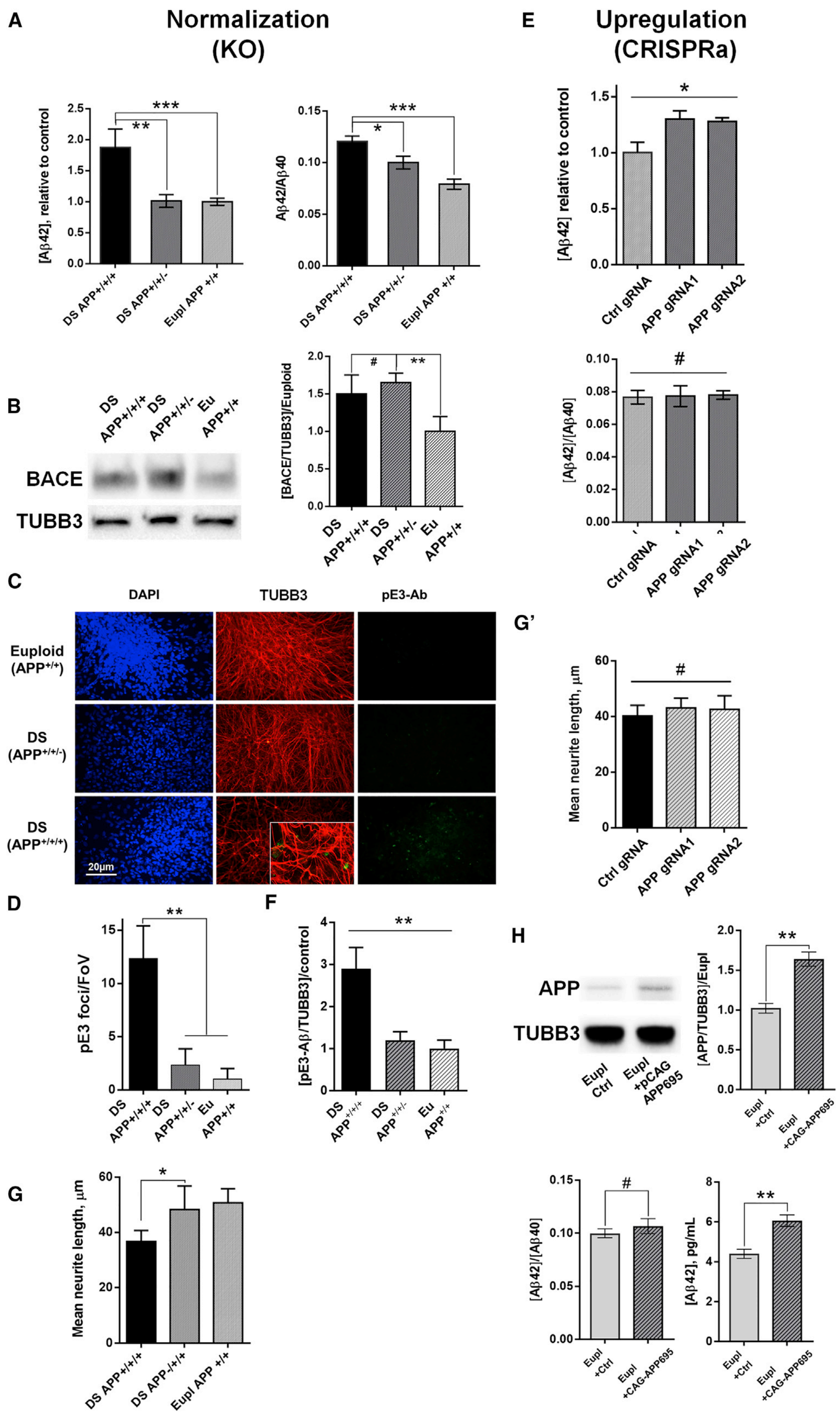

(legend on next page)

sibling hESC-derived neurons, but this was accompanied by little (day 45) to no (day 90) skewing of the Aβ42/Aβ40 ratio (Figure S3B). The correction of APP gene dosage ($APP^{+/+/-}$ DS iPSC) reversed the elevated Aβ42/Aβ40 ratio observed in DS iPSC-derived neuronal cultures (Figure 3A), albeit only partially, suggesting APP expression may indirectly affect the activity of the processing machinery. Indeed, mRNA and protein levels of β-secretase BACE2, an Hsa21 gene already elevated in day 65 DS neurons (and DS brains; e.g., Cheon et al., 2008), becomes even further elevated in DS $APP^{+/+/-}$ neurons (Figure 3B). Since BACE2 activity potentially reduces the substrate pool for BACE1 (Sun et al., 2006), this may account for the reduction in Aβ40 and thus the only partially normalized Aβ42/Aβ40 ratio observed in APP copy-number-normalized DS neurons (Figure S3E). Inhibition of BACE activity with 1 or 10 nM verubecestat reduced Aβ42 production (Figure S3F) and Aβ42/Aβ40 ratios in neuronal cultures from $APP^{+/+/+}$ DS, $APP^{+/+/-}$ DS, and isogenic euploid iPSCs (Figure S3E), confirming that BACE activity is responsible.

We next show that increasing the expression of APP in Genea022::TRE-dCas9-VP64 hESC-derived day 90 neurons was sufficient to increase both secreted Aβ42 and Aβ40 peptide levels but did not alter the Aβ42/Aβ40 ratio (Figure 3E). In euploid iPSC (Eu79)-derived neurons, 1.5-fold overexpression of APP using an APP695-expressing plasmid similarly upregulated Aβ42 peptide levels but also did not change the Aβ42/Aβ40 ratio (Figure 3H).

Collectively these data show that APP copy number is necessary for increased Aβ42 levels and skewing of the Aβ42/Aβ40 ratio in DS neurons, and that increasing APP expression in euploid neurons is sufficient to increase Aβ42 peptide levels but does not alter the Aβ42/Aβ40 ratio in this model.

Oligomeric Aβ42 is considered to be a key cytotoxic agent in AD, particularly after it heterodimerizes with N-terminally truncated pyroglutamylated Aβ (Gunn et al., 2010). This further processed species is more aggregation prone and can make up half of the amyloid load in AD brains (Gunn et al., 2016). We detected increased numbers of pyroglutamate (pE3)-positive foci in 120 day DS iPSC-derived neuronal cultures (Figure 3C) and increased protein expression by western blot (Figures 3D and S3G), which were both reduced to near-euploid levels upon removal of the supernumerary copy of the APP gene. CRISPRa-driven overexpression of *APP* in euploid Gen22 hESC was not sufficient to cause an appreciable increase in the pyroglutamate (pE3) levels (Figure S3H).

APP is also known to play a role in regulation of the outgrowth of neuronal processes. N-terminally processed extracellular fragments (e.g., sAPPα) can enhance neurite outgrowth, whereas C-terminally derived forms can exert opposite effects (Trazzi et al., 2013; Young-Pearse et al., 2008). Normalization of *APP* copy number in a DS chromosomal context increased mean neurite length by ~18% (day 120 neurons), to a size similar to that of isogenic euploid controls (Figure 3G), suggesting that in DS the extra copy of APP limits neurite outgrowth. CRISPRa-directed upregulation of *APP* in euploid neurons did not increase neurite length (Figure 3G').

APP and Aβ42 Levels Do Not Directly Control Neuronal Cell Death or Tau-Hyperphosphorylation Sites Linked to Neurodegeneration

Aβ42 is thought to be responsible for increasing neuronal cell death by increasing oxidative stress (Manterola et al., 2013). Hydrogen peroxide-induced apoptosis, a measure of cellular resilience to oxidative stress, is indeed increased in primary DS fetus-derived neurons (Busciglio and Yankner, 1995). In agreement with these observations we find that day 90 DS neurons also exhibit increased hydrogen peroxide-induced apoptosis (Figures 4A and 4B). However, hydrogen peroxide sensitivity was not corrected by normalization of *APP* gene copy number in either day 45 (Figure 4A) or day 90 cultures (Figure 4B). Increasing the expression of APP in hESCs, which increases Aβ42 levels, only very modestly increased apoptosis in day 90 neuronal cultures (Figure 4C). We conclude that increased *APP* gene

Figure 3. APP Levels Influence β-Amyloidogenic AD-like Phenotypes and Neurite Outgrowth in Neuronal Cultures
(A) Levels of secreted Aβ42 and Aβ42/Aβ40 peptide ratios measured in medium conditioned by 90-day-old neuronal cultures of respective genotypes (N = 3).
(B) Western blot detection of BACE protein in APP^{+++}, APP^{++-} DS and isogenic euploid neurons.
(C) Day 120+ DS neuronal cultures display more pE3-pyroglutamate-Aβ immunoreactive foci than euploid or APP copy-number-corrected DS cultures.
(D) Quantification of the numbers of pE3-pyroglutamate foci (per field of view).
(E) CRISPRa-driven overexpression of APP leads to an increase in Aβ42 levels but does not alter the Aβ42/Aβ40 ratio (day 90).
(F) Quantification of pyroglutamate expression relative to TUBB3, as measured by western blotting.
(G) The effect of APP copy number on neurite length in day 120 DS neurons and (G′) in euploid neurons with CRISPRa-upregulated APP.
(H) Overexpression of APP in day 90 euploid neurons using APP695 ORF results in elevated Aβ42 levels but does not alter the Aβ42/Aβ40 ratio. Means ± SEM (N = 3).
*$p < 0.05$, **$p < 0.01$, ***$p < 0.001$, #non-significant. Scale bar in (C) represents 20 μm.

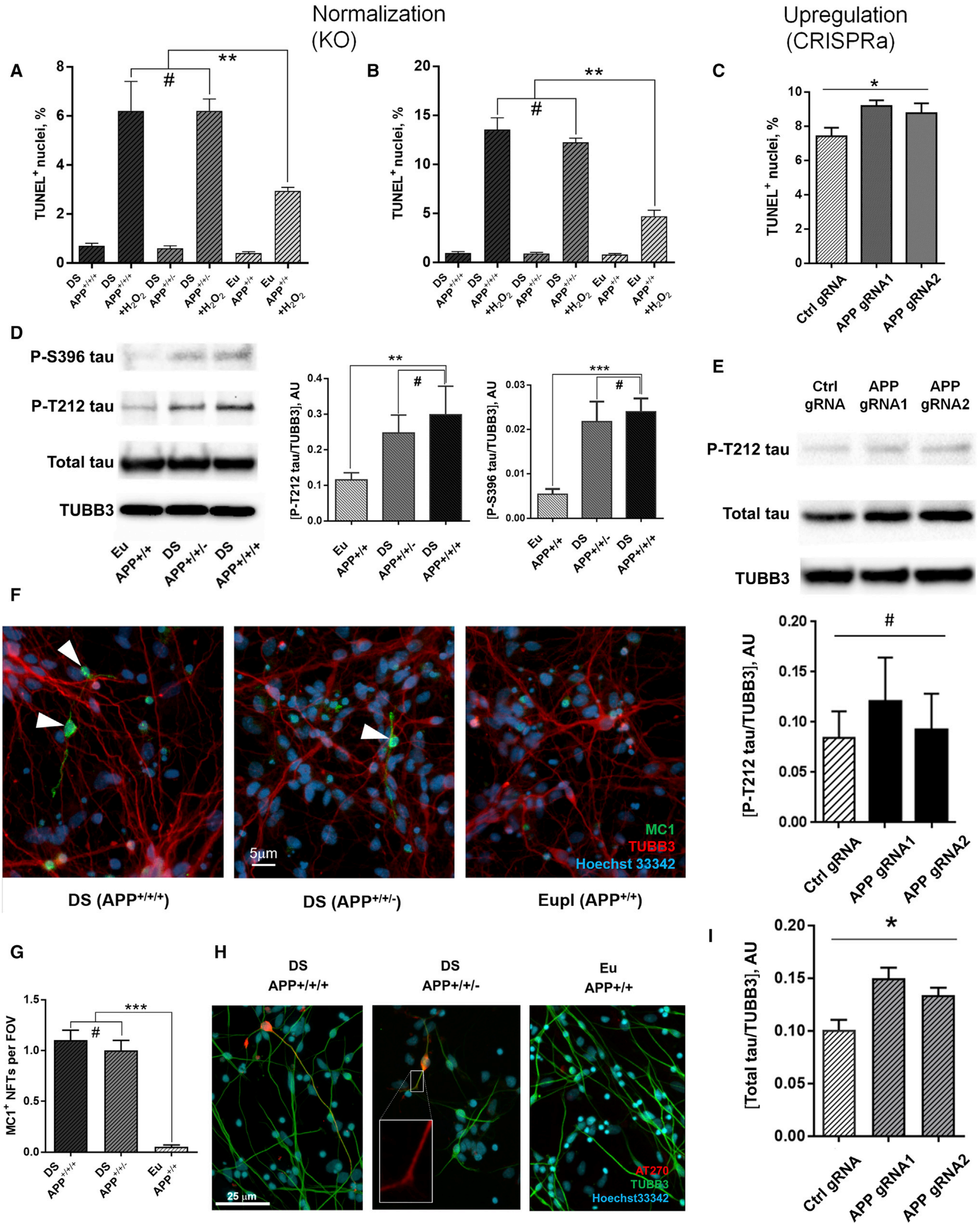

(legend on next page)

load, and increased Aβ42 and pE3 pyroglutamate levels, are not directly responsible for the increased oxidative stress-induced cell death of DS neurons under these conditions.

We next explored whether APP gene load and Aβ42 levels affect tau phosphorylation in day 90 neurons from isogenic DS, euploid, and APP-normalized DS iPSCs. To account for potential differences in tau protein levels themselves or altered neuronal content of the samples, the fractions of P-S396 and P-T212 tau (canonical sites implicated in AD) were normalized to either total tau or the neuronal marker βIII-tubulin (TUBB3). Irrespectively, P-S396 and P-T212 tau levels were significantly higher in DS neurons, but these were not reduced by normalization of APP gene dosage (Figure 4D). These data therefore strongly suggest that the increased levels of Aβ42 and skewed Aβ42/Aβ40 ratio in DS neuronal cultures are not directly responsible for increasing these tau-phosphorylation events, and this is consistent with our observation that normalization of APP also did not affect apoptosis, a process previously linked to tau phosphorylation (Wu et al., 2016). Immunofluorescent detection of hyperphosphorylated tau with the AT270 antibody revealed an increase in immunoreactivity in both cell bodies and axons in a subset of day 120 DS $APP^{+/+/+}$ neurons compared with isogenic $APP^{+/+}$ euploid neurons, but this was not reduced in DS $APP^{+/+/-}$ cultures (Figure 4H). Similar data were obtained with the AT8 phospho-tau antibody (Figure S4A). Detection of pathological tau with the conformation-specific antibody MC1 revealed potential early NFT-like structures in day 120 DS neurons that were absent in isogenic euploid neurons but persisted in DS $APP^{+/+/-}$ neuronal cultures (Figures 4F and 4G). CRISPRa upregulation of *APP* to DS levels in euploid hESCs over 90 days of neuronal differentiation did not lead to increased tau T212-phosphorylation (Figure 4E). This does lead to a modest upregulation of total tau in these neurons (Figure 4E), in agreement with previously reported effects of APP on tau proteostasis (Moore et al., 2015). Collectively our data suggest that the increased APP and Aβ42 levels do not affect the increased abundance of neuro-pathology-associated species of phosphorylated tau or apoptotic sensitivity of DS neurons.

DISCUSSION

In iPSC-derived DS neurons, APP mRNA and protein levels are ~2-fold increased over the entire neuronal differentiation time course, in agreement with elevated gene dosage and previous reports (Buxbaum and Greengard, 1996). We show that inactivating one copy of the *APP* gene in a trisomy 21 background normalized APP, secreted levels of Aβ42, Aβ42/Aβ40 ratio (at least in part), and pyroglutamate (pE3)-containing Aβ levels and foci numbers to euploid levels. This indicates that increased expression of APP is directly responsible for these hallmark features of AD pathogenesis in this *in vitro* DS model. A considerable body of literature suggests that Aβ42 (either aggregated or in its monomeric form) directly or indirectly induces tau hyperphosphorylation and that this in turn leads to synaptic defects, neuronal dysfunction, and cell death (e.g., Gotz et al., 2001). Our data show that normalization of APP copy number and Aβ42 levels in DS cortical neurons does not reduce tau phosphorylation, nor does it affect the sensitivity of neurons to oxidative stress-induced apoptosis. Furthermore, overexpression of APP to physiologically relevant levels (1.5–2-fold) in euploid cortical neurons increased Aβ42 but was not sufficient to increase amyloid pyroglutamate E3 levels, or tau (T212) hyperphosphorylation, and only very moderately increased oxidative stress-induced apoptosis. APP overexpression by itself did not significantly skew the Aβ42/40 ratio, suggesting that other Hsa21 genes might attenuate APP processing.

These data challenge the notion of a direct linear relationship between APP/Aβ42 levels and tau pathology-induced

Figure 4. Hyperphosphorylation of Tau and Apoptosis Are Not Controlled by the Levels of *APP* Gene Expression
(A) Hydrogen peroxide-induced cell death measured by fluorescence-activated cell sorting of TUNEL-labeled cells in DS neuronal cultures at days 45.
(B and C) (B) Day 90 DS cultures and (C) day 90 Gen22::TRE-dCas9-VP64 hESC-derived neurons (gRNAs as labeled).
(D) Western blots of P-T212, P-S396, and total Tau in day 90 DS, APP copy-number-corrected, and isogenic euploid neurons and their quantification relative to TUBB3.
(E) Western blotting of day 90 Gen22::TRE-dCas9-VP64 hESC-derived neurons with T212 Tau antibody and quantification relative to total Tau.
(F) Overlaid images of $MC1^{+}$ (paired helical filament-containing) NFT-like aggregates (highlighted by arrowheads) in Hsa21-trisomic, APP copy-number-normalized, and euploid day 120 cortical neurons.
(G) Quantification of the frequency of $MC1^{+}$ NFT-containing neurons per field of view in D120 cortical neuronal cultures.
(H) Detection of hyperphosphorylated tau accumulation using AT270 antibody in day 120 DS, APP copy-number-corrected, and isogenic euploid neurons.
(I) Levels of total Tau protein in day 90 Genea22::TRE-dCas9-VP64 hESC-derived neurons overexpressing APP, relative to TUBB3.
*$p < 0.05$, **$p < 0.01$, ***$p < 0.001$, #non-significant. Values shown are means ± SEM of three experiments.

neuronal cell death in this DS model. In agreement with this notion, mouse models that display typical Aβ pathology with amyloid plaque deposition, such as mice with a humanized *App* gene (Saito et al., 2014) or overexpression of wild-type APP protein (Cataldo et al., 2003), also did not exhibit tau pathology. In agreement with previous observations (Moore et al., 2015), APP gene copy number affects tau proteostasis, albeit modestly. Interestingly, we do not observe this in DS iPSC-derived neurons, suggesting compensatory effects of the dosage of other Hsa21 genes or other compensatory processes (Simon et al., 2012).

Collectively these data underline the need for a better understanding of the role(s) of other Hsa21 genes and elucidation of the intra-chromosomal and genome-wide gene regulatory networks they affect. Our gene expression data comparing isogenic euploid, DS, and APP copy-number-normalized DS neurons at days 45 and 65 of cortical differentiation highlight a number of important concepts in this regard. The initial idea that DS is exclusively driven by the overexpression of all Hsa21 genes or by genes that reside in a DS critical region (Korbel et al., 2009) has been superseded by more sophisticated models that take into account intra- and inter-chromosomal gene regulatory networks as well as epigenetic effects (Antonarakis, 2017). We now show that the number, identity, and expression levels of overexpressed Hsa21 genes in DS neurons vary during different temporal windows of neuronal differentiation, a concept well accepted in developmental biology. The corollary of this observation is that each cell type at each stage of development may be differentially affected by overexpression of specific combinations of Hsa21 genes (either directly, or indirectly by altering the expression of genes on other chromosomes, or by affecting the global epigenetic landscape), adding further complexity to understanding the etiology of DS-associated pathologies.

At later time points (days 90–120) we find that presence of a supernumerary APP copy decreases neurite length in a trisomic genetic background, in agreement with previous data in a DS mouse model (Trazzi et al., 2013). Our gene expression data reveal that a surprisingly large proportion of genes differentially expressed between euploid and DS neurons are controlled by APP copy number and that this proportion dramatically increases from day 45 to day 65. Remarkably, a disproportionally large fraction (~10%) of these APP-controlled genes reside on chromosome 21 (see GEO dataset for details).

The molecular mechanism that mediates this unexpected gene regulatory role of APP remains unclear at this stage. It has been suggested that APP can function as a ligand, act as a (co)-receptor (Deyts et al., 2016), and can release a Notch-like intra-cellular domain of APP (AICD) that can alter transcription following dimerization with intra-cellular partners such as Fe65 and Tip60 (Cao and Sudhof, 2001; Chang and Suh, 2010). However, when we examined the cohort of genes identified using ChIP (chromatin immunoprecipitation)-on-chip in neuroblastoma cells overexpressing AICD and Fe65 (Muller et al., 2007), we found very limited overlap with our sets of APP copy-number-dependent up- or downregulated genes in DS neurons.

Our functional data indicating that tau phosphorylation and neuronal cell death were not affected by APP copy number or Aβ42 normalization are supported by our bioinformatics analyses that show that genes that change their behavior in an APP-dependent fashion in a Hsa21-trisomic background overlap with pathways related to abnormal nervous system development (Enrichr portal, MP0003632, $p < 10^{-16}$) and abnormal brain morphology (MP0002152, $p < 10^{-11}$) but not with AD (Table S1). Conversely, genes that are differentially expressed between DS and euploid neurons and overexpressed independently of the *APP* gene copy number show high congruence with gene sets upregulated in DS at both days 45 and 65 (false discovery rate [FDR]-corrected p values $<6 \times 10^{-15}$ and $<3 \times 10^{-8}$, respectively, see Table S1), indicating that, in this *in vitro* model, Hsa21 genes other than APP drive many of the DS and AD pathogenic processes. Gene expression comparisons of DS and euploid neurons identified AD as the top disease, whereas comparison of DS with DS APP aligns with the "abnormal brain development and morphology"-associated gene list (input datasets shown in Tables S1A and S4A). Collectively, our data reveal that the *APP* gene plays an important role in moderating the expression of genes in *trans*, many of which reside on chromosome 21 itself, and that this changes during the course of cortical neuronal differentiation *in vitro*. We further demonstrate that the supernumerary copy of *APP* is indeed responsible for increased Aβ42 and pyroglutamate-containing amyloid levels in DS but is not directly involved in stimulation of tau hyperphosphorylation or increased neuronal cell death in DS neuronal cultures.

Systematic CRISPR-assisted genome manipulation in DS iPSC should permit the further elucidation of the transcriptional and putative epigenetic modulatory effects of APP and the roles and interactions of other Hsa21 genes involved in AD pathogenesis in DS, and provide further insight into the complex molecular mechanisms and gene regulatory networks underlying AD pathogenesis in DS and the general population.

EXPERIMENTAL PROCEDURES

Human Pluripotent Stem Cell Culture

Experiments were performed with approval by the University of Queensland Human Research Ethics Committee (HREC/09/QRCH/103, approval number 2015000667) and University of Queensland Animal Ethics Committee (AIBN/178/15/SCA/LEJEUNE/KACST).

Derivation of isogenic DS and euploid human iPSCs from skin fibroblasts was described (Briggs et al., 2013) and the near-isogenic hESC cell lines Genea021 and Genea022 (Dumevska et al., 2016a, 2016b) were obtained from Genea Biocells (San Diego, United States). Pluripotent cell cultures were maintained on Matrigel substrate (BD Biosciences, United States) in mouse embryonic fibroblast-conditioned knockout serum replacement (KOSR) medium supplemented with 100 μM β-mercaptoethanol and 100 ng/mL basic fibroblast growth factor (bFGF) (for iPSCs) or 50 ng/mL bFGF (for hESCs), and passaged by manual cutting or enzymatic dissociation using dispase as described (Ovchinnikov et al., 2015b).

Generation of the Cortical Neurogenic Cultures

Simultaneous inhibition of transforming growth factor β/activin and bone morphogenetic protein/growth differentiation factor signaling pathways was used to generate neurogenic cultures (Briggs et al., 2013; Chambers et al., 2009). Production of the neuroprogenitor and neuronal populations was performed using conventional approaches (see Supplemental Experimental Procedures), and maturation for 90- and 120-day-old cultures was achieved by supplementing the differentiation medium from day 65 with 20 ng/mL brain-derived neurotrophic factor, 10 ng/mL glial cell-derived neurotrophic factor, 500 nM ascorbic acid, and 1 mM dibutyl-cyclic AMP.

Transcriptome Analyses

Description of sample preparation and Illumina-HT12 microarray analysis is outlined in Supplemental Experimental Procedures. A $\log_2$ detection threshold of 3.2 was applied to the quantile normalized $\log_2$ data, retaining probes expressing above threshold in at least 50% of samples. After filtering, 17,259 probes were considered for a two-factor differential expression analysis (by phenotype and day) using Limma (Ritchie et al., 2015). Due to earlier removal of two outlying samples, a mixed linear model was applied, treating variance within cell line clones as random effects. Multiple pairwise contrasts between phenotypes and days of interest were fitted to the linear model and top tables of significant differential expression probes by empirical Bayes moderated t test were produced. Results were FDR $p < 0.01$ adjusted (Benjamini-Hochberg). A fold change threshold of 1.5 was used in heatmaps and volcano plots. Probes were annotated to genes using the hg19 Ensembl human genome assembly.

Immunofluorescence and Flow Cytometry

Staining with antigen-specific and fluorescent antibodies was performed essentially as described (Ovchinnikov et al., 2015a) with minor modifications (see Supplemental Experimental Procedures). Antibodies used in this study are listed in Supplemental Experimental Procedures.

Western Blotting

Western blotting of the Laemmli/RIPA-based protein lysates supplemented with protease and phosphatase inhibitors (Roche/Life Sciences, USA) was performed using the iBlot transfer system (Life Technologies, USA) and detected using Clarity ECL (Bio-Rad) according to the manufacturer's recommendations (see details in Supplemental Experimental Procedures).

Measuring Soluble Products of APP Proteolysis

To measure production of different forms of Aβ in the medium of neurogenic cultures of various genotypes, we utilized ELISA kits specifically aimed at detection of the two most relevant and dominant Aβ species: Aβ40 (KHB3482) and Aβ42 (KHB3441) (Thermo Fisher Scientific/Life Technologies). Medium was used in 1:20 dilution in ELISA buffer for direct measurement of absolute peptide concentration using the provided standard curves. Peptide concentrations were normalized to βIII-tubulin as measured by western blotting from cells that produced secreted APP products.

ACCESSION NUMBERS

The accession number for the dataset reported in this paper is GEO: GSE100680.

SUPPLEMENTAL INFORMATION

Supplemental Information includes Supplemental Experimental Procedures, four figures, and four tables and can be found with this article online at https://doi.org/10.1016/j.stemcr.2018.05.004.

AUTHOR CONTRIBUTIONS

D.A.O. and E.J.W. conceived the project and designed experiments; D.A.O. performed all wet-lab experiments and data analyses; C.A.W. contributed to experimental design; C.A.W., I.V., and O.K. contributed to bioinformatics analyses; D.A.O. and E.J.W. wrote the manuscript; all authors edited the manuscript.

ACKNOWLEDGMENTS

We acknowledge funding from the Australian Research Council's Special Research Initiative "Stem Cells Australia" and the Australian National Health and Medical Research Council grant (NHMRC APP1062802). We thank Rowland Mosbergen, Sally Martin, and Tyrone Chen for technical assistance, Drs. R. Medeiros and P. Davis for providing the MC1 antibody, and all researchers who deposited plasmids in the Addgene repository. This work was performed in part at the University of Queensland node of the Australian National Fabrication Facility, a company established under the National Collaborative Research Infrastructure Strategy to provide nano- and micro-fabrication facilities for Australia's researchers.

Received: August 21, 2017
Revised: May 3, 2018
Accepted: May 4, 2018
Published: May 31, 2018

REFERENCES

Antonarakis, S.E. (2017). Down syndrome and the complexity of genome dosage imbalance. Nat. Rev. Genet. *18*, 147–163.

Braak, H., and Braak, E. (1994). Morphological criteria for the recognition of Alzheimer's disease and the distribution pattern of cortical changes related to this disorder. Neurobiol. Aging *15*, 355–356, discussion 379–380.

Briggs, J.A., Sun, J., Shepherd, J., Ovchinnikov, D.A., Chung, T.L., Nayler, S.P., Kao, L.P., Morrow, C.A., Thakar, N.Y., Soo, S.Y., et al.

(2013). Integration-free induced pluripotent stem cells model genetic and neural developmental features of down syndrome etiology. Stem Cells *31*, 467–478.

Busciglio, J., and Yankner, B.A. (1995). Apoptosis and increased generation of reactive oxygen species in Down's syndrome neurons in vitro. Nature *378*, 776–779.

Buxbaum, J.D., and Greengard, P. (1996). Regulation of APP processing by intra- and intercellular signals. Ann. N. Y. Acad. Sci. *777*, 327–331.

Cao, X., and Sudhof, T.C. (2001). A transcriptionally active complex of APP with Fe65 and histone acetyltransferase Tip60. Science *293*, 115–120.

Cataldo, A.M., Petanceska, S., Peterhoff, C.M., Terio, N.B., Epstein, C.J., Villar, A., Carlson, E.J., Staufenbiel, M., and Nixon, R.A. (2003). App gene dosage modulates endosomal abnormalities of Alzheimer's disease in a segmental trisomy 16 mouse model of Down syndrome. J. Neurosci. *23*, 6788–6792.

Chambers, S.M., Fasano, C.A., Papapetrou, E.P., Tomishima, M., Sadelain, M., and Studer, L. (2009). Highly efficient neural conversion of human ES and iPS cells by dual inhibition of SMAD signaling. Nat. Biotechnol. *27*, 275–280.

Chang, C.Y., Chen, S.M., Lu, H.E., Lai, S.M., Lai, P.S., Shen, P.W., Chen, P.Y., Shen, C.I., Harn, H.J., Lin, S.Z., et al. (2015). N-butylidenephthalide attenuates Alzheimer's disease-like cytopathy in Down syndrome induced pluripotent stem cell-derived neurons. Sci. Rep. *5*, 8744.

Chang, K.A., and Suh, Y.H. (2010). Possible roles of amyloid intracellular domain of amyloid precursor protein. BMB Rep. *43*, 656–663.

Cheon, M.S., Dierssen, M., Kim, S.H., and Lubec, G. (2008). Protein expression of BACE1, BACE2 and APP in Down syndrome brains. Amino Acids *35*, 339–343.

Decourt, B., Mobley, W., Reiman, E., Shah, R.J., and Sabbagh, M.N. (2013). Recent perspectives on APP, secretases, endosomal pathways and how they influence Alzheimer's related pathological changes in Down syndrome. J. Alzheimers Dis. Parkinsonism (*Suppl 7*) https://doi.org/10.4172/2161-0460.S7-002.

Deyts, C., Thinakaran, G., and Parent, A.T. (2016). APP receptor? To be or not to be. Trends Pharmacol. Sci. *37*, 390–411.

Doran, E., Keator, D., Head, E., Phelan, M.J., Kim, R., Totoiu, M., Barrio, J.R., Small, G.W., Potkin, S.G., and Lott, I.T. (2017). Down syndrome, partial trisomy 21, and absence of Alzheimer's disease: the role of APP. J. Alzheimers Dis. *56*, 459–470.

Dumevska, B., Bosman, A., McKernan, R., Main, H., Schmidt, U., and Peura, T. (2016a). Derivation of trisomy 21 affected human embryonic stem cell line Genea021. Stem Cell Res. *16*, 401–404.

Dumevska, B., Bosman, A., McKernan, R., Schmidt, U., and Peura, T. (2016b). Derivation of human embryonic stem cell line Genea022. Stem Cell Res. *16*, 472–475.

Gotz, J., Chen, F., van Dorpe, J., and Nitsch, R.M. (2001). Formation of neurofibrillary tangles in P301l tau transgenic mice induced by Abeta 42 fibrils. Science *293*, 1491–1495.

Gunn, A.P., Masters, C.L., and Cherny, R.A. (2010). Pyroglutamate-Abeta: role in the natural history of Alzheimer's disease. Int. J. Biochem. Cell Biol. *42*, 1915–1918.

Gunn, A.P., Wong, B.X., Johanssen, T., Griffith, J.C., Masters, C.L., Bush, A.I., Barnham, K.J., Duce, J.A., and Cherny, R.A. (2016). Amyloid-beta peptide Abeta3pe-42 induces lipid peroxidation, membrane permeabilization, and calcium influx in neurons. J. Biol. Chem. *291*, 6134–6145.

Korbel, J.O., Tirosh-Wagner, T., Urban, A.E., Chen, X.N., Kasowski, M., Dai, L., Grubert, F., Erdman, C., Gao, M.C., Lange, K., et al. (2009). The genetic architecture of Down syndrome phenotypes revealed by high-resolution analysis of human segmental trisomies. Proc. Natl. Acad. Sci. USA *106*, 12031–12036.

Manterola, L., Hernando-Rodriguez, M., Ruiz, A., Apraiz, A., Arrizabalaga, O., Vellon, L., Alberdi, E., Cavaliere, F., Lacerda, H.M., Jimenez, S., et al. (2013). 1-42 beta-amyloid peptide requires PDK1/nPKC/Rac 1 pathway to induce neuronal death. Transl. Psychiatry *3*, e219.

Maulik, M., Peake, K., Chung, J., Wang, Y., Vance, J.E., and Kar, S. (2015). APP overexpression in the absence of NPC1 exacerbates metabolism of amyloidogenic proteins of Alzheimer's disease. Hum. Mol. Genet. *24*, 7132–7150.

Moore, S., Evans, L.D., Andersson, T., Portelius, E., Smith, J., Dias, T.B., Saurat, N., McGlade, A., Kirwan, P., Blennow, K., et al. (2015). APP metabolism regulates tau proteostasis in human cerebral cortex neurons. Cell Rep. *11*, 689–696.

Muller, T., Concannon, C.G., Ward, M.W., Walsh, C.M., Tirniceriu, A.L., Tribl, F., Kogel, D., Prehn, J.H., and Egensperger, R. (2007). Modulation of gene expression and cytoskeletal dynamics by the amyloid precursor protein intracellular domain (AICD). Mol. Biol. Cell *18*, 201–210.

Murray, A., Letourneau, A., Canzonetta, C., Stathaki, E., Gimelli, S., Sloan-Bena, F., Abrehart, R., Goh, P., Lim, S., Baldo, C., et al. (2015). Brief report: isogenic induced pluripotent stem cell lines from an adult with mosaic Down syndrome model accelerated neuronal ageing and neurodegeneration. Stem Cells *33*, 2077–2084.

Ovchinnikov, D.A., Hidalgo, A., Yang, S.K., Zhang, X., Hudson, J., Mazzone, S.B., Chen, C., Cooper-White, J.J., and Wolvetang, E.J. (2015a). Isolation of contractile cardiomyocytes from human pluripotent stem-cell-derived cardiomyogenic cultures using a human NCX1-EGFP reporter. Stem Cell Dev. *24*, 11–20.

Ovchinnikov, D.A., Sun, J., and Wolvetang, E.J. (2015b). Generation of footprint-free induced pluripotent stem cells from human fibroblasts using episomal plasmid vectors. Methods Mol. Biol. *1330*, 37–45.

Oyama, F., Cairns, N.J., Shimada, H., Oyama, R., Titani, K., and Ihara, Y. (1994). Down's syndrome: up-regulation of beta-amyloid protein precursor and tau mRNAs and their defective coordination. J. Neurochem. *62*, 1062–1066.

Ritchie, M.E., Phipson, B., Wu, D., Hu, Y., Law, C.W., Shi, W., and Smyth, G.K. (2015). limma powers differential expression analyses for RNA-sequencing and microarray studies. Nucleic Acids Res. *43*, e47.

Rovelet-Lecrux, A., Frebourg, T., Tuominen, H., Majamaa, K., Campion, D., and Remes, A.M. (2007). APP locus duplication in a Finnish family with dementia and intracerebral haemorrhage. J. Neurol. Neurosurg. Psychiatry *78*, 1158–1159.

Saito, T., Matsuba, Y., Mihira, N., Takano, J., Nilsson, P., Itohara, S., Iwata, N., and Saido, T.C. (2014). Single app knock-in mouse models of Alzheimer's disease. Nat. Neurosci. *17*, 661–663.

Sherman, S.L., Allen, E.G., Bean, L.H., and Freeman, S.B. (2007). Epidemiology of Down syndrome. Ment. Retard. Dev. Disabil. Res. Rev. *13*, 221–227.

Shi, Y., Kirwan, P., Smith, J., MacLean, G., Orkin, S.H., and Livesey, F.J. (2012). A human stem cell model of early Alzheimer's disease pathology in Down syndrome. Sci. Transl. Med. *4*, 124ra129.

Simon, D., Garcia-Garcia, E., Royo, F., Falcon-Perez, J.M., and Avila, J. (2012). Proteostasis of tau. Tau overexpression results in its secretion via membrane vesicles. FEBS Lett. *586*, 47–54.

Sleegers, K., Brouwers, N., Gijselinck, I., Theuns, J., Goossens, D., Wauters, J., Del-Favero, J., Cruts, M., van Duijn, C.M., and Van Broeckhoven, C. (2006). APP duplication is sufficient to cause early onset Alzheimer's dementia with cerebral amyloid angiopathy. Brain *129*, 2977–2983.

Spires-Jones, T.L., and Hyman, B.T. (2014). The intersection of amyloid beta and tau at synapses in Alzheimer's disease. Neuron *82*, 756–771.

Sun, X., He, G., and Song, W. (2006). BACE2, as a novel APP theta-secretase, is not responsible for the pathogenesis of Alzheimer's disease in Down syndrome. FASEB J. *20*, 1369–1376.

Trazzi, S., Fuchs, C., Valli, E., Perini, G., Bartesaghi, R., and Ciani, E. (2013). The amyloid precursor protein (APP) triplicated gene impairs neuronal precursor differentiation and neurite development through two different domains in the Ts65Dn mouse model for Down syndrome. J. Biol. Chem. *288*, 20817–20829.

Vemuri, P., Wiste, H.J., Weigand, S.D., Knopman, D.S., Shaw, L.M., Trojanowski, J.Q., Aisen, P.S., Weiner, M., Petersen, R.C., Jack, C.R., Jr., et al. (2010). Effect of apolipoprotein E on biomarkers of amyloid load and neuronal pathology in Alzheimer disease. Ann. Neurol. *67*, 308–316.

Wu, B.K., Yuan, R.Y., Lien, H.W., Hung, C.C., Hwang, P.P., Chen, R.P., Chang, C.C., Liao, Y.F., and Huang, C.J. (2016). Multiple signaling factors and drugs alleviate neuronal death induced by expression of human and zebrafish tau proteins in vivo. J. Biomed. Sci. *23*, 25.

Young-Pearse, T.L., Chen, A.C., Chang, R., Marquez, C., and Selkoe, D.J. (2008). Secreted APP regulates the function of full-length APP in neurite outgrowth through interaction with integrin beta1. Neural Dev. *3*, 15.

Stem Cell Reports, Volume *11*

Supplemental Information

The Impact of APP on Alzheimer-like Pathogenesis and Gene Expression in Down Syndrome iPSC-Derived Neurons

Dmitry A. Ovchinnikov, Othmar Korn, Isaac Virshup, Christine A. Wells, and Ernst J. Wolvetang

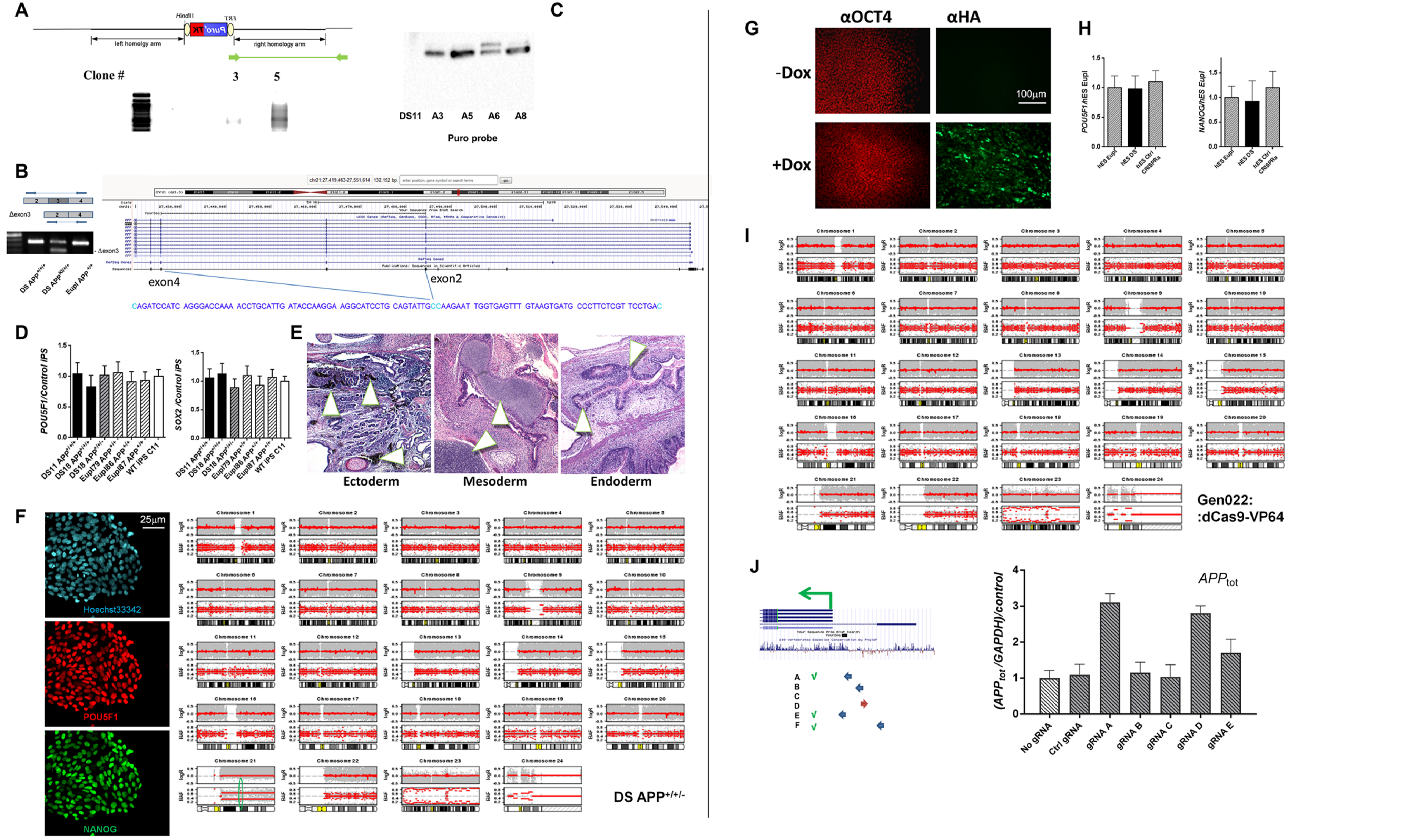

Supplemental Figure S1. (related to Figure 1) Validation of the clonal CRISPR-edited DS iPSC and CRISPRa hESC lines. A. Targeted allele-specific right homology arm-spanning PCR, with primers residing outside of right homology arm and within the selection cassette. **B.** Sequence and alignment of the amplicon of mRNA spliced in APP-targeted clone across the exon 3. **C.** Southern blot of the parental DS iPSC line and Puromycin-resistant clones using a Puromycin cassette-specific probe. **D.** qPCR validation of pluripotency marker expression in the isogenic iPSC cohort. **E.** H&E staining of the teratoma sections illustrating differentiation into the 3 germ layers (ectoderm, mesoderm, endoderm). **F.** Immunofluorescent detection of pluripotency markers POU5F1/OCT4 and NANOG in the DS $APP^{+/+/-}$ iPSC clone. Right panel; SNP analysis gDNA DS $APP^{+/+/-}$. **G.** Immunofluorescence staining of the Gen22::TRE-dCas9-VP64 line with anti-OCT3/4 and anti-HA (to detect HA-dCas9-VP64), in the absence or presence of doxycycline. **H.** qPCR analysis of pluripotency-associated gene expression in Gen21, Gen22, and Gen22::TRE-dCas9-VP64 lines **I.** SNP analysis of Gen22::TRE-dCas9-VP64 CRISPRa line. **J.** Validation of the 5 gRNAs targeting proximal to the main promoter region of the *APP* gene for their ability to mediate activation using a CRISPRa approach in 293FT cells. Values are shown as mean + SEM. N=3, n=2 for **D, H.**

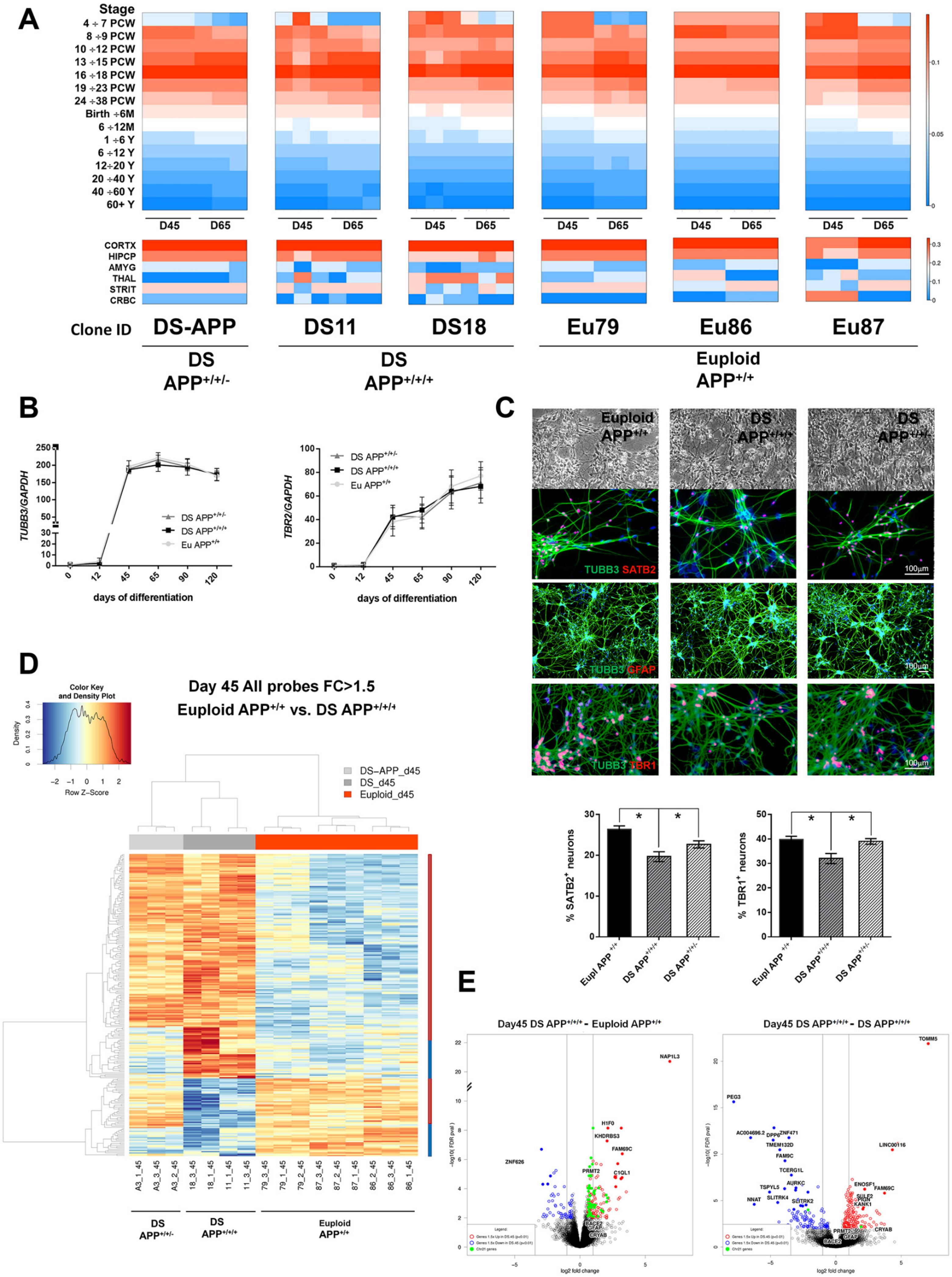
A
Stage
4 ÷ 7 PCW
8 ÷9 PCW
10 ÷12 PCW
13 ÷15 PCW
16 ÷18 PCW
19 ÷23 PCW
24 ÷38 PCW
Birth ÷6M
6 ÷12M
1 ÷6 Y
6 ÷12 Y
12÷20 Y
20 ÷40 Y
40 ÷60 Y
60+ Y
D45 D65
CORTX
HIPCP
AMYG
THAL
STRIT
CRBC
Clone ID
DS-APP
DS11
DS18
Eu79
Eu86
Eu87
DS APP+/+/-
DS APP+/+/+
Euploid APP+/+
B
TUBB3/GAPDH
DS APP+/+/-
DS APP+/+/+
Eu APP+/+
days of differentiation
TBR2/GAPDH
C
Euploid APP+/+
DS APP+/+/+
DS APP+/+/-
TUBB3 SATB2
TUBB3 GFAP
TUBB3 TBR1
100µm
% SATB2+ neurons
% TBR1+ neurons
Eupl APP+/+
DS APP+/+/+
DS APP+/+/-
D
Color Key and Density Plot
Density
Row Z-Score
Day 45 All probes FC>1.5
Euploid APP+/+ vs. DS APP+/+/+
DS−APP_d45
DS_d45
Euploid_d45
A3_1_45
A3_3_45
A3_2_45
18_3_45
18_1_45
11_1_45
11_3_45
79_3_45
79_1_45
79_2_45
87_3_45
87_2_45
87_1_45
86_2_45
86_3_45
86_1_45
DS APP+/+/-
DS APP+/+/+
Euploid APP+/+
E
Day45 DS APP+/+/+ - Euploid APP+/+
Day45 DS APP+/+/- - DS APP+/+/+
NAP1L3
H1F0
KHDRBS3
FAM69C
C1QL1
PRMT2
ZNF626
BACE2
GFAP
CRYAB
TOMM5
PEG3
AC004696.2
DPP6
ZNF471
TMEM132D
FAM9C
LINC00116
TCERG1L
AURKC
TSPYL5
SLITRK4
SLITRK2
NNAT
ENOSF1
SULF2
PRDM
KANK1
FAM69C
PRMT2
GFAP
BACE2
CRYAB
-log10(FDR pval)
log2 fold change

Supplemental Figure S2 (related to Figure 2). Transcriptomic profiling and characterization of the cortical neurogenic cultures A. Temporal and spatial identity assignment of the day 65 neuronal differentiation samples (5 clones: APP copy number-normalized DS11, 2 Hsa21-trisomic DS and 3 euploid clones) using RNA expression data from Illumina HT12 microarray and an online resource CoNTExT (https://context.semel.ucla.edu/). Scale bars reflecting predicted probabilities of temporal (top) or spatial (bottom) class memberships are shown on the right. **B.** Time course qPCR analysis of pan-neuronal marker *TUBB3* and intermediate neuronal progenitor marker *TBR2* during the cortical neuronal differentiation of the isogenic iPSC cohort **C.** Representative phase-contrast (top row) and epifluorescence (rows 2-4) images of neuronal cultures of all 3 genotypes at day 90 (GFAP staining in 3rd row at day 55) indicates comparable abundance of cortical layer markers SATB2 and TBR1 (quantification relative to TUBB3^{+} cells shown in **C'**). **D.** Heatmap based on hierarchical clustering of all significantly differentially-expressed probes (|FC|>1.5, P_{adj} <0.01) at day 45. **E.** Volcano plots for probes differentially expressed in DS vs euploid (left) and DS vs. APP copy number-normalized DS neuronal cultures (right) at day 45. Hsa21 genes shown in bright-green, genes down- or up-regulated >1.5 fold shown in blue and red, respectively. Negative decimal logarithm of the false discovery rate probability plotted on Y axis, log_2 of the fold change plotted on X with thin line at 0.5/2 FC values. Values are shown as mean ± SD (**B, C'**).

`

Supplemental Figure S3 (related to Figures 3, 4). A. Western blot showing upregulation of APP levels is retained in day 90 DS neurones, and fully offset by normalisation of the APP copy number. Quantification relative to beta-Actin. **B.** Quantification of Aβ42 and Aβ42/40 ratios in medium of day 90 DS and sibling euploid hESC-derived neurons (as measured by peptide-specific specific ELISA). **C.** Quantification of Aβ40 petide levels in day 90 culture medium from DS, DS copynumber normalised and euploid neurons. **D.** Effects of APP level modulation on *BACE2* (3 left panels) and *BACE1* transcript levels (qPCR) at indicated timepoints. **E.** Modulation of the Aβ42/40 ratio by the BACE inhibitor Verubicestat (Verub., used at 1 nM in DMSO) based on measurements from the medium of 90 day-old neurons of all 3 DS patient-derived genotypes described in this study. **F.** Effect of Verubicestat and the γ-secretase inhibitor DAPT on absolute levels of Aβ42 peptide secreted by day 90 DS neurons. **G**. Example of a western blot for pyroglutamate(pE3)-Ab in isogenic DS neurons quantified in Figure 3F. **H**. Western blot and its quantification showing pE3-pyroglutamate levels relative to TUBB3 in APP-overexpressing day 90 Gen22::TRE-dCas9-VP64 derived neurons bearing the indicated gRNAs. **I.** An example of TUBB3/Hoechst33342 - stained neuronal cultures at day 90 (top) used for automated neurite length calculation (see Supplemental information for details). **J.** An isogenic cohort of 2 DS trisomic clones, APP copy number-normalized clone and 3 euploid clones (at day 65 of neuronal differentiation); **K.** Gen22::TRE-dCas9-VP64 derived neurons (day 90) bearing the indicated APP promoter targeting gRNAs. Values shown are means ±SEM, N=3, n=2. * p<0.05, ** p<0.01, *** p<0.001, # ns. Mean values and SEM shown on graphs.

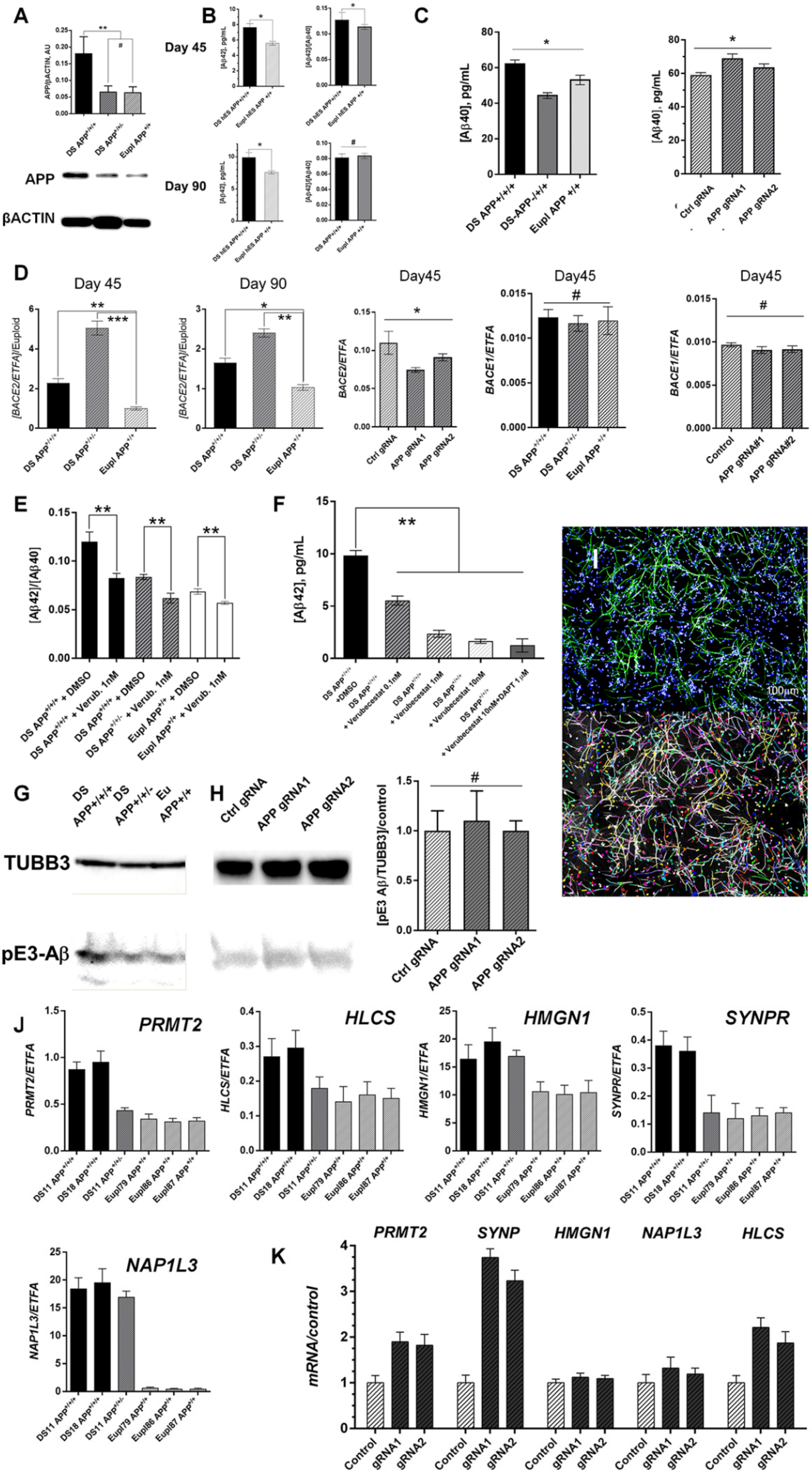

A
APP/βACTIN, AU
DS APP+/+/+
DS APP+/+/-
Eupl APP+/+
APP
βACTIN
B
Day 45
Day 90
[Aβ42], pg/mL
[Aβ42]/[Aβ40]
DS hES APP+/+/+
Eupl hES APP +/+
C
[Aβ40], pg/mL
DS APP+/+/+
DS-APP-/+/+
Eupl APP +/+
Ctrl gRNA
APP gRNA1
APP gRNA2
D
Day 45
Day 90
Day45
[BACE2/ETFA]/Euploid
BACE2/ETFA
BACE1/ETFA
Control
APP gRNA#1
APP gRNA#2
E
[Aβ42]/[Aβ40]
DS APP+/+/+ + DMSO
DS APP+/+/+ + Verub. 1nM
DS APP+/+/- + DMSO
DS APP+/+/- + Verub. 1nM
Eupl APP+/+ + DMSO
Eupl APP+/+ + Verub. 1nM
F
[Aβ42], pg/mL
DS APP+/+/+ +DMSO
DS APP+/+/+ + Verubecestat 0.1nM
DS APP+/+/+ + Verubecestat 1nM
DS APP+/+/+ + Verubecestat 10nM
DS APP+/+/+ + Verubecestat 10nM+DAPT 1 μM
100μm
G
DS APP+/+/+
DS APP+/+/-
Eu APP+/+
H
Ctrl gRNA
APP gRNA1
APP gRNA2
TUBB3
pE3-Aβ
[pE3 Aβ/TUBB3]/control
J
PRMT2
HLCS
HMGN1
SYNPR
NAP1L3
PRMT2/ETFA
HLCS/ETFA
HMGN1/ETFA
SYNPR/ETFA
NAP1L3/ETFA
DS11 APP+/+/+
DS18 APP+/+/+
DS11 APP+/+/-
Eupl79 APP+/+
Eupl86 APP+/+
Eupl87 APP+/+
K
PRMT2
SYNP
HMGN1
NAP1L3
HLCS
mRNA/control
Control
gRNA1
gRNA2

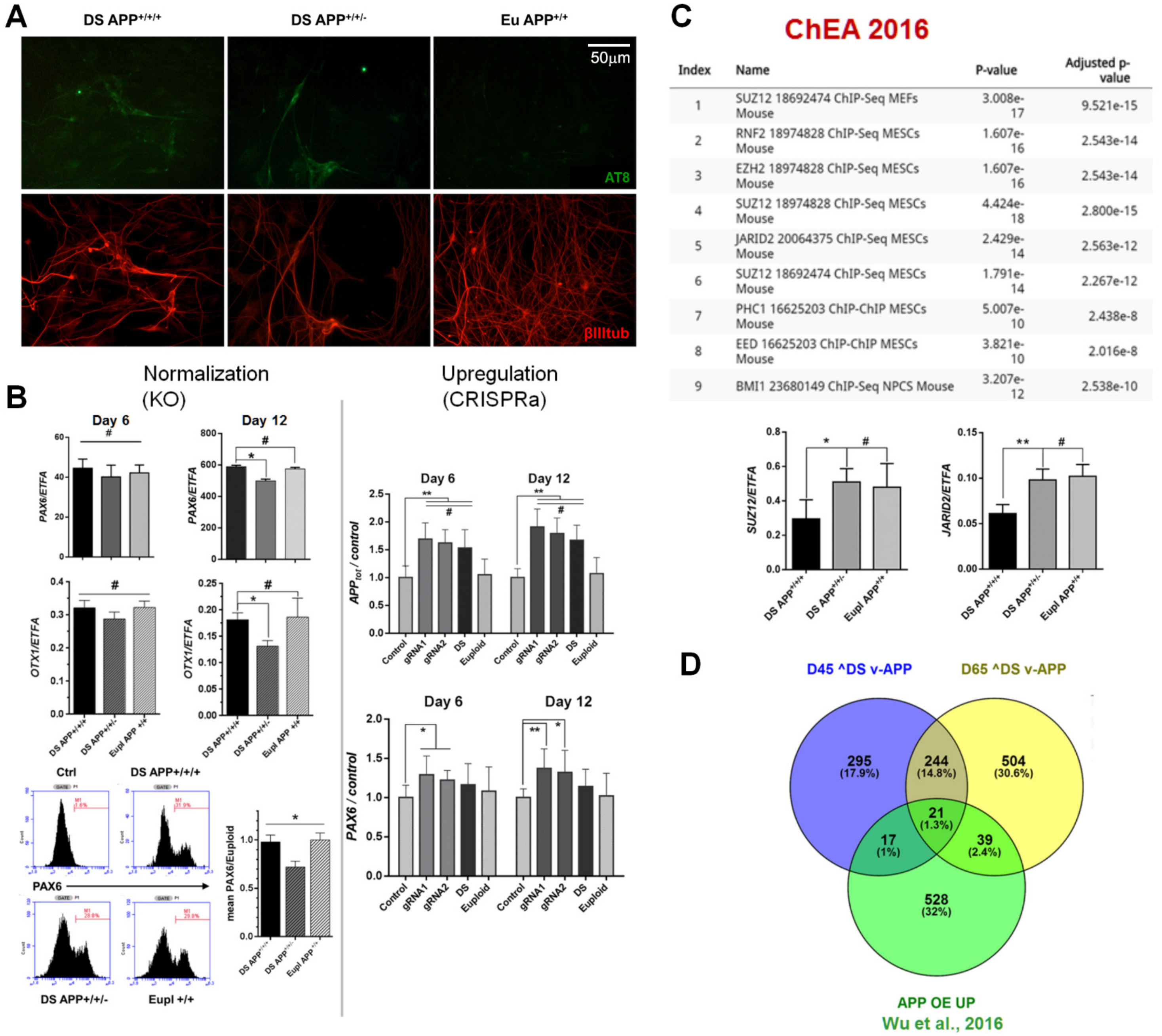

Index	Name	P-value	Adjusted p-value
1	SUZ12 18692474 ChIP-Seq MEFs Mouse	3.008e-17	9.521e-15
2	RNF2 18974828 ChIP-Seq MESCs Mouse	1.607e-16	2.543e-14
3	EZH2 18974828 ChIP-Seq MESCs Mouse	1.607e-16	2.543e-14
4	SUZ12 18974828 ChIP-Seq MESCs Mouse	4.424e-18	2.800e-15
5	JARID2 20064375 ChIP-Seq MESCs Mouse	2.429e-14	2.563e-12
6	SUZ12 18692474 ChIP-Seq MESCs Mouse	1.791e-14	2.267e-12
7	PHC1 16625203 ChIP-ChIP MESCs Mouse	5.007e-10	2.438e-8
8	EED 16625203 ChIP-ChIP MESCs Mouse	3.821e-10	2.016e-8
9	BMI1 23680149 ChIP-Seq NPCS Mouse	3.207e-12	2.538e-10

Supplemental Figure S4 (Related to Figures 4, 2). Interpretation and comparison of mRNA expression profiling results from the isogenic cohort of DS (Hsa21-trisomic), euploid and APP copy number-normalized DS neurons. A. Detection of hyperphosphorylated tau with AT8 anti-phospho-Tau antibody (Day 120 neuronal culture). **B.** Venn diagram showing overlap of DEG lists of transcripts upregulated in DS vs euploid and downregulated in DS-APP vs DS at days 45 (blue circle) and 65 (yellow circle) with APP overexpression-driven genes (green) from Wu et al. **C. S**ignificance of an overlap between 265 (244+21) APP copy number dependent differentially expressed genes with ChIP Enrichment Analysis (ChEA 2016), qPCR validation of APP copy number-dependent expression of the PRC2 components, *SUZ12* and *JARID2,* in day 65 neuronal cultures **D.** Venn diagrams built using a free online resource http://bioinfogp.cnb.csic.es/tools/venny/. * p<0.05, ** p<0.01, # ns. N=3, n=2 for qPCR Values shown are means±SEM.

Table S1 (Related to Figure 2): Analysis of the pathway alignment of APP-dependent or dynamically-regulated gene sets (using ToppGene online tool); validation of the APP synexpression groups using the Enrichr integrated gene list analysis online tool.

APP-regulated genes at day 65 (shown in Venn diagram in Figure 2B)

DAPL1, LOC404266, CRYAB, ALDH1A3, SCRG1, GFAP, RBP1, CFI, C18ORF51, MMP7, DACT2, HSD17B2, C1QL1, SYNPR, RDH10, DCT, HOPX, HOXB6, SIX6, NRIP3, A2M, SPP1, LEFTY2, METRN, PMP2, SCUBE2, HS.204481, CA14, IGFBP5, KAL1, CHSY3, C21ORF62, MARCH3, LMO2, RAB31, SMOC1, TAGLN2, PRSS23, LOC100133609, HS.569104, NFIX, SYT4, TRAF3IP2, F3, CPNE4, SLC44A3, TNC, DKK3, PPP1R1B, RBM20, RELN, BHLHE22, BAALC, NECAB2, CPS1, RHPN2, EBF3, SERPINB6, CRYM, RASD1, ATP1B2, MGP, SULF2, LPAR1, PCDH17, GLIS3, HTRA1, KANK1, NKAIN4, GPC3, HS.531457, NHLH2, RGS20, PODXL, LYN

**Gene expression signature/ID**	Gene overlap, UP @D45	Gene overlap, UP @D65
**Down syndrome (umls:C0013080)**	IL10RB, C21orf33, PIGP, DONSON, PDE9A, GART, WRB, DSCR3, PFKL, APP, TTC3, USP16, PDXK, IFNAR1, IFNGR2, SYNJ1, PSMG1, N6AMT1, CYYR1, PKNOX1, CSTB, PRMT2, CRYZL1, TMEM50B, SON	MRPL39, IL10RB, U2AF1, C21orf33, ATP5O, WRB, CSTB, HLCS, PRMT2, CRYZL1, BACE2, TMEM50B, GABPA, MX1, IFNAR2
**Alzheimer's Disease (umls:C0002395)**	PAG1, RELN, MAOA, SERPINF1, GART, CPLX1, GSTO2, EOMES, C1QL1, CDK5R1, SYNJ1, KCND2, NFIX, SYN1, SYN2, BACE2, HS3ST1, HOMER2, CA2, CAMK2A, NTS, KHDRBS2, GRIN1, APP, SLC1A2, IGFBP5, SCG3, TSPAN7, ELAVL4, SNAP25, CTNNA2, SNCA, MYO16	

**Dynamically-regulated pathways D65-D45**	Euploid (3 clones) D65-D45	
**trans-synaptic signalling (GO:0099537)**	DLGAP2, PDYN, TMOD2, MGLL, MEF2C, NRXN1, SYT13, PMCH, HTR2C, PPFIA4, PPFIA2, ICA1, BCAN, APOE, GABBR2, SLC1A1, SLC1A2, PRKCE, SNCB, ATP2B2, GABRA2, GABRG2, GLRB, TSPO, SLC17A7, DLG2, GRIN3B, CA2, HPCA, CAMK2A, CAMK2B, NTSR1, PCDHB4, GRIA2, GRIA3, GRIN1, SYT7, CCKBR, ZNF225, SEZ6, EXOC4	
**, Neuronal system (#1268763)**		DLGAP2, GNG8, MAOA, NRXN1, PPFIA4, PPFIA2, GABBR2, SLC1A1, SLC1A2, PRKACB, PRKCB, GABRA2, GABRG2, KCNA5, KCNF1, KCNJ4, KCNK1, SLC17A7, DLG2, CAMK2A, CAMK2B, GRIA2, GRIA3, GRIN1, SYT7, KCNH3

Table S2 (Related to Figure 2): Ingenuity (Qiagen) prediction of the pathways most-affected by the Hsa21 trisomy from DS vs Euploid comparison (D45)

Categories	Diseases or Functions Annotation	p-Value	Predicted Activation State	Activation z-score	№ of Molecules	Molecules
Neurological Disease	**neurological signs**	8.7E-08	⌂	2.184	43	ADCY1,APP,BHLHE22,CA2,CAMK2A,CAMK2B,CDK5R1,CHRM3,CNR1,CRYAB,CTGF,CX3CL1,DAAM2,DIO2,DIRAS2,DKK3,DMRT3,DONSON,ELAVL2,GPRC5C,GRIA2,GRIN1,GYPC,HSPA1A/HSPA1B,IER3,LMCD1,MAL2,NDRG1,NTF3,PCDH7,PLCB1,PPP1R1B,SALL4,SCN3B,SLC1A2,SMAD6,SNAP25,SNCA,SRPX,SYNJ1,SYNPR,TTR,VAMP2,ADCY1,APP,ATP1A2,BCHE,CA14,CA2,CA4,CCL2,CDK5R1,CHRM3,CNR1,CRH,GABBR2,
Neurological Disease	**cognitive impairment**	3.25E-06	⌂	2.173	29	GRIN1,MAF,MAOA,NTF3,PLAT,PMM2,PTGER4,SCN3B,SLC1A2,SNAP25,SNCA,SYNJ1,TSPAN7,TTR,TUBB2A,VAMP2
Neurological Disease, Organismal Injury and Abnormalities	**abnormality of cerebral cortex**	2.05E-09	⌂	2.05	25	ADCY1,APP,ATF3,BRINP1,CDK5R1,CNR1,CTNNA2,CTSV,CX3CL1,GAP43,GPR37,GRIA2,GRIN1,HSD17B2,NFIX,OTX1,OTX2,PLAT,POU3F2,SLC1A2,SNAP25,SNCA,SPP1,SYN1,VAMP2

Table S3 (Related to Figure 2): Analysis of the pathways influenced by APP copy number in DS neurons using the Reactome and KEGG DEG list pathway alignment-analysing open access bioinformatic tools

APP-dependent upregulated in DS in day 45 neurons

ID	Description	GeneRatio	BgRatio	pvalue	p.adjust	qvalue	GeneID
72163	mRNA Splicing - Major Pathway	25/603	115/6749	2.05E-05	0.008390092	0.007225009	HNRNPA1/HNRNPK/SF3A3/HNRNPA3/U2AF1/MAGOH/SF3B6/SNRPB/HSPA8/CPSF1/CSTF3/DHX9/SRSF2/SNU13/PHF5A/ALYREF/SNRPA1/SNRPA/HNRNPUL1/HNRNPM/HNRNPR/HNRNPD/POLR2C/PRPF19/PABPN1
72172	mRNA Splicing	25/603	115/6749	2.05E-05	0.008390092	0.007225009	HNRNPA1/HNRNPK/SF3A3/HNRNPA3/U2AF1/MAGOH/SF3B6/SNRPB/HSPA8/CPSF1/CSTF3/DHX9/SRSF2/SNU13/PHF5A/ALYREF/SNRPA1/SNRPA/HNRNPUL1/HNRNPM/HNRNPR/HNRNPD/POLR2C/PRPF19/PABPN1
2262752	Cellular responses to stress	43/603	256/6749	3.10E-05	0.008390092	0.007225009	CAT/HDAC6/NUP85/H3F3B/MAPKAPK3/RING1/UBE2D3/FKBP4/YWHAE/TXNRD2/VCP/PRDX5/HSPA8/RPA1/TNRC6B/IGFBP7/CBX4/HSPA1B/MAPK3/NUP107/TERF2/UBA52/MAPK8/UBE2D2/CAMK2A/UBB/CAMK2B/SOD1/PRDX3/GPX8/SEH1L/ID1
72203	Processing of Capped Intron-Containing Pre-mRNA	25/603	119/6749	3.79E-05	0.008390092	0.007225009	HNRNPA1/HNRNPK/SF3A3/HNRNPA3/U2AF1/MAGOH/SF3B6/SNRPB/HSPA8/CPSF1/CSTF3/DHX9/SRSF2/SNU13/PHF5A/ALYREF/SNRPA1/SNRPA/HNRNPUL1/HNRNPM/HNRNPR/HNRNPD/POLR2C/PRPF19/PABPN1
3560783	Defective B4GALT7 causes EDS, progeroid type	8/603	19/6749	0.000120295	0.017743474	0.015279543	CSPG5/BCAN/GPC6/SDC2/BGN/NCAN/GPC4/SDC1
3560801	Defective B3GAT3 causes JDSSDHD	8/603	19/6749	0.000120295	0.017743474	0.015279543	CSPG5/BCAN/GPC6/SDC2/BGN/NCAN/GPC4/SDC1
1971475	A tetrasaccharide linker sequence is required for GAG synthesis	9/603	25/6749	0.000188604	0.023844918	0.020533715	CSPG5/BCAN/GPC6/SDC2/BGN/NCAN/GPC4/SDC1/GXYLT1

APP-dependent upregulated in DS day 65 neurons

ID	Description	GeneRatio	BgRatio	pvalue	p.adjust	qvalue	GeneID
422475	Axon guidance	58/753	292/6749	6.15E-06	0.005696398	0.005484652	EPHB4/CRMP1/ARHGEF7/ST8SIA2/LAMB1/MYH10/CDC42/LAMA1/VASP/DNM1/AP2B1/YES1/DOCK1/KRAS/ABLIM1/SDC2/CDK5R1/TIAM1/GRIN1/DPYSL2/NCAM1/ERBB2/LAMC1/
112315	Transmission across Chemical Synapses	42/753	194/6749	1.43E-05	0.006623393	0.00637719	GNG5/PPFIA2/STXBP1/SLC6A1/CAMK2B/GABRB3/GABRA2/GABRA5/GABRG2/GNG2/PRKCB/SLC1A1/GABRA3/AP2B1/GNGT1/LIN7B/CPLX1/GNB1/CALM3/GRIN1/SNAP25/GAD1/VA
1566948	Elastic fibre formation	14/753	39/6749	4.27E-05	0.013187281	0.012697086	ITGB5/MFAP4/BMP7/FBN2/LTBP3/BMP2/FBLN5/FBLN1/MFAP2/LOXL1/ITGB1/ELN/ITGA5/FBLN2
888590	GABA synthesis, release, reuptake and degradation	9/753	19/6749	8.37E-05	0.019381838	0.018661381	STXBP1/SLC6A1/CPLX1/SNAP25/GAD1/VAMP2/STX1A/GAD2/SLC32A1
977441	GABA A receptor activation	7/753	13/6749	0.00019606	0.025658115	0.024704357	GABRB3/GABRA2/GABRA5/GABRG2/GABRA3/GABRB1/GABRA1
1266738	Developmental Biology	73/753	438/6749	0.000213329	0.025658115	0.024704357	SMAD4/MED30/EPHB4/CRMP1/ARHGEF7/MED25/ST8SIA2/LAMB1/MYH10/CDC42/LAMA1/MED16/VASP/DNM1/AP2B1/YES1/DOCK1/KRAS/ABLIM1/SDC2/CDK5R1/TIAM1/KLF4/I
977443	GABA receptor activation	16/753	55/6749	0.000220293	0.025658115	0.024704357	GNG5/GABRB3/GABRA2/GABRA5/GABRG2/GNG2/GABRA3/GNGT1/GNB1/GABBR2/ADCY1/GNG12/KCNJ2/GABRB1/GABRA1/GABBR1
2129379	Molecules associated with elastic fibres	12/753	35/6749	0.000249015	0.025658115	0.024704357	ITGB5/MFAP4/BMP7/FBN2/LTBP3/BMP2/FBLN5/FBLN1/MFAP2/ITGB1/ELN/FBLN2
112310	Neurotransmitter Release Cycle	14/753	45/6749	0.000249377	0.025658115	0.024704357	PPFIA2/STXBP1/SLC6A1/SLC1A1/LIN7B/CPLX1/SNAP25/GAD1/VAMP2/SLC1A3/STX1A/MAOA/GAD2/SLC32A1

Table S4 (related to Figure S4): Gene memberships of the overlaps in APP-regulated gene sets identified using Venn diagrams depicted in Figure S4D

**244 genes Upregulated in DS vs Euploid and DOWNregulated in DS-APP vs DS at both D45 and D65**	TOMM5, HOXB8, CRYAB, HOXB5, NLRP2, LOC205251, SULF2, LMO2, DAPL1, NFIX, ALDH1A3, CFI, PODXL, SYNPR, A2M, HOXA5, LOC404266, HS.19193, C1QL1, RAB31, RAX, ATOH8, BHLHE22, HOXC4, PRSS23, POU4F2, ITGB5, CYP1B1, HOXC6, PRPH, DACT2, HOPX, CHX10, ALPK2, HS.71947, ENOSF1, TNC, CLDN1, SIX6, COLEC12, EBF3, SERPINF1, C18ORF51, CPS1, WNT7B, GJA1, TXNL1, CXXC5, TYMS, CDKN1A, HOXB7, MALT1, TGIF1, ZNF680, TPBG, GLIS3, FABP7, ZNF521, KAL1, DUSP6, SMOC1, PARD6G, CYB5A, PIGN, CPNE4, KANK1, C1QTNF5, PPP4R1, ME2, MDK, RSPO3, SPARCL1, HOXB6, ADAMTS1, TUBB6, RDH10, NDUFV2, DKK3, ATP1B2, HS3ST3A1, ABLIM1, C3ORF58, PLS3, BCL2, RBMS1, NME5, CA14, ATOH7, RBM20, LOC643911, CHSY3, HOXC8, SERPINB6, PRODH, FVT1, NDC80, RASD1, DTNA, RELN, HS.531457, ZIC3, PLCE1, PPP1R1B, HS.552087, SPP1, AFG3L2, C12ORF31, PSMG2, IMPA2, MMP7, LOC100130502, LPAR1, PLTP, CXCR4, MEX3C, HOMER2, LOC285943, LOXL1, GDF10, C4ORF31, KLHDC8B, DCT, ZNF503, MRLC2, NHLH2, MYL12A, MARCH3, CDH6, EFCBP1, HOXB3, HSD17B2, DCN, KCNJ13, TPPP3, OTX2, FLNC, MAPK4, CCND1, CHMP1B, C18ORF1, C21ORF34, RHPN2, TRPM3, FAR2, SULF1, TRAF3IP2, CASP6, FHL2, FAM167A, FBN2, HS.553310, SERPING1, SYT4, LPIN2, ITGA2, TNFRSF11B, HS.574278, SEC11C, VWC2, PQLC1, ACOX2, HS.582985, ZNF248, LOC100133609, F3, GRM8, C1ORF24, RARB, HOXA2, C6ORF165, PDLIM1, LOC730254, PON2, C8ORF47, COL18A1, ZNF266, SLC39A4, HS.278285, CLSTN2, HS.569104, SCUBE2, TAGLN2, NRIP3, KIAA1468, C8ORF13, HS.552008, PLEKHA4, SEH1L, C21ORF62, SOCS3, NECAB2, SLC44A3, CHST3, COL4A1, HS.204481, ARL4C, FNDC5, ZNF532, CD44, TMEM132B, ID1, STX8, OSBPL1A, BAALC, SH3D19, RHOU, LAPTM4B, LOC154860, IL13RA1, SH3BP4, HS.143937, FEZ2, C18ORF22, EMP1, NPPB, C18ORF10, SCRG1, TGFBI, HEPH, ADAMTS3, IFITM2, GREM1, RNF182, CD9, KITLG, EPB41L3, ADD3, HOXB2, ABCA1, OTP, FBLN2, HS.61208, LOC401052, DYNLRB2, ANKRD12, GLIPR2, FOXJ1, FOLR1, SESN1, SDSL, AGTRAP, BMP7, LOC100134265, TSHZ,

**21 genes upregulated by APP in Wu et al. dataset and regulated by APP copy number at D45&D65**	RBP1, HTRA1, APCDD1, IGFBP5, ID3, BMP2, LAMA1, ARL4A, SILV, TWSG1, PMAIP1, SEMA6A, PTPRM, PCDH17, PMP22, VIM, GPC3, ACADVL, GPR98, LOC400657, MSRA

Alignment of the transcriptomic changes with gene sets regulated by components of the Polycomb Repressor Complex 2

Gene lists for ChEA 2016 analysis (Enrichr)	Test DEG list	P_{adj} value	Pathway overlap membership
SUZ12_ 18692474	See above	0.0000000000001	**55/1135:** CLSTN2;FHL2;RAX;PTPRM;OTP;CDH6;SIX6;ADAMTS1;GRM8;CYP1B1;HOXA2;HOXA5;CHX10;SEMA6A;IGFBP5;LMO2;EBF3;C1QL1;GREM1;ALDH1A3;COL4A1;HOXB3;HOXB8;HOXB7;HOXB6;HOXB5;FBN2;HS3ST3A1;NFIX;HTRA1;CXCR4;SCUBE2;NHLH2;RELN;RASD1;PODXL;ATOH8;HOXC4;OTX2;FLNC;HOXC8;HOXC6;ABCA1;GDF10;DTNA;HOMER2;WNT7B;BMP7;POU4F2;BMP2;PPP1R1B;ID1;BCL2;ID3;TGFBI

EZH2<br>18974828	See above	0.000000000000025	58/1302:<br>CLSTN2;RAX;PTPRM;OTP;PRSS23;SIX6;ADAMTS3;ADAMTS1;GRM8;HOXA2;CYP1B1;HOXA5;CHX10;SEMA6A;IGFBP5;LMO2;EBF3;C1QL1;GREM1;AFG3L2;ALDH1A3;COL4A1;HOXB3;HOXB2;HOXB8;HOXB7;HOXB6;HOXB5;FBN2;COLEC12;HS3ST3A1;NFIX;TSHZ1;GLIS3;HTRA1;CXCR4;PCDH17;SCUBE2;NHLH2;RELN;PODXL;ATOH8;GPC3;HOXC4;OTX2;FLNC;HOXC8;HOXC6;GDF10;HOMER2;WNT7B;BMP7;POU4F2;BMP2;PPP1R1B;BCL2;VWC2;ID3
SUZ12<br>18974828	As above	0.0000000000000028	75/1934:<br>CLSTN2;FHL2;RAX;PTPRM;OTP;CLDN1;PRSS23;LOXL1;CDH6;SIX6;ADAMTS3;CCND1;ADAMTS1;SESN1;GRM8;HOXA2;CYP1B1;RSPO3;HOXA5;CHX10;TGIF1;MMP7;SEMA6A;IGFBP5;LMO2;EBF3;C1QL1;GREM1;ALDH1A3;RAB31;COL4A1;HOXB3;HOXB2;HOXB8;HOXB7;CD44;HOXB6;CHST3;HOXB5;FBN2;COLEC12;NFIX;HS3ST3A1;PON2;TSHZ1;HTRA1;GLIS3;CXCR4;PCDH17;SCUBE2;ATOH7;NHLH2;RELN;PODXL;ATOH8;GPC3;HOXC4;NRIP3;OTX2;FLNC;HOXC8;HOXC6;GDF10;HOMER2;WNT7B;TPBG;BMP7;POU4F2;BMP2;PPP1R1B;BCL2;VWC2;PMP22;ID3;VIM
JARID2_<br>20064375	As above	0. 0000000000025	50/1117<br>CLSTN2;RAX;PTPRM;OTP;CDH6;SIX6;ADAMTS1;GRM8;CYP1B1;HOXA2;RSPO3;HOXA5;SEMA6A;IGFBP5;LMO2;EBF3;C1QL1;FNDC5;GREM1;COL4A1;HOXB3;HOXB2;HOXB8;HOXB7;HOXB6;HOXB5;FBN2;NECAB2;HS3ST3A1;NFIX;TSHZ1;GLIS3;HTRA1;CXCR4;PCDH17;SCUBE2;NHLH2;RELN;ATOH8;CHSY3;HOXC8;HOXC6;HOMER2;WNT7B;BMP7;POU4F2;BMP2;PPP1R1B;BCL2;VWC2
EED_<br>16625203	As above	0.00000002	36/830:<br>FBN2;COLEC12;NFIX;FHL2;RAX;CXCR4;OTP;LOXL1;NHLH2;SIX6;RELN;RASD1;ADAMTS1;PODXL;ATOH8;HOXA2;CYP1B1;HOXC4;PRODH;HOXA5;HOXC6;CHX10;GDF10;SEMA6A;IGFBP5;EBF3;BMP7;POU4F2;COL4A1;PMP22;ID3;HOXB3;HOXB2;HOXB8;HOXB7;HOXB6

Supplemental experimental procedures

Targeting the APP locus using CRISPR/Cas9-aided homologous recombination in hiPSCs

For gene targeting, $3x10^6$ pluripotent DS iPS cells were transfected with 2.5µg of targeting vector (Figure 1) together with 1 µg a pre-validated gRNA and Cas9 expressing plasmid (pX459, a gift from Feng Zhang, MIT) using CA-137 protocol in 4D Nucleofector (Lonza, Switzerland) according to manufacturer's instruction and primary cell transfection buffer kit PC3 (Lonza, Switzerland). Induction of *APP* overexpression was performed using aqueous doxycycline hyclate (Sigma-Aldrich, USA) at 1 µg/mL in culture medium unless indicated otherwise. Overexpression of APP by transfection was achieved using either 2 µg pCAX-APP695 plasmid (Addgene plasmid 30137) or co-transfection of 1 µg of SP-dCas9-VPR (Addgene 63798) and 0.5 µg each of validated (data not shown) gRNAs (in constitutive lentiviral vector Addgene 52628) targeting regions upstream of *APP* promoter into matured hiPSC-derived euploid neurons (from the isogenic 6-clone cohort) in a 24-well plate using Lonza neuronal transfection kit (Lonza, Switzerland).

To address the role of the supernumerary copy of the APP gene on development of AD neuropathology in DS we edited one APP allele in a previously characterised footprint-free DS-iPSC line with full trisomy of HSA21 - clone C11DS (Briggs et al., 2012). Exon 3 of the APP gene was replaced with a Puro-TK selection cassette using CRISPR/SpCas9-assisted homology-directed repair (DS $APP^{+/+/-}$ line, Figure 1A). Correct targeting was confirmed using PCRs specific to the correctly targeted allele (Figure 1B, Figure S1A) and Southern blotting (Figure 1C). While deletion of exon 3 still permits low-level splicing between exons 2 and 4 (Figure S1B, mRNA from neuronal cultures on day 65), splicing of the resulting transcript introduces a frameshift and should not lead to the synthesis of a functional APP protein. Indeed, the ~1.5-2-fold increase in APP protein expression observed in neuronally differentiated DS $APP^{+/+/+}$ iPSC was "normalised" to euploid levels in isogenic CRISPR-targeted $APP^{+/+/-}$ DS iPSC-derived neuronal cultures (Figures 1D and 1E), and this was maintained during prolonged neuronal differentiation (up to 90 days tested, Figure S3A). The clonal APP allele-targeted $APP^{+/+/-}$ DS iPSC line exhibits the expected expression of pluripotency markers (Figure S1D), the ability to form representatives of all three germ layers in teratoma assays (Figure S1E), and maintains a stable HSA21-trisomic karyotype without obvious deletions, duplications or insertions as assessed by SNP array analysis (Figure S1F).

Generation of stable hPSCs allowing for CRISPRa-driven upregulation of the APP gene.

To assess the effect of APP overexpression in a euploid (HSA21-disomic) background we modified a euploid human ES cell line Genea022 (Dumevska et al., 2016b) with lentiviral transgenes carrying a doxycycline-inducible artificial transactivator dSpCas9-VP64, permitting comparison with its sibling HSA21-trisomic DS hESC counterpart, Genea021 (Dumevska et al., 2016a). Transduction of the Genea022::TRE-dCas9-VP64 line with a puromycin-selectable lentivirus constitutively expressing one of the pre-validated gRNAs that direct the transcriptional activator upstream of the main *APP* promoter, allows for doxycycline-tunable overexpression of APP protein to levels comparable to DS (Figures 1F and 1G). Induction of the HA-tagged dCas9-VP64, and APP overexpression was confirmed by western blotting using antibodies listed below.

Generation of the cortical neurogenic cultures

Simultaneous inhibition of TGFbeta/Activin and BMP/GDF signalling pathways was used to generate neurogenic cultures (Briggs et al., 2013; Chambers et al., 2009). Briefly, SB431542 at 10µM and dorsomorphin at 5µM were used for inhibition of the abovementioned pathways, respectively, for the first 6 and 12 days of differentiation medium (KOSR gradually exchanged for N2B27 (neurobasal medium supplemented with 2% B27, 1% N2 and 1% GlutaMax , all from LifeTechnologies) in 25% incremental steps on days 4, 6, 8, 10). The neuroepithelial cell layer formed by day 6 was mechanically separated into evenly-sized pieces of ~500 cells/each and transferred in differentiation medium in ultra-low attachment plate for neurosphere formation. After day 12, neurospheres were plated on Matrigel-coated (1/50 dilution of original supplied stock in DMEM/F12 base medium) TC-treated plastic. Growing neurogenic cultures were passaged every ~10 days, and were plated on poly-L-ornithine/human laminin-coated TC plastic, glass coverslips or chamber slides (plastic or glass) 2-6 weeks before assays (unless specified otherwise). Further maturation was achieved by supplementing the differentiation medium with 20ng/mL BDNF, 10ng/mL GDNF, 500nM ascorbic acid and 1mM dibutyl-cyclic AMP.

Microarray analysis: sample and data preparation

Total RNA from triplicates of day 45 and 65 neuronal differentiation cultures[17,71] was prepared using direct lysis on the plate with TriZOL reagent (LifeTechnologies), followed by total RNA isolation using PureLink RNA kit

(Ambion/LifeTech) with on-column DNase treatment (as per manufacturer's instructions). After RNA integrity verification using BioAnalyzer (Agilent Technologies, USA), RIN=10 RNA samples were used for Illumina HT-12 microarray hybridization and an iSCAN analysis (performed by AGRF-Melbourne). The summarised probe profile and control probe files were read into the statistical programming language R (https://www.R-project.org/), and analysed using packages from the Bioconductor project(Huber et al., 2015). Quality was assessed using array QualityMetrics (Kauffmann et al., 2009), and two samples (DS11 and DS18 at day 45) were determined to be outliers and were removed from any subsequent analysis. The samples underwent *normexp* background correction using the negative control probes and quantile normalization using the negative and positive control probes, using the *normexp* function from the Limma package (Ritchie et al., 2015). Any probes not considered to be detected (using a detection p-value of less than 0.01), were removed, resulting in 25,378 probes and 34 samples. A linear model was fitted taking into account the group and the time point (results of this analysis were used for evaluation of spatiotemporal identity of neurogenic samples using CoNTExT and Enrichr analysis of consistently APP-dependent genes). For other analyses, the otherwise unaltered sample probe profile with remaining 34 samples was independently background-corrected and quantile normalized using R/Bioconductor package Lumi (Du et al., 2008) and further log2 transformed. This data was used in all subsequent analyses.

Western blotting (extended)

Cells were washed with $PBS^{(-/-)}$ twice and subsequently lysed with 2x Laemmli loading buffer (Sigma), containing 1 tablet of each complete protease inhibitor and PhosSTOP phosphatase inhibitor (Roche). DNA in samples were sheared with at least 27G syringe needles, heated at 65°C for 10 mins and resolved using sodium dodecyl sulphate polyacrylamide gel electrophoresis (SDS-PAGE). The iBlot® Dry Blotting System (Invitrogen) is used to transfer proteins onto a PVDF membrane using program P3 iBlot transfer device. Blots were rinsed in TBS and blocked in TBST (TBS + 0.05% Tween-20) containing either 3-5% bovine serum albumin (BSA, for western blotting, Cell Signalling Technologies) for phospho-protein detection or 5% skim dried milk (Diploma brand) at room temperature for ≥1 hour, probed with desired primary antibodies in blocking buffer at 4°C over-night. Membranes were then rinsed and washed thrice for 15 minutes each in TBST, probed with appropriate HRP-conjugated secondary antibodies in blocking buffer (typically 5% skim milk, normally at 1:1000 or higher dilutions) for at least one hour at room temperature, washed as before, rinsed in TBS and detected using Clarity ECL (BioRad) according to manufacturer's recommendations The images were analyzed using Image Lab (Bio-Rad) software.

Immunofluorescence and flow-cytometric (FACS) analyses

Staining with antigen-specific and fluorescent antibodies was performed essentially as described (Ovchinnikov et al., 2015; Ovchinnikov et al., 2014) with minor modifications. For staining with anti-phosphorylated proteins forms, PBS was substituted with TBS as a base buffer, and Triton-X100 was used at 0.25% for permeabilization in case of detection of a nuclear protein. All antibodies used in this study are listed below. For apoptosis measurement in neuronal cultures, 12 hours after hydrogen peroxide challenge we performed a TUNEL staining, a flow cytometry-optimized APO-BrdU™ TUNEL Assay Kit, with Alexa Fluor™ 488 Anti-BrdU (Invitrogen/ThermoFisherScientific) was used according to manufacturer's instructions (fractions of $TUNEL^{+}$ nuclei used for calculations shown in Figure 4A-C). Observations were validated using an externalised phosphoserine-visualizing surface Annexin V staining (using Dead Cell Apoptosis Kit with Annexin V Alexa Fluor™ 488 & Propidium Iodide, Invitrogen/ThermoFisherScientific) 4 hours after the hydrogen peroxide challenge according to manufacturer's instructions.

Statistical analyses

N refers to a number of independently-repeated experiments, n- technical replicates (e.g. wells in q qPCR reaction or a staining/differentiation). Unless stated otherwise, the results represent an analysis of means of at least three independent experiments. Mean-of-the-ratios (MoR) comparison approach was always taken, i.e. values from repeated experiments were compared after internal normalization(s) (e.g. for each individual western blot protein form of interest was normalized against a housekeeping gene and/or unphosphorylated tau levels, *etc*.). Two-tailed t-test for used for pairwise comparison of the means for different genotypes, and one-way ANOVA for identification of the presence of an outlier sample in a set of three or more cohorts. Standard symbols were used to reflect p values obtained through the respective statistical analyses (using GraphPad Prizm software 6 or 7): #, n.s. - not statistically-significant (p>0.05), * - p<0.05, ** - p<0.01, *** - p<0.001, **** - p<0.0001.

Morphometric measurements of cells in neurogenic cultures

Neuronal neurite length parameters, were measured using Harmony 12.1 software. As a first step, individual cell assignment was carried out using nuclear detection ("Method M", typically diameter 8-10 µm, splitting coefficient and common threshold ~25%) on nuclear stain channel. TUBB3-positive cells/processes (Figure S3I) were used for calculation of the dendritic parameters using built-in "neurite length analysis" module (CSIRO) using parameter settings determined via training on representative samples for each multi-sample experiment. Example of numeric settings for main parameters: Smoothing width 3, Linear window 6, Contrast 3, Diameter 6 Gap closure distance 5, Gap closure quality 25, Debarb length 20, Body thickening 4, Tree length 15.

Analysis of the effects of APP copy number on expression of HSA21 genes

Interestingly, a significant fraction (~30%) of genes affected by normalization of the *APP* copy number in DS (HSA21-trisomic) map to chromosome 21 (volcano plot in Figure 2D, see GEO data set for chromosomal assignment details). Particularly at day 65, the supernumerary *APP* copy has a profound effect on mis-expression of genes from HSA21 with 25 of 329 HSA21 genes (HugoGeneNomenclatureCommittee site-HSA21 gene annotation) overexpressed in an APP-dependent manner, while at day 45 only 4 HSA21 genes were overexpressed in an APP-dependent manner (Figure 2C). ENRICHR analysis reveals a significant overlap with gene sets involved in abnormal nervous system and abnormal brain morphology but not with Alzheimer's disease, as would perhaps be anticipated given that APP is thought to be an important determinant of AD-like neuropathology in DS. Collectively, our data suggest that even a modest increase in APP levels in a trisomy 21 context has profound effects on the transcriptional makeup of DS neurons, and that the impact of APP on gene expression increases with neuronal maturation, and has a particularly pronounced effect on over-expression of other HSA21 genes through an as yet unidentified mechanism (See GEO dataset for more details).

Identification and evaluation of putative epigenetic mechanisms mediating genome-wide supernumerary *APP* copy impact in Hsa21-trisomic DS iPS-derived neurons

To identify other potential mechanisms responsible for the observed APP-dependent genome-wide transcriptome changes we compared the 265 genes that are overexpressed in DS neurons in an APP-dependent fashion at both days with existing ChIP datasets (Lachmann et al., 2010), available through Enrichr online analysis tool suite (Chen et al., 2013; Kuleshov et al., 2016). This identified, at the high confidence levels (p<2E-8, Figure S4C), enrichment for targets of components of the polycomb repressive complex 2 (PRC2), such as SUZ12, EZH2, JARID2 and EED. Significantly, *SUZ12* and *JARID2,* both obligatory PRC2 components, are repressed in DS neurons in an APP copy number-dependent manner (see Figure S4C and Supplemental Table S4 for overlap gene memberships). How APP is mechanistically involved in this remains unclear and likely complex since a number of other epigenetic modifier genes were found to be upregulated in an APP-dependent and independent fashion (Figure S3J) in DS neurons. These include *HLCS*, a HSA21 gene implicated in modulation of repression-associated chromatin complexes (Zempleni et al., 2011; Zempleni et al., 2014) and *HMGN1*, a protein that associates with chromatin-accessible domains and enhancers and displaces heterochromatic histone H1 (Martinez de Paz and Ausio, 2016) (Figure S3J). Overexpression of APP in CRISPRa day 90 euploid neurons is sufficient to upregulate *HLCS* but not *HMGN1* (Figure S3K).

Comparison of neuronal gene expression datasets with previously reported data

Transcriptome comparison of our footprint free isogenic cohort of DS IPSC derived neurons with previously reported (Weick et al., 2016) 30 day-old retrovirally generated DS iPSC-derived neurons reveals substantial overlap in overexpressed HSA21 genes at day 45 (65%), and at day 65 (59%) (data not shown). However, when all up-regulated genes are considered, the overlap drops to 25% on day 45, and 12% on day 65 (data not shown), suggesting that while the core HSA21-driven overexpression cohort is largely preserved, neuronal cultures of different ages display highly-dynamic misexpression pattern of genes residing on other chromosomes. The limited overlap amongst the identified gene sets may also be due to platform differences, our more stringent cut-off used, the maturity of the cultures, or method of iPSC generation (footprint-free vs retroviral iPS cells. Comparison of our APP-dependent gene sets with a data set kindly provided by Wu et al. (Wu et al., 2016), who overexpressed APP in HEK293 cells, also did not show significant overlap, suggesting these gene regulatory effects of APP are likely to be cell context-dependent (Figure S4B). DEG overlap memberships are listed in Supplemental Table S4 and could be retrieved from GEO and Wu et al. resources online).

Oligonucleotides used in this study

qPCR primer sets

ETFA	TGTTGATGCTGGCTTTGTTC	TGGATGGCTCCAGATATTCC
APP	GATGCGGAGGAGGATGAC	TCTGTGGCTTCTTCGTAGG
NANOG	GATAGATTTCAGAGACAGAAATACCTCAGC	AGGCCTTCTGCGTCACACC
APP_{tot}	CATGGTCCAGAGTGGAAGCCATGGTC	GAGAGACTGATTCATGCGCTC
APP_{695}	AGGAGGAAGAAGTGGCTGAGGTGGA	TCTCTCGGTGCTTGGCCTCAA
APP_{751}	GCCGAGCAATGATCTCCCGCTGGTA	TCTCTCGGTGCTTGGCCTCAA
APP_{770}	GTGTGCTCTGAACAAGCCGAGAC	GGATCTCGGGCAAGAGGTTC
ASCL1	TCTCATCCTACTCGTCGGACGA	CTGCTTCCAAAGTCCATTCGCAC
DCX	TCCAGCAGCCAGCTCTCTACC	TACAGGTCCTTGTGCTTCCG
BACE1	ACCAACCTTCGTTTGCCCAA	TCTCCTAGCCAGAAACCATCAG
BACE2	GGAGATGCTGATCGGGACC	AGTACGTGTCTATGTAGGAGTGC
PAX6	CAGCACCAGTGTCTACCAACCA	CAGATGTGAAGGAGGAAACCG
GAPDH	GAGTCAACGGATTTGGTCGT	TTGATTTTGGAGGGATCTCG
GLAST	TACCCTTCGCTCAAAGTCACC	CCGATCTGGGCTGAGTCTTTT
GLI1	CCCAGTACATGCTGGTGGTT	GCTTTACTGCAGCCCTCGT
HB9	CGCTCTCCTACTCGTACCCG	GGAGTTGAAGTCGGGCATCTTA
HES1	GTGCGAGGGCGTTAATACCG	TGATCTGGGTCATGCAGTTGG
HOXB4	GCAAAGAGCCCGTCGTCTACCG	TGTCAGGTAGCGGTTGTA
NCAM	ACCTGGAGGACTTCTACCCG	ACCATGTGCCCATCCAGAGTC
NKX2.2	AACGATGAGGAGGGCTCTGTG	TGTCGCTGCTGTCGTAGAAGG
OLIG2	CACGGCCTACTCAAGTCTCCG	ACTTGGCGTCGGAGGTGAGG
OTX1	CTACCCTGACATCTTCATGCGGG	GAGAGGACTTCTTCTTGGCTG
OTX2	AAAGAAGACATCTCCAGCTCG	GTCGGGACTGAGGTGCTAGAGG
SOX1	TCCTGGAGTATGGACTGTCCG	GAATGCAGGCTGAATTCGG
SOX2	CCACCTACAGCATGTCCTACTCG	GGGAGGAAGAGGTAACCACAGG
SOD1	CTCACTCTCAGGAGACCATTGC	CCACAAGCCAAACGACTTCCAG
DSCR1	AAACTTCAGCAACCCCTTCTC	CCACTTGTTTCCATCCCACT
DYRK1A DO1	TTCAGTGGTGCCAATGAGGT	TTCTTGCTTTTGGTGCTTGGT
DYRK1A DO2	GCACACTGGAGAACCTCTGTT	AGCAGGTGGAATACCCAGAACT
S100B	TCCCGGGATGCGCCTGATCA	CCCCCAGAGCTGGCTCGGAT
SYNP	GCATCGACATAGCGTTTGCC	GACGAGGAGTCACCAATCAATG
MMP7	GAGTGAGCTACAGTGGGAACA	CTATGACGCGGGAGTTTAACAT
JUN	AACAGGTGGCACAGCTTAAAC	CAAACTGCTGCGTTAGCATGAG
HMGN1	GCGAAGCCGAAAAAGGCAG	TCCGCAGGTAAGTCTTCTTTAG

NAP1L3	GCAGTTAGAAATCGTGTGCAAG	TGTAGGCTCGTATTCTGCATTGA
H1FO	ACCCTCCTCGACTTCCACAG	TGTAGAGCTTGATAGCTGCCA
HLCS	CCCGTTGAGCATTATCACCTC	GGCAGACGCAAACTTGACTG
PRMT2	ACATTCCGGCAAACCATGTG	GGATGACTTTATCCGTCAGGG

CRISPR/SpCas9 manipulation of the *APP* locus used in this study

gRNA APP3.2	CACCGACCTGCATTGATACCAAGGA	AAACTCCTTGGTATCATGCAGGTC
SNA PCR APP3 435bp	CTGGGTGACAGAGTGAGGAAA	ACCCACTGATGGTAATGCTGG
Ca APPgRNA1	CACCGCGCGAGCGGGCGCAGTTCCC	AAACGGGAACTGCGCCCGCTCGCGC
Ca APPgRNA2	CACCGCCCACAGGTGCACGCGCCCT	AAACAGGGCGCGTGCACCTGTGGGC

Primers used for *APP* gene level manipulation using CRISPR/Cas9 technology

		Forward primer	Reverse primer
**Homology arm amplification**	**Left: 702 bp**	aaaaaggtaccGAACTTGACTTCTGCAATGACTGGT	Aaaaa ggcgcgccGCCTGGTCTTGAGGGAAGAAAAAG
	**Right: 793 bp**	aaaaaagcggccgcAAGCATCTAACAAGCCTCCACTG	aaaaaaccatggGGGGCTGTAGTTAGTTATAGGAGC
**Screening PCR**	**Left: 729 bp**	GTCCACAATCTCCACAGAC	AGGCCTACCCGCTTCCATTGCTC
**Puromycin-targeting probe PCR**	**483 bp**	ACCGAGTACAAGCCCACGGTGC	TCGTAGAAGGGGAGGTTGCGGG

Antibodies used in this study

Antibody	Recommended dilutiuon	Catalog#	Source
**APP (C-term)**	WB 1/1000, 5% CST-BSA	CST.2452S	CST
**GAPDH (D16H11) XP®**	WB 1/2000 NFDM block (be default)	CST.5174S	CST
**DYRK1A (D30C10) Rabbit mAb**	WB	sc-3010	SCBT
**Alzheimer's Disease Antibody Sampler Kit**	WB 1/1000 for pirmaries, 1/2500 for anti-Ms and Rb se	CST.9784S	CST
**Phospho-Tau (Ser396) (PHF13) Mouse mAb**	WB 1/1000, 5% CST-BSA	CST.9632S	CST
**β-Amyloid (pE3 Peptide) (D5N5H) Rabbit mAb**	WB:1/1000, IF: 1/200	CST.14975P	CST
**Phospho-Tau (Thr212) (PHF13) Mouse mAb**	WB:1/1000, 5% CST-BSA	MBS128512	CST
**BACE**	WB 1/1000 for pirmaries, 1/2500 for anti-Ms and Rb se	D10E5	CST
**AT270 (P-Tau Thr181)**	IF/WB	MN-1080	Thermo
**TUBB3 (Rabbit)**	IF/IB	SAB4300623	Sigma
**TUBB3 (Guinea pig)**	IF	302304	Synaptic Systems
**GFAP**	IF/WB	Z0334	Dako
**MC1**	1/250 (IF)	N/A	P. Davies
**AT8**	1/400 (IF)	MN1020	Thermo

Supplemental references

Briggs, J.A., Sun, J., Shepherd, J., Ovchinnikov, D.A., Chung, T.L., Nayler, S.P., Kao, L.P., Morrow, C.A., Thakar, N.Y., Soo, S.Y., *et al.* (2012). Integration-free induced pluripotent stem cells model genetic and neural developmental features of down syndrome etiology. Stem Cells *31*, 467-478.

Briggs, J.A., Sun, J., Shepherd, J., Ovchinnikov, D.A., Chung, T.L., Nayler, S.P., Kao, L.P., Morrow, C.A., Thakar, N.Y., Soo, S.Y., *et al.* (2013). Integration-free induced pluripotent stem cells model genetic and neural developmental features of down syndrome etiology. Stem Cells *31*, 467-478.

Chambers, S.M., Fasano, C.A., Papapetrou, E.P., Tomishima, M., Sadelain, M., and Studer, L. (2009). Highly efficient neural conversion of human ES and iPS cells by dual inhibition of SMAD signaling. Nature biotechnology *27*, 275-280.

Chen, E.Y., Tan, C.M., Kou, Y., Duan, Q., Wang, Z., Meirelles, G.V., Clark, N.R., and Ma'ayan, A. (2013). Enrichr: interactive and collaborative HTML5 gene list enrichment analysis tool. BMC Bioinformatics *14*, 128.

Du, P., Kibbe, W.A., and Lin, S.M. (2008). lumi: a pipeline for processing Illumina microarray. Bioinformatics *24*, 1547-1548.

Dumevska, B., Bosman, A., McKernan, R., Main, H., Schmidt, U., and Peura, T. (2016a). Derivation of Trisomy 21 affected human embryonic stem cell line Genea021. Stem cell research *16*, 401-404.

Dumevska, B., Bosman, A., McKernan, R., Schmidt, U., and Peura, T. (2016b). Derivation of human embryonic stem cell line Genea022. Stem cell research *16*, 472-475.

Huber, W., Carey, V.J., Gentleman, R., Anders, S., Carlson, M., Carvalho, B.S., Bravo, H.C., Davis, S., Gatto, L., Girke, T., *et al.* (2015). Orchestrating high-throughput genomic analysis with Bioconductor. Nature methods *12*, 115-121.

Kauffmann, A., Gentleman, R., and Huber, W. (2009). arrayQualityMetrics--a bioconductor package for quality assessment of microarray data. Bioinformatics *25*, 415-416.

Kuleshov, M.V., Jones, M.R., Rouillard, A.D., Fernandez, N.F., Duan, Q., Wang, Z., Koplev, S., Jenkins, S.L., Jagodnik, K.M., Lachmann, A., *et al.* (2016). Enrichr: a comprehensive gene set enrichment analysis web server 2016 update. Nucleic Acids Res *44*, W90-97.

Lachmann, A., Xu, H., Krishnan, J., Berger, S.I., Mazloom, A.R., and Ma'ayan, A. (2010). ChEA: transcription factor regulation inferred from integrating genome-wide ChIP-X experiments. Bioinformatics *26*, 2438-2444.

Letourneau, A., Santoni, F.A., Bonilla, X., Sailani, M.R., Gonzalez, D., Kind, J., Chevalier, C., Thurman, R., Sandstrom, R.S., Hibaoui, Y., *et al.* (2014). Domains of genome-wide gene expression dysregulation in Down's syndrome. Nature *508*, 345-350.

Martinez de Paz, A., and Ausio, J. (2016). HMGNs: The enhancer charmers. BioEssays : news and reviews in molecular, cellular and developmental biology *38*, 226-231.

Ovchinnikov, D.A., Hidalgo, A., Yang, S.K., Zhang, X., Hudson, J., Mazzone, S.B., Chen, C., Cooper-White, J.J., and Wolvetang, E.J. (2015). Isolation of contractile cardiomyocytes from human pluripotent stem-cell-derived cardiomyogenic cultures using a human NCX1-EGFP reporter. Stem cells and development *24*, 11-20.

Ovchinnikov, D.A., Titmarsh, D.M., Fortuna, P.R., Hidalgo, A., Alharbi, S., Whitworth, D.J., Cooper-White, J.J., and Wolvetang, E.J. (2014). Transgenic human ES and iPS reporter cell lines for identification and selection of pluripotent stem cells in vitro. Stem cell research *13*, 251-261.

Ritchie, M.E., Phipson, B., Wu, D., Hu, Y., Law, C.W., Shi, W., and Smyth, G.K. (2015). limma powers differential expression analyses for RNA-sequencing and microarray studies. Nucleic acids research *43*, e47.

Weick, J.P., Kang, H., Bonadurer, G.F., 3rd, and Bhattacharyya, A. (2016). Gene Expression Studies on Human Trisomy 21 iPSCs and Neurons: Towards Mechanisms Underlying Down's Syndrome and Early Alzheimer's Disease-Like Pathologies. Methods in molecular biology *1303*, 247-265.

Wu, Y., Zhang, S., Xu, Q., Zou, H., Zhou, W., Cai, F., Li, T., and Song, W. (2016). Regulation of global gene expression and cell proliferation by APP. Sci Rep *6*, 22460.

Zempleni, J., Li, Y., Xue, J., and Cordonier, E.L. (2011). The role of holocarboxylase synthetase in genome stability is mediated partly by epigenomic synergies between methylation and biotinylation events. Epigenetics *6*, 892-894.

Zempleni, J., Liu, D., Camara, D.T., and Cordonier, E.L. (2014). Novel roles of holocarboxylase synthetase in gene regulation and intermediary metabolism. Nutrition reviews *72*, 369-376.
